# Supplementary material for: Positive selection in the genomes of two Papua New Guinean populations at distinct altitude levels
Source: Nat Commun. 2024 Apr 30;15:3352. doi: 10.1038/s41467-024-47735-1 (PMC11061283; doi:10.1038/s41467-024-47735-1)
Supplement: Supplementary file 1 — Supplementary Information [file 41467_2024_47735_MOESM1_ESM.pdf]

Positive selection in the genomes of two Papua New Guinean populations at distinct altitude levels

Mathilde André[1,2], Nicolas Brucato[3], Georgi Hudjasov[2], Vasili Pankratov[2], Danat Yermakovich[2], Francesco Montinaro[1,4], Rita Kreevan[2], Jason Kariwiga[5,6], John Muke[7], Anne Boland[8], Jean-François Deleuze[8], Vincent Meyer[8], Nicholas Evans[9], Murray P. Cox[10,11], Matthew Leavesley[5,12,13], Michael Dannemann[2], Tõnis Org[2], Mait Metspalu[1], Mayukh Mondal[2,14*], François-Xavier Ricaut[3*]

*Corresponding authors:

mondal.mayukh@gmail.com

francois-xavier.ricaut@univ-tlse3.fr

These authors jointly supervised this work: Mayukh Mondal, François-Xavier Ricaut

Affiliations:

1. Estonian Biocentre, Institute of Genomics, University of Tartu, Riia 23b, 51010 Tartu, Tartumaa, Estonia
2. Centre for Genomics, Evolution & Medicine, Institute of Genomics, University of Tartu, Riia 23b, 51010 Tartu, Tartumaa, Estonia
3. Centre de Recherche sur la Biodiversité et l'Environnement (CRBE), Université de Toulouse, CNRS, IRD, Toulouse INP, Université Toulouse 3 – Paul Sabatier (UT3), Toulouse, France
4. Department of Biosciences, Biotechnology and the Environment, University of Bari, Bari, Italy
5. Strand of Anthropology, Sociology and Archaeology, School of Humanities and Social Sciences, University of Papua New Guinea, PO Box 320, University 134, National Capital District, Papua New Guinea
6. School of Social Science, University of Queensland, St Lucia, Queensland, Australia
7. Social Research Institute Ltd, Port Moresby, Papua New Guinea
8. Université Paris-Saclay, CEA, Centre National de Recherche en Génomique Humaine (CNRGH), 91057, Evry, France
9. ARC Centre of Excellence for the Dynamics of Language, Coombs Building, Fellows Road, CHL, CAP, Australian National University, Australia
10. School of Natural Sciences, Massey University, Palmerston North, New Zealand
11. Department of Statistics, University of Auckland, Auckland, New Zealand
12. College of Arts, Society and Education, James Cook University, P.O. Box 6811, Cairns, Queensland, 4870, Australia
13. ARC Centre of Excellence for Australian Biodiversity and Heritage, University of Wollongong, Wollongong, New South Wales, 2522, Australia
14. Institute of Clinical Molecular Biology, Christian-Albrechts-Universität zu Kiel 24118 Kiel, Germany

Table of contents

Supplementary Figures 3

Supplementary Tables 29

Supplementary Notes........

1. Sampling and sequencing 52
2. Phenotype data collection 53
3. Variant Calling 53
4. Quality control 54
5. Kinship analysis 54
6. Generation of genomic mask 55
7. LD pruning and Admixture 55
8. Phasing........ 56
9. Random sampling approach to test the significance of the top candidate regions for selection 56
10. Control for the difference in mean coverage. 57
11. Control for admixture in PNG lowlanders........ 57
12. Relate 58
13. Clues........ 59
14. Association in the UK Biobank........ 60
15. Association test with PNG phenotypes 60
16. Introgression........ 61

References: 62

Supplementary Figures

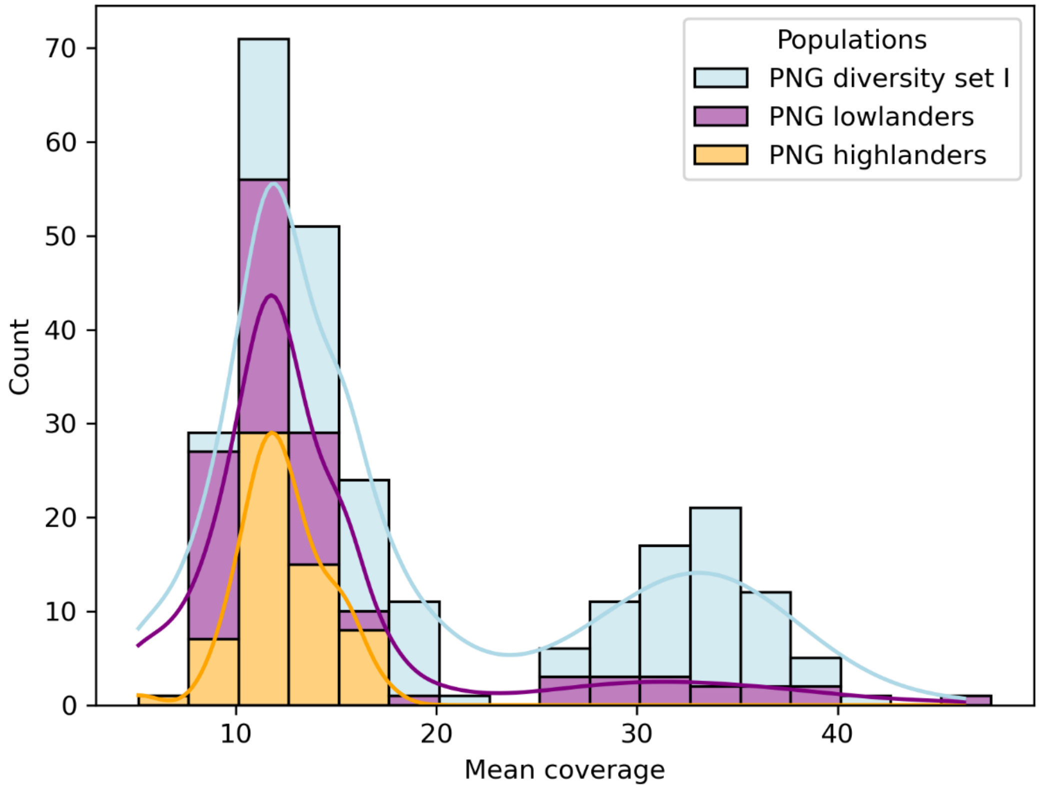

Figure S1: Distribution of mean coverage within the autosomes interval in Port Moresby (PNG diversity set I), PNG highlanders (Mt Wilhelm) and PNG lowlanders (Daru) sequences. Figure generated with the ggplot2 package (v3.4.2). Source data are provided as a Source Data file.

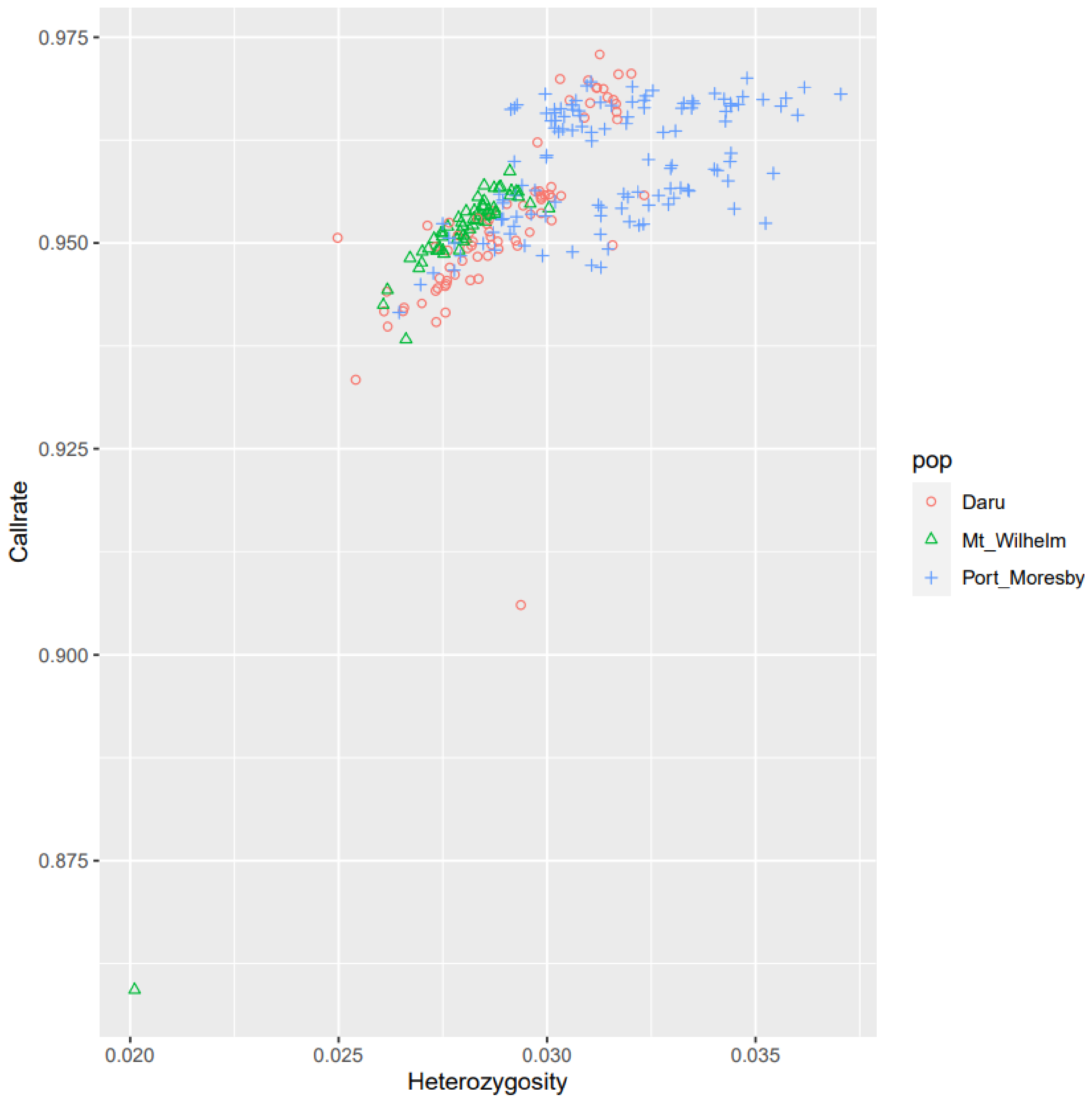

Figure S2: Heterozygosity to call rate within autosome interval in PNG diversity set I (Port Moresby), PNG highlanders(Mt Wilhelm), PNG lowlanders (Daru) sequences. Figure generated with the ggplot2 package (v3.4.2). Source data are provided as a Source Data file.

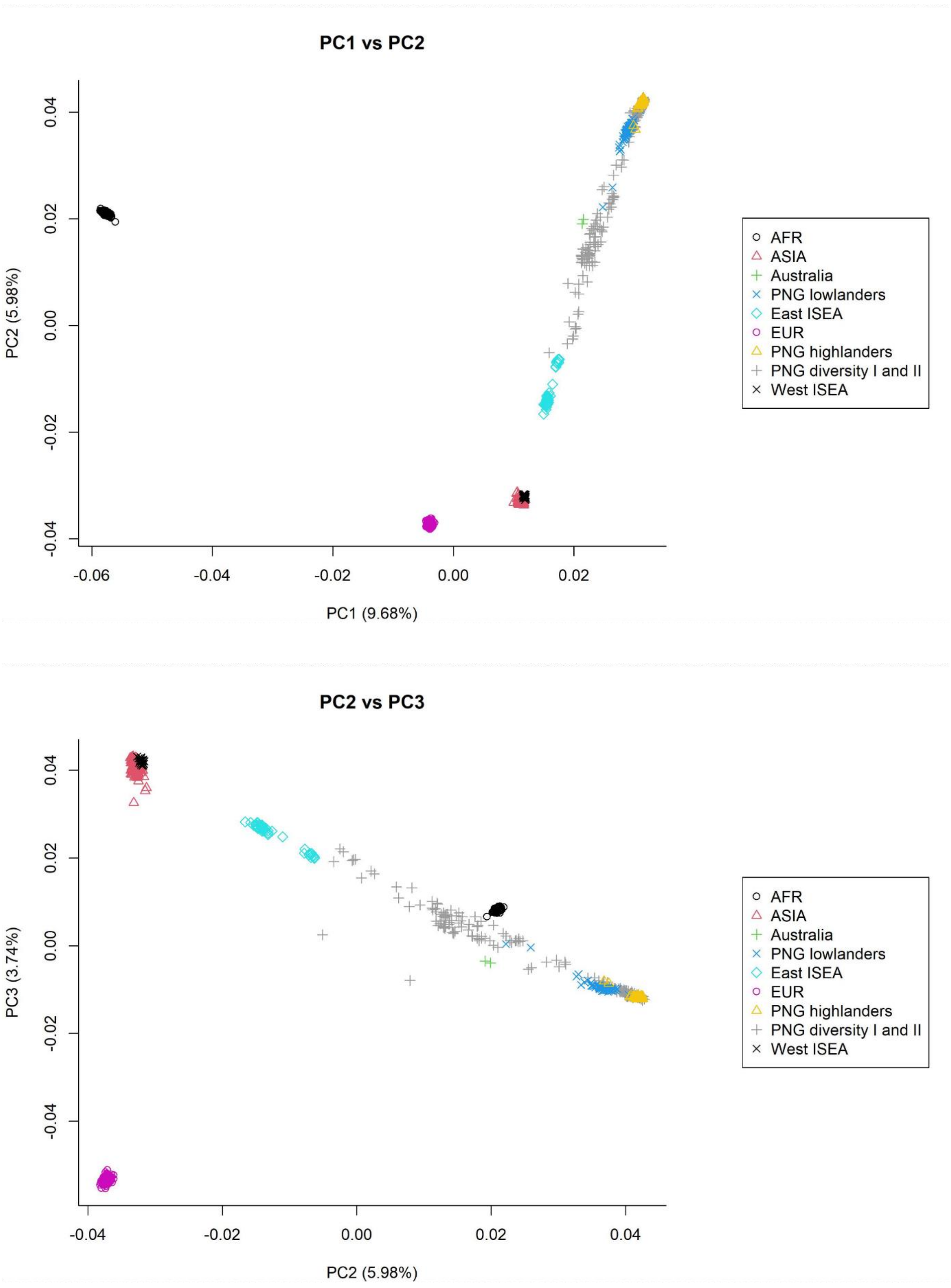

Figure S3: PCA plots fromPC1 to PC3. AFR=YRI and ESN, ASIA=CHB and KHV, EUR= GBR and CEU, all from the 1000 Genomes Project (Byrska-Bishop et al., 2022), PNG diversity I and II (Brucato et al., 2021, Bergström et al., 2020; Jacobs et al., 2019; Malaspinas et al., 2016; Mallick et al., 2016; Vernot et al., 2016), West and East ISEA from Jacobs et al., 2019 and AUS = Aboriginal Australian genomes from SGDP (Mallick et al. 2016). The figure was generated using R v3.3.0 software and the ggplot2 package (v3.4.2). Source data are provided as a Source Data file.

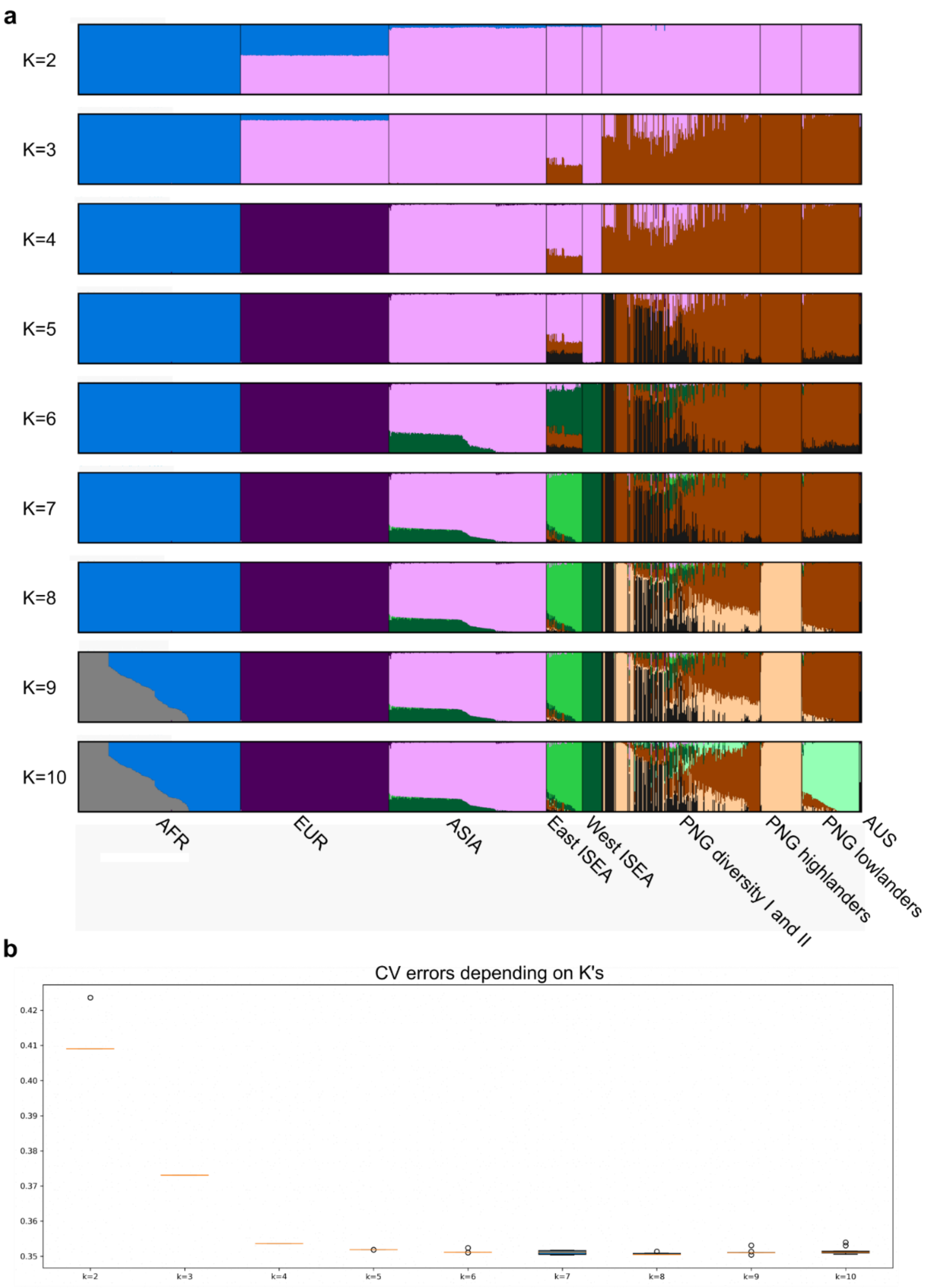

Figure S4: (**a**) **ADMIXTURE ancestry proportions for K=2 to K=10**. AFR=YRI and ESN, ASIA=CHB and KHV, EUR= GBR and CEU, all from the 1000 Genomes Project (Byrska-Bishop et al., 2022), PNG diversity I and II (Brucato et al., 2021, Bergström et al., 2020; Jacobs et al., 2019; Malaspinas et al., 2016; Mallick et al., 2016; Vernot et al., 2016), West and East ISEA from Jacobs et al., 2019 and AUS = Aboriginal Australian genomes from SGDP (Mallick et al. 2016). Plots generated with pong (v 1.5) (Behr et al., 2016) (**b**)**. Cross-validation errors for ADMIXTURE.** CV errors were computed for ten independent runs of the ADMIXTURE analysis from K=2 to K=10. CV error is low in all K but 2 and 3, with K=8 showing the lowest CV error (CV error mean [95% CI]:0.351 [0.350 - 0.351]). Source data are provided as a Source Data file.

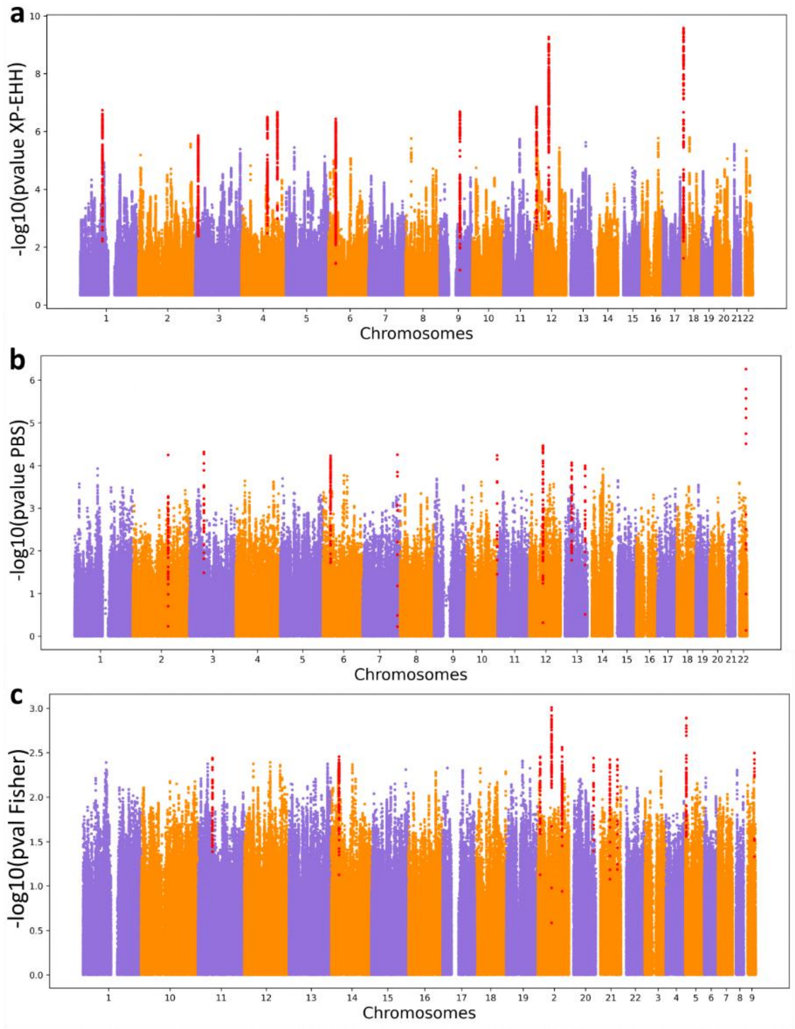

Fig. S5: Manhattan plots of the p-value for the three selection scans among PNG highlanders computed with a random sampling approach. The ten genomic regions with the highest score are shown in red. (**a**) P-values for the XP-EHH scores using PNG highlanders as the target population and PNG lowlanders as the reference population. (**b**) P-values for the PBS scores using PNG highlanders as the target population, PNG lowlanders as the reference population, and Yorubas from 1000G as the outgroup. (**c**) P-values for the Fisher Scores combining the PBS and XP-EHH scores of PNG highlanders. Source data are provided as a Source Data file.

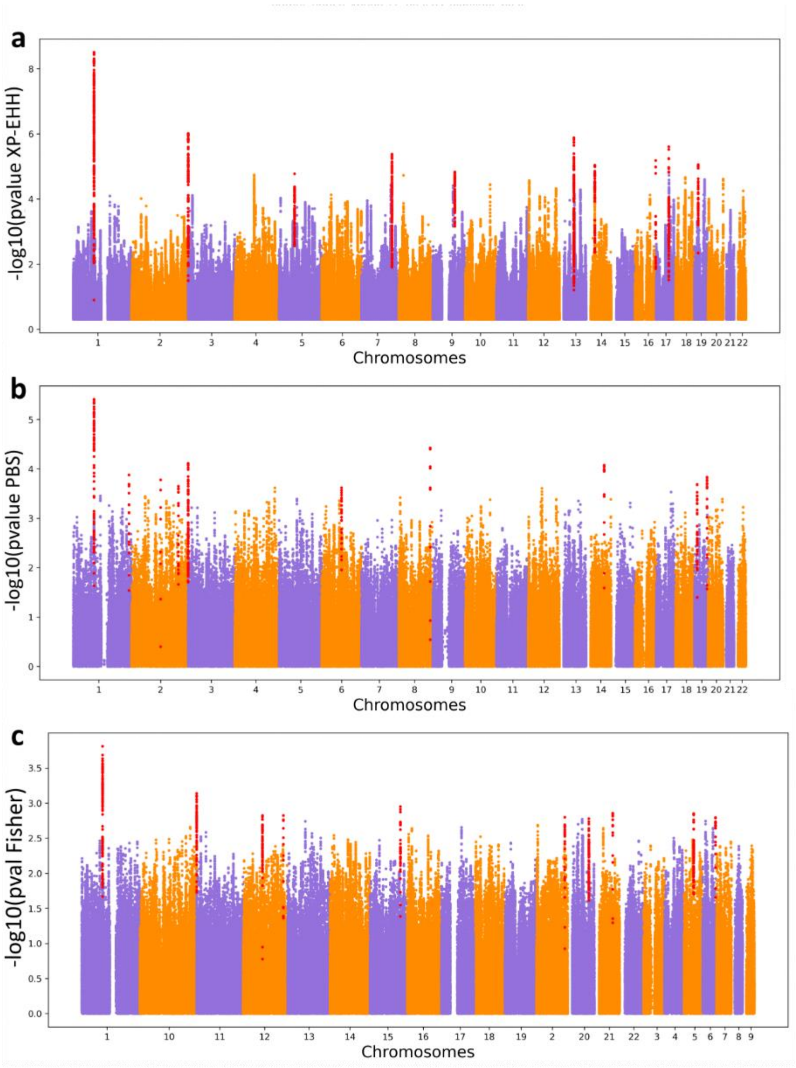

Fig. S6: Manhattan plots of the p-value for the three selection scans among PNG lowlanders computed by random sampling approach. The ten genomic regions with the highest score are shown in red. (**a**) P-values for the XP-EHH scores using PNG lowlanders as the target population and PNG highlanders as the reference population. (**b**) P-values for the PBS scores using PNG lowlanders as the target population, PNG highlanders as the reference population, and Yorubas from 1000G as the outgroup. (**c**) P-values for the Fisher Scores combining the PBS and XP-EHH scores of PNG lowlanders. Source data are provided as a Source Data file.

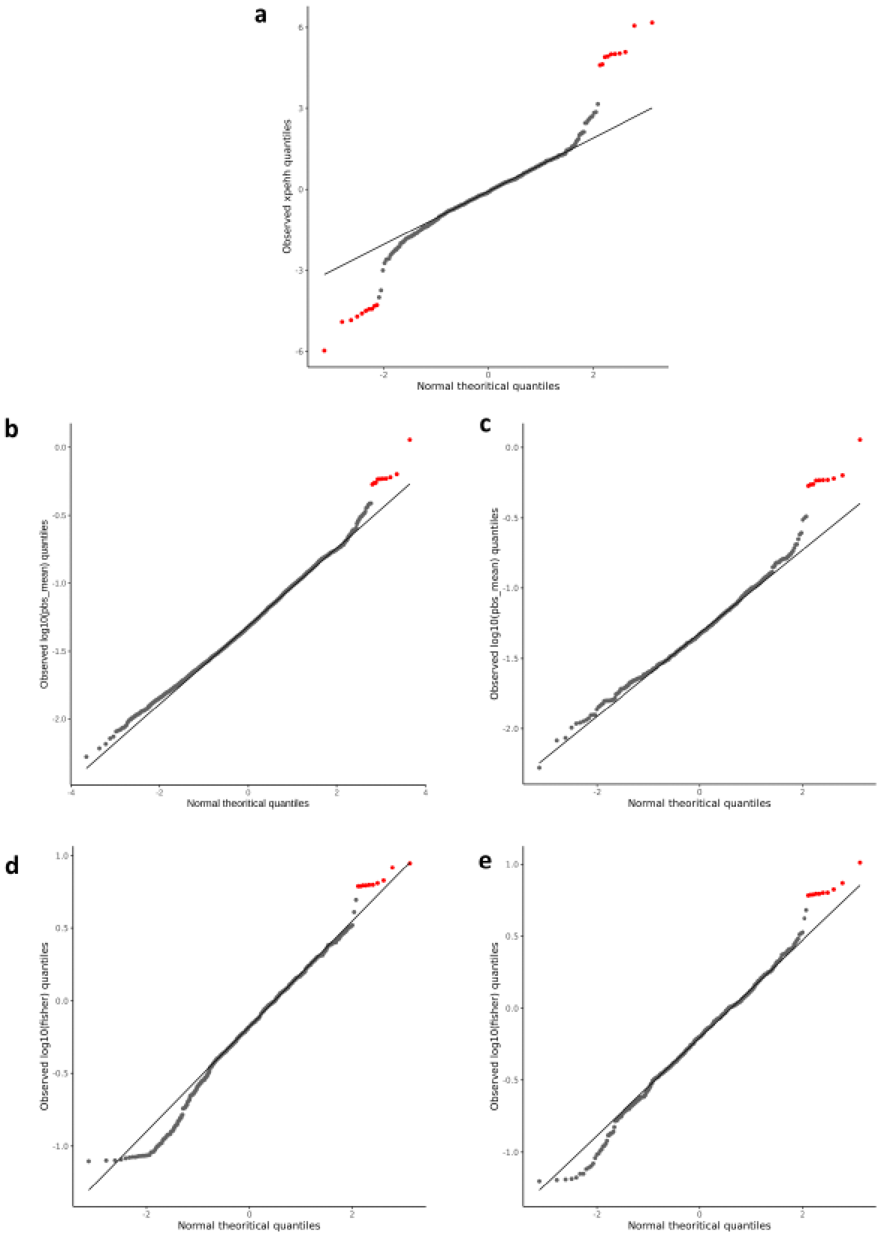

Figure S7: Quantile-quantile plot for the XP-EHH, PBS and Fisher scores computed for PNG highlanders and lowlanders. (**a**) QQ plot showing the observed XP-EHH score of independent random SNPs separated by 5MB (y-axis) as a function of quantiles expected from a normal distribution with the same mean and variance as the empirical distribution(x-axis). SNPs with the highest score within the top 10 XP-EHH regions in PNG highlanders (Table S7) and PNG lowlanders (Table S8) are shown in red. (**b,c**) QQ plot showing the observed log10(mean PBS score) of independent random 20 SNPs long windows separated by 5MB (y-axis) as a function of quantiles expected from a normal distribution with the same mean and variance as the empirical distribution(x-axis). 20 SNPs windows with the highest mean PBS score within the top 10 PBS regions in PNG highlanders (**b**, Table S7) and PNG lowlanders (**c**, Table S8) are shown in red. (**d,e**) QQ plot showing the observed log10(Fisher score) of independent random 20 SNPs long windows separated by 5MB (y-axis) as a function of quantiles expected from a normal distribution with the same mean and variance as the empirical distribution(x-axis). 20 SNPs windows with the highest Fisher score within the top 10 Fisher regions in PNG highlanders (**d**, Table S7) and PNG lowlanders (**e**, Table S8) are shown in red. Source data are provided as a Source Data file.

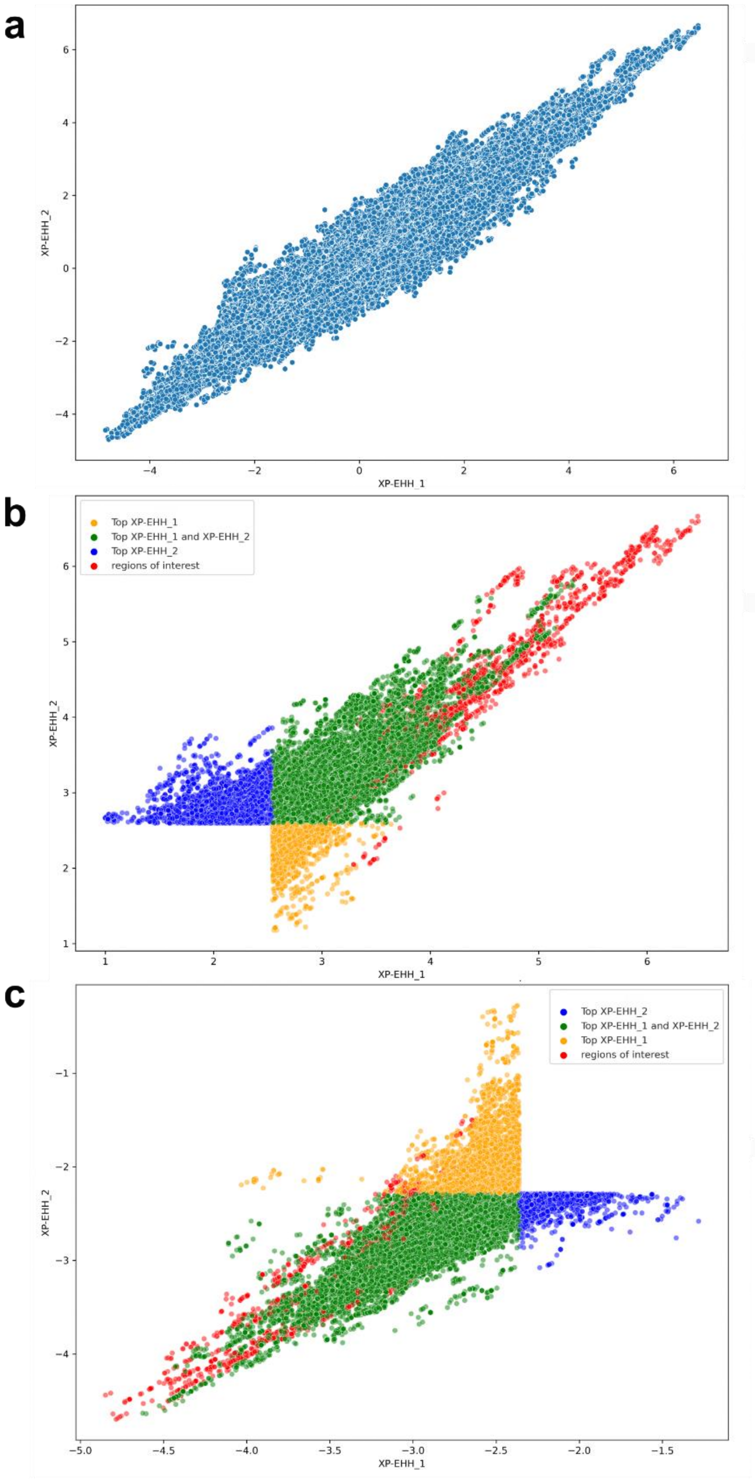

Figure S8: XP-EHH results with high or reduced coverage PNG lowlanders. (**a**) Correlation between variant calling including high coverage PNG lowlanders (XP-EHH_1) and variant calling with reduced coverage (XP-EHH_2). Spearman coefficient=0.970. (**b**) zoom in on the SNPs in the 99th percentile (top selected SNPs in PNG highlanders) for XP-EHH_1, XP-EHH_2 or both. SNPs included in the top XP-EHH regions in PNG highlanders (Table S7) are coloured in red. (**c**) zoom in on the SNPs in the 1st percentile (top selected SNPs in PNG lowlanders) for XP-EHH_1, XP-EHH_2 or both. SNPs included in the top XP-EHH regions in PNG lowlanders (Table S8) are coloured in red. Source data are provided as a Source Data file.

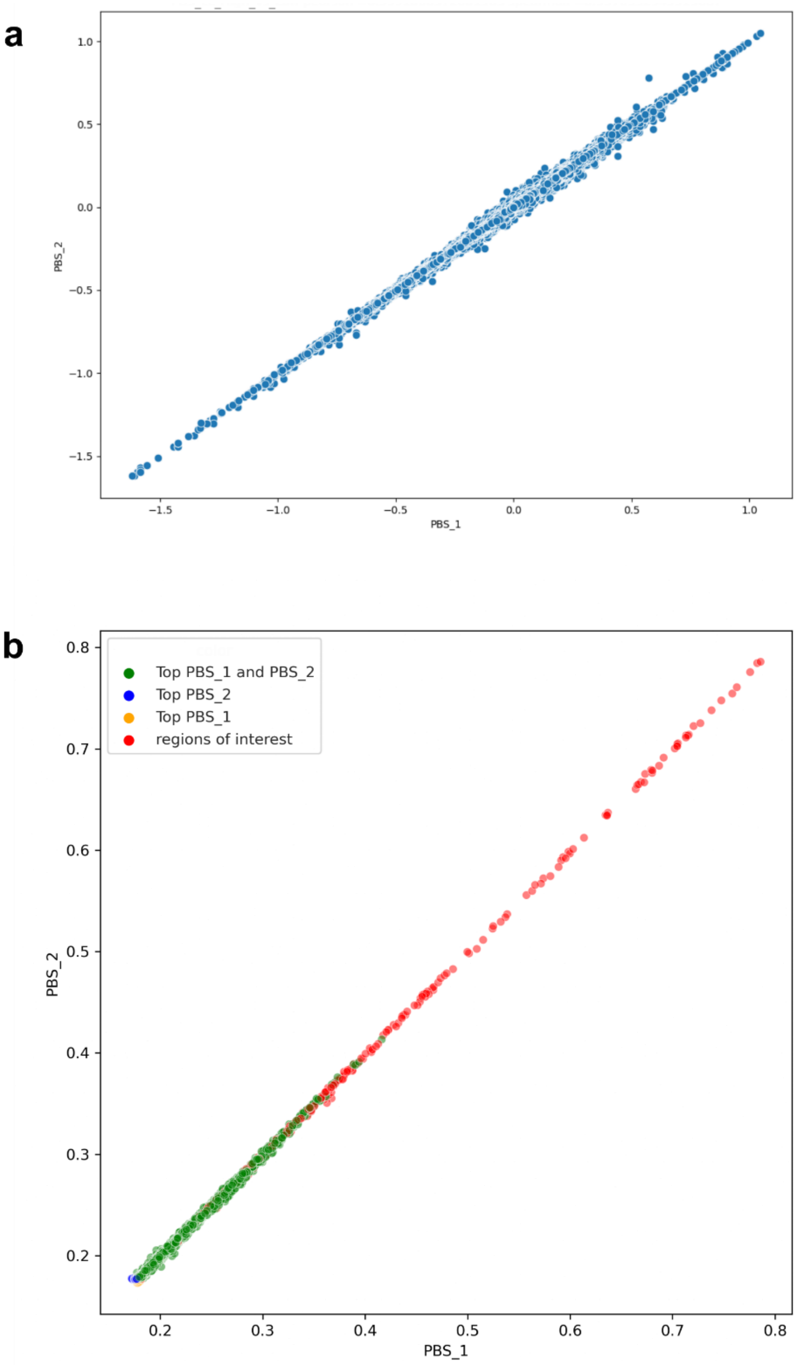

Figure S9: PBS results with high or low coverage PNG lowlanders as the target population. PNG highlanders are used as the reference population, and YRI from the 1000 Genomes Project as the outgroup. (**a**) Correlation between variant calling including high coverage PNG lowlanders (PBS_1) and variant calling with reduced coverage (PBS_2). Spearman coefficient=0.998. (**b**) zoom in on the SNPs in the 99th percentile (top selected SNPs in PNG lowlanders) for PBS_1, PBS_2, or both. SNPs included in the top PBS regions in PNG lowlanders (Table S8) are coloured in red. Source data are provided as a Source Data file.

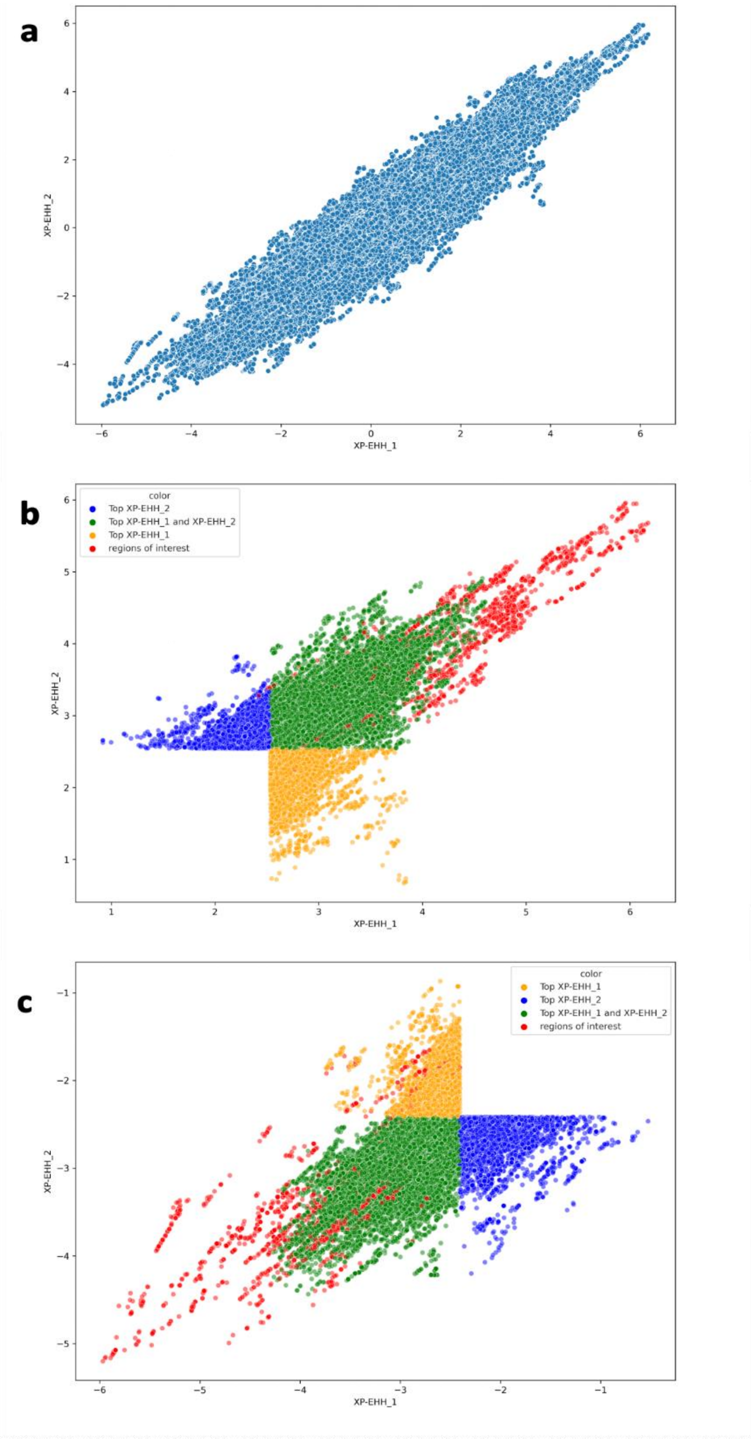

Figure S10: XP-EHH results with or without admixed PNG lowlanders. (**a**) Correlation between XP-EHH score including admixed PNG lowlanders (XP-EHH_1) and XP-EHH score not including admixed PNG lowlanders (XP-EHH_2) as the reference population. Spearman coefficient=0.950. (**b**) zoom in on the SNPs in the 99th percentile (top selected SNPs in PNG highlanders) for XP-EHH_1, XP-EHH_2 or both. SNPs included in the top XP-EHH regions in PNG highlanders (Table S7) are coloured in red. (**c**) zoom in on the SNPs in the 1st percentile (top selected SNPs in PNG lowlanders) for XP-EHH_1, XP-EHH_2 or both. SNPs included in the top XP-EHH regions in PNG lowlanders (Table S8) are coloured in red. Source data are provided as a Source Data file.

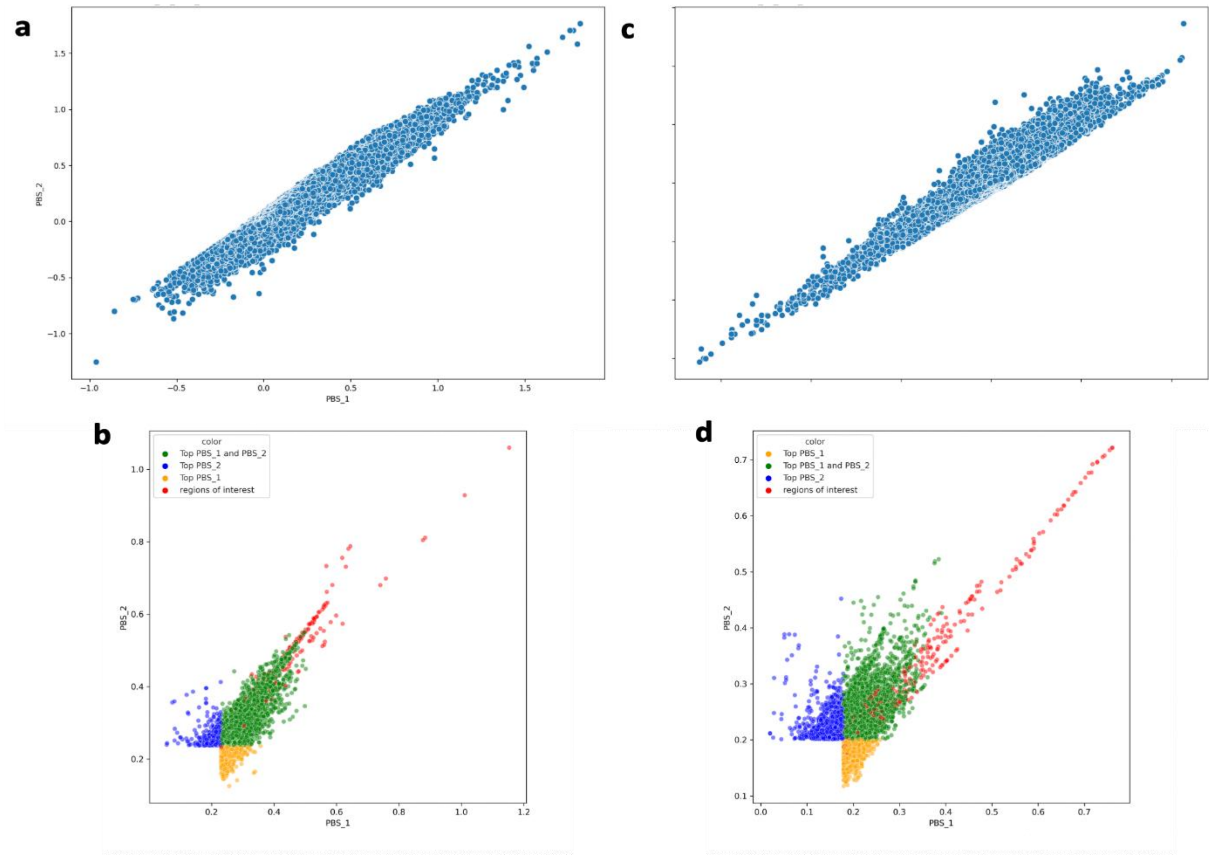

Figure S11: PBS results with or without admixed PNG lowlanders. (**a**) Correlation between PBS results for PNG highlanders with PNG lowlanders including admixed PNG lowlanders as the reference population (PBS_1), PBS results for PNG highlanders with PNG lowlanders not including admixed PNG lowlanders as the reference population (PBS_2) and YRI as the outgroup. Spearman coefficient=0.935. (**b**) zoom in on the SNPs in the 99th percentile (top selected SNPs in PNG highlanders) for PBS_1, PBS_2, or both. SNPs included in the top PBS regions in PNG highlanders (Table S8) are coloured in red. (**c**) Correlation between PBS results for PNG lowlanders including admixed PNG lowlanders (PBS_1) and PBS results for PNG lowlanders not including admixed PNG lowlanders (PBS_2) with PNG highlanders as the reference population and YRI from 1000 as the outgroup. Spearman coefficient=0.926. (**d**) zoom in on the SNPs in the 99th percentile (top selected SNPs in PNG lowlanders) for PBS_1, PBS_2, or both. SNPs included in the top PBS regions in PNG lowlanders (Table S8) are coloured in red. Source data are provided as a Source Data file.

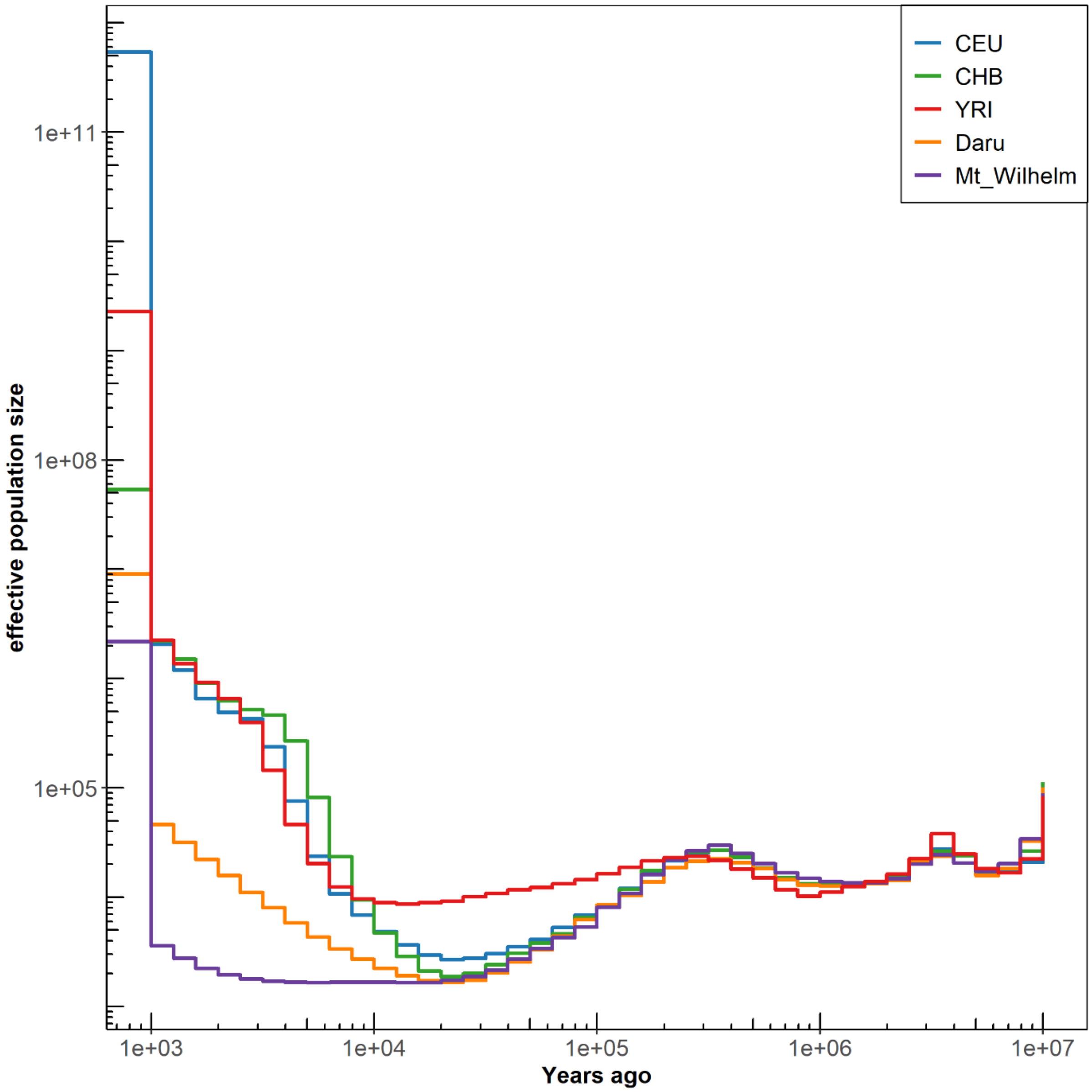

Figure S12: Relate effective population size curves computed with Relate for CEU, CHB, and YRI from the 1000 Genomes Project, Mt Wilhelm (PNG highlanders) and Daru (PNG lowlanders). Figure generated with the ggplot2 package (v3.4.2). Source data are provided as a Source Data file.

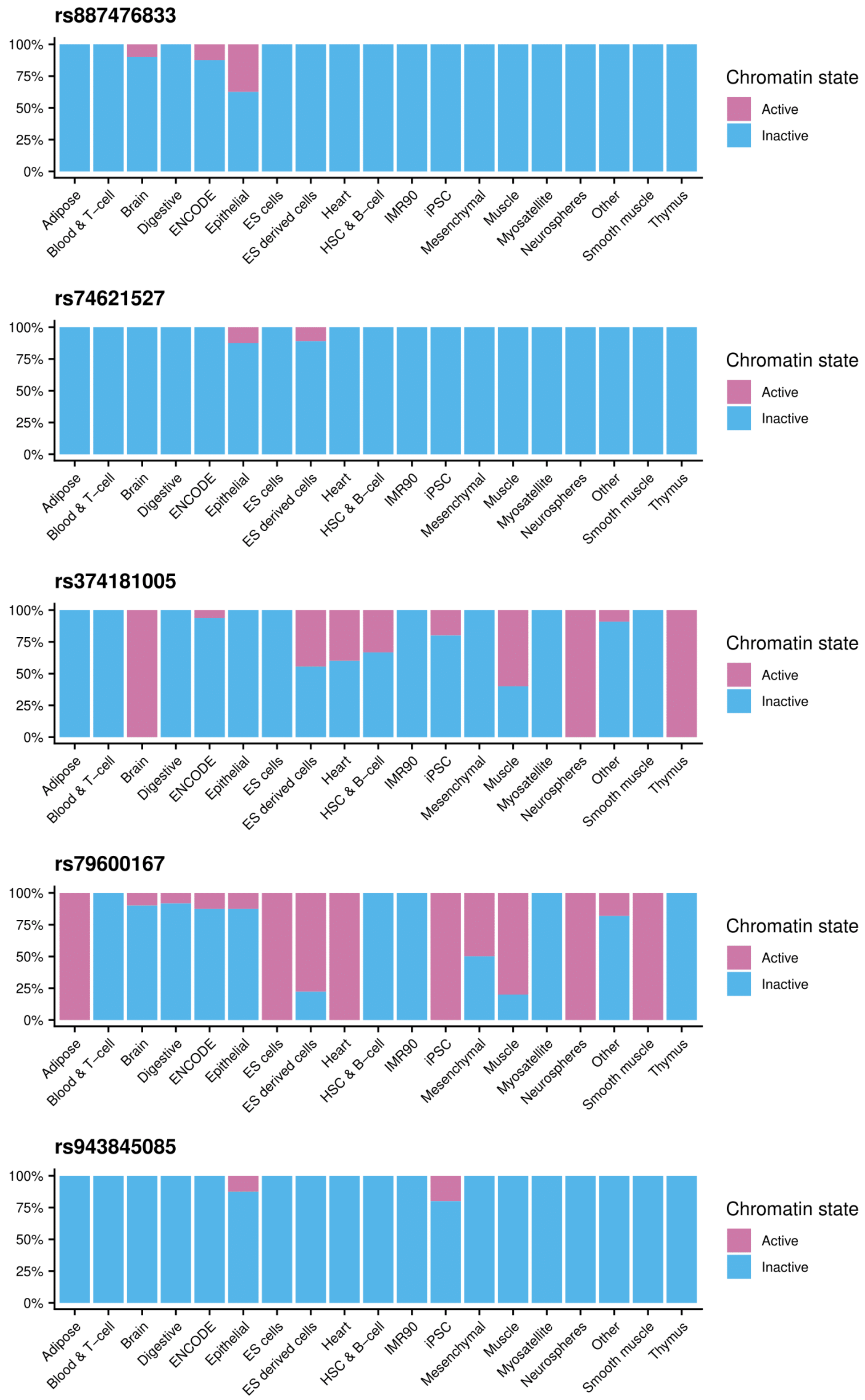
rs887476833
rs74621527
rs374181005
rs79600167
rs943845085
100%
75%
50%
25%
0%
Adipose
Blood & T−cell
Brain
Digestive
ENCODE
Epithelial
ES cells
ES derived cells
Heart
HSC & B−cell
IMR90
iPSC
Mesenchymal
Muscle
Myosatellite
Neurospheres
Other
Smooth muscle
Thymus
Chromatin state
Active
Inactive

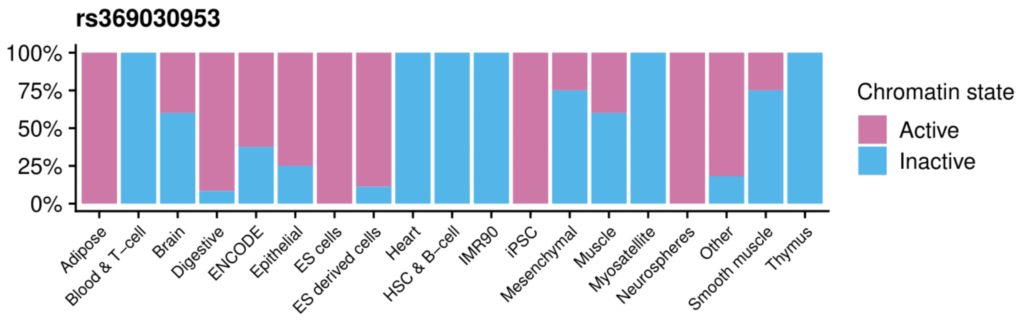
rs369030953
100%
75%
50%
25%
0%
Adipose
Blood & T–cell
Brain
Digestive
ENCODE
Epithelial
ES cells
ES derived cells
Heart
HSC & B–cell
IMR90
iPSC
Mesenchymal
Muscle
Myosatellite
Neurospheres
Other
Smooth muscle
Thymus
Chromatin state
Active
Inactive

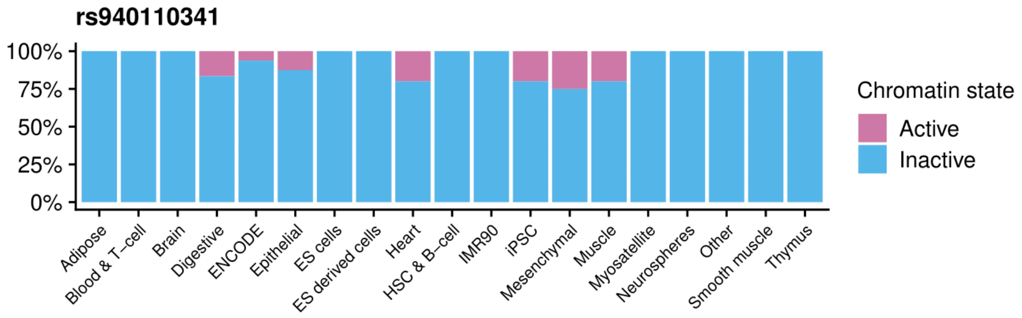
rs940110341
100%
75%
50%
25%
0%
Adipose
Blood & T–cell
Brain
Digestive
ENCODE
Epithelial
ES cells
ES derived cells
Heart
HSC & B–cell
IMR90
iPSC
Mesenchymal
Muscle
Myosatellite
Neurospheres
Other
Smooth muscle
Thymus
Chromatin state
Active
Inactive

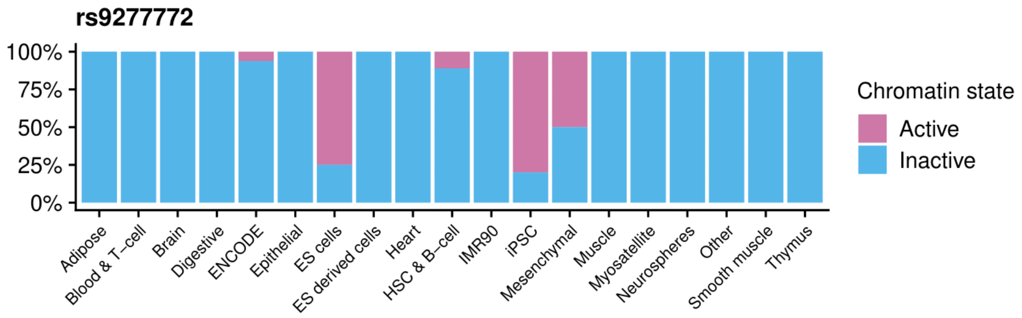
rs9277772
100%
75%
50%
25%
0%
Adipose
Blood & T–cell
Brain
Digestive
ENCODE
Epithelial
ES cells
ES derived cells
Heart
HSC & B–cell
IMR90
iPSC
Mesenchymal
Muscle
Myosatellite
Neurospheres
Other
Smooth muscle
Thymus
Chromatin state
Active
Inactive

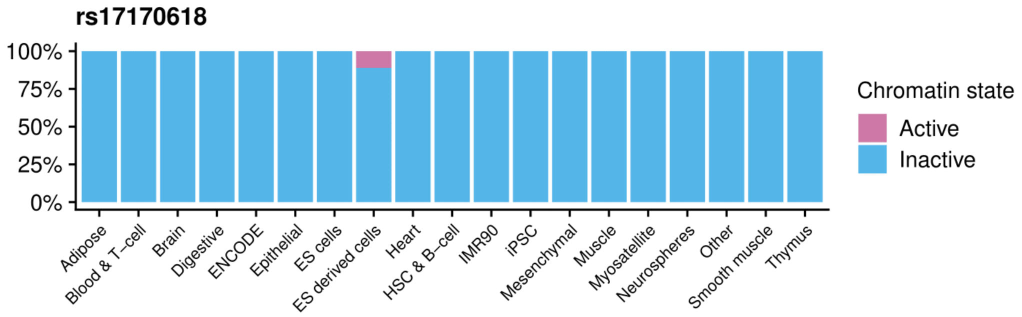
rs17170618
100%
75%
50%
25%
0%
Adipose
Blood & T–cell
Brain
Digestive
ENCODE
Epithelial
ES cells
ES derived cells
Heart
HSC & B–cell
IMR90
iPSC
Mesenchymal
Muscle
Myosatellite
Neurospheres
Other
Smooth muscle
Thymus
Chromatin state
Active
Inactive

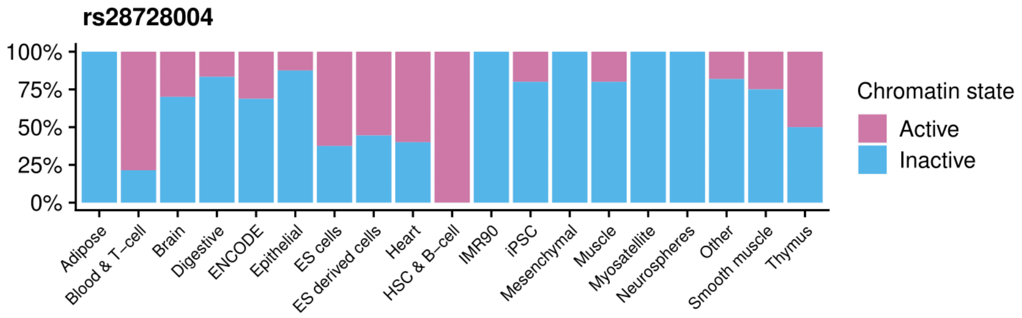
rs28728004
100%
75%
50%
25%
0%
Adipose
Blood & T–cell
Brain
Digestive
ENCODE
Epithelial
ES cells
ES derived cells
Heart
HSC & B–cell
IMR90
iPSC
Mesenchymal
Muscle
Myosatellite
Neurospheres
Other
Smooth muscle
Thymus
Chromatin state
Active
Inactive

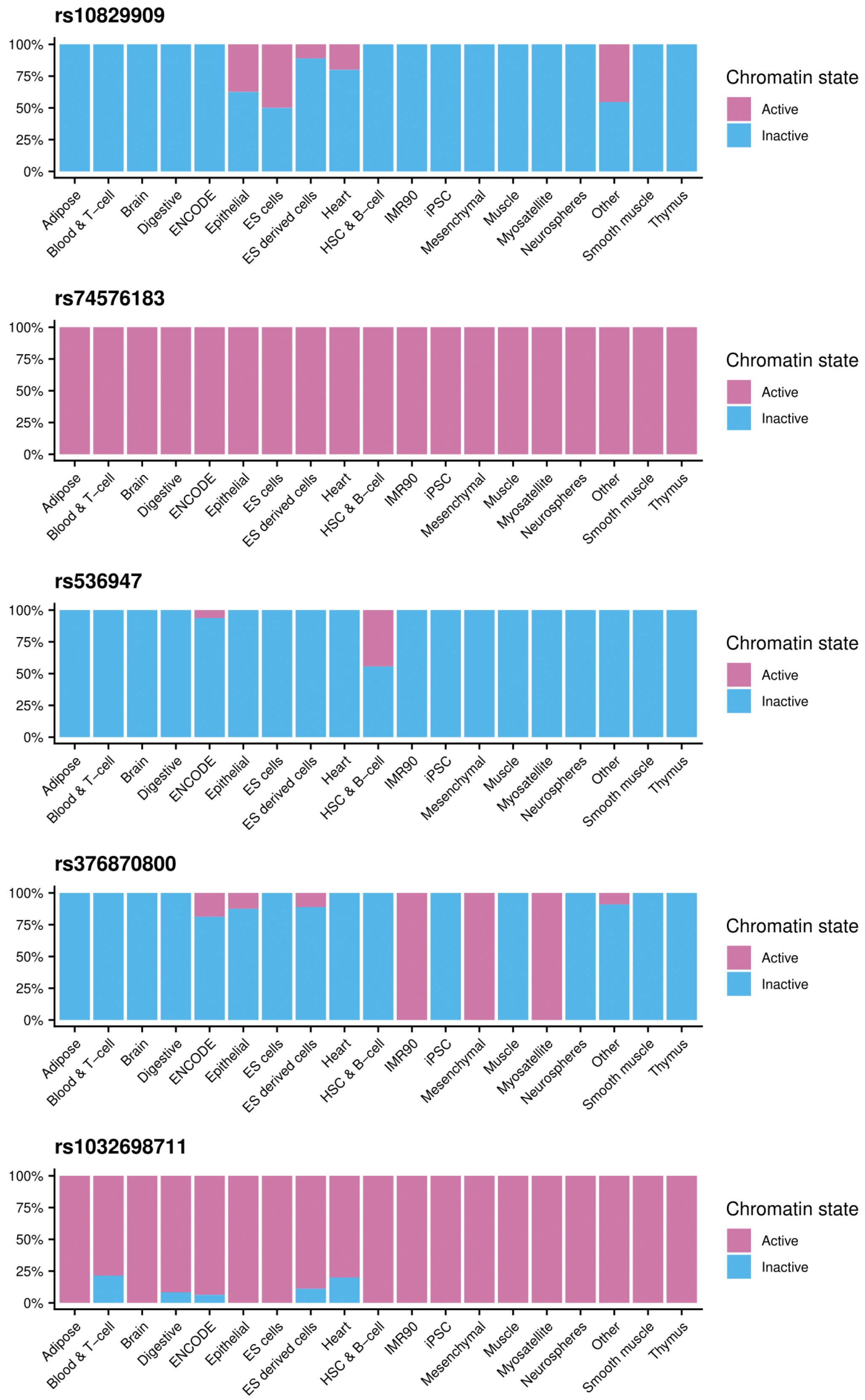
rs10829909
rs74576183
rs536947
rs376870800
rs1032698711
100%
75%
50%
25%
0%
Adipose
Blood & T−cell
Brain
Digestive
ENCODE
Epithelial
ES cells
ES derived cells
Heart
HSC & B−cell
IMR90
iPSC
Mesenchymal
Muscle
Myosatellite
Neurospheres
Other
Smooth muscle
Thymus
Chromatin state
Active
Inactive

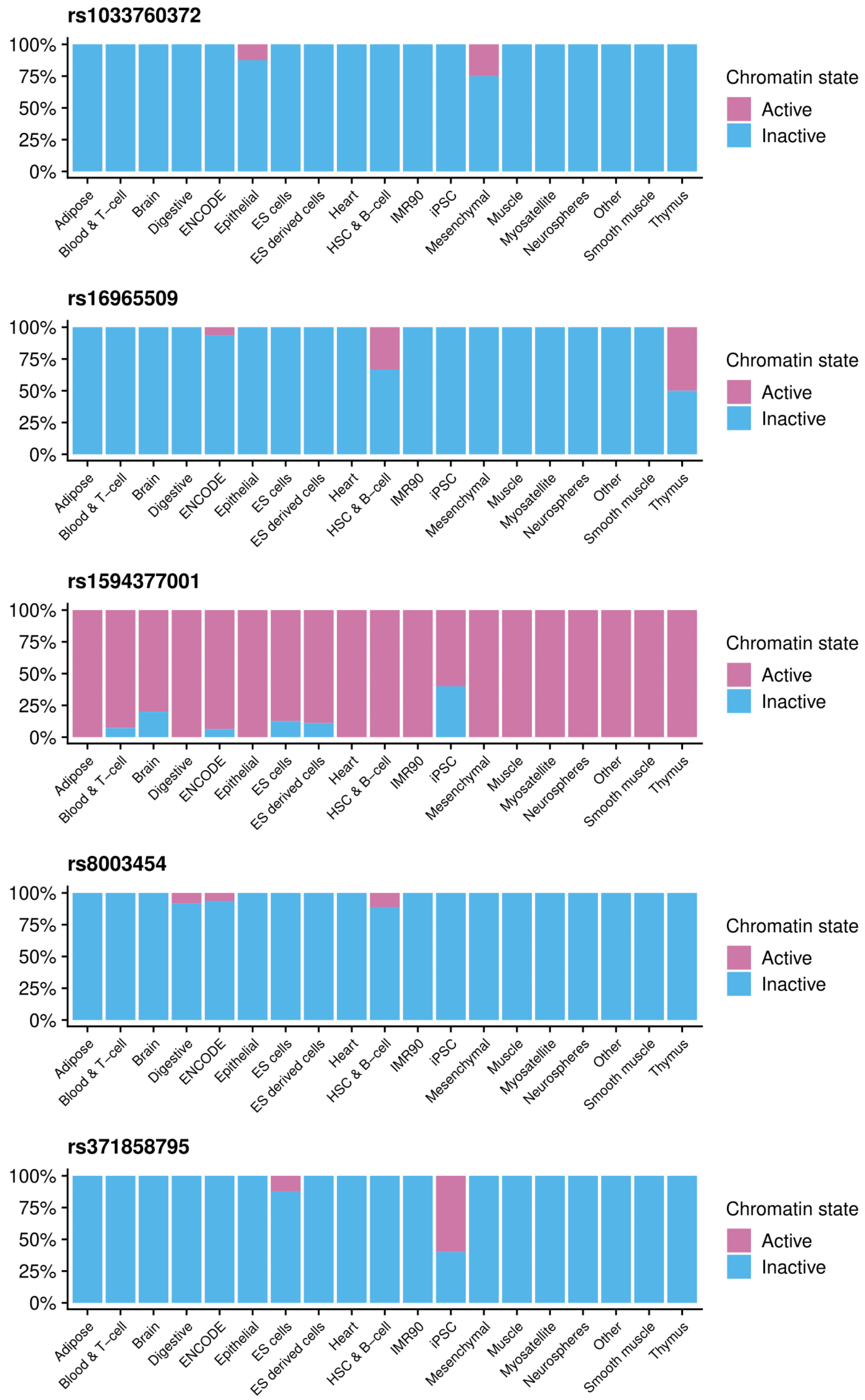
rs1033760372
rs16965509
rs1594377001
rs8003454
rs371858795
100%
75%
50%
25%
0%
Adipose
Blood & T−cell
Brain
Digestive
ENCODE
Epithelial
ES cells
ES derived cells
Heart
HSC & B−cell
IMR90
iPSC
Mesenchymal
Muscle
Myosatellite
Neurospheres
Other
Smooth muscle
Thymus
Chromatin state
Active
Inactive

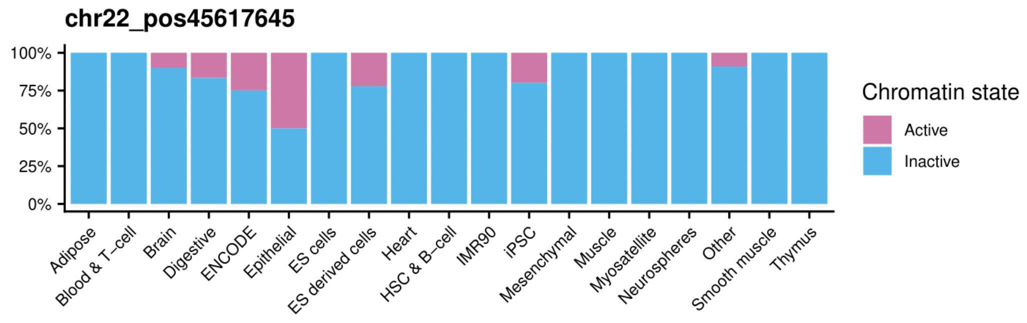

Figure S13: Chromatin state of the candidate SNPs of PNG highlanders for the epigenomes of the Roadmap project clustered in tissues. Figure generated with the ggplot2 package (v3.4.2).

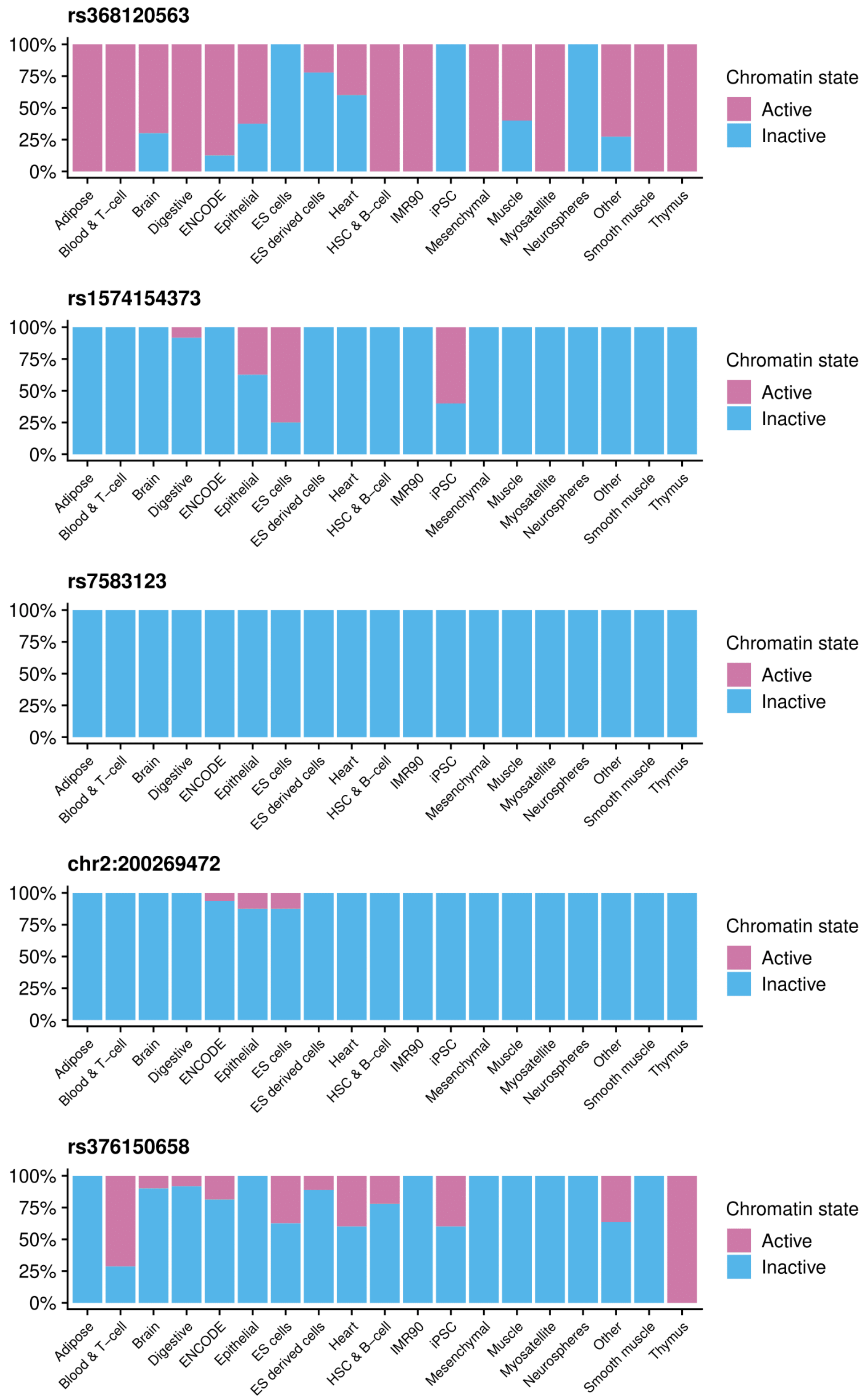
rs368120563
rs1574154373
rs7583123
chr2:200269472
rs376150658
100%
75%
50%
25%
0%
Adipose
Blood & T−cell
Brain
Digestive
ENCODE
Epithelial
ES cells
ES derived cells
Heart
HSC & B−cell
IMR90
iPSC
Mesenchymal
Muscle
Myosatellite
Neurospheres
Other
Smooth muscle
Thymus
Chromatin state
Active
Inactive

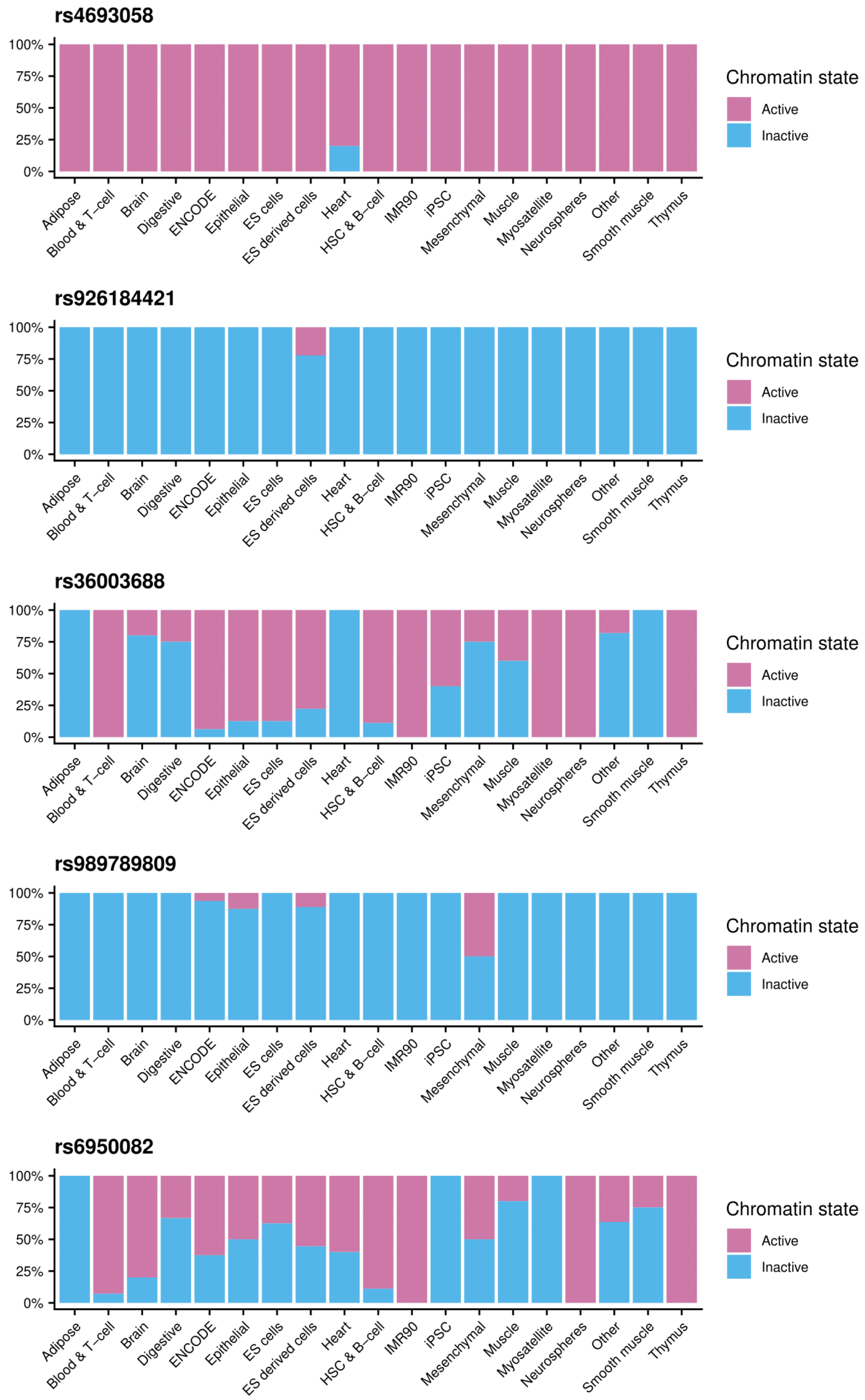
rs4693058
100%
75%
50%
25%
0%
Adipose
Blood & T−cell
Brain
Digestive
ENCODE
Epithelial
ES cells
ES derived cells
Heart
HSC & B−cell
IMR90
iPSC
Mesenchymal
Muscle
Myosatellite
Neurospheres
Other
Smooth muscle
Thymus
Chromatin state
Active
Inactive
rs926184421
100%
75%
50%
25%
0%
Adipose
Blood & T−cell
Brain
Digestive
ENCODE
Epithelial
ES cells
ES derived cells
Heart
HSC & B−cell
IMR90
iPSC
Mesenchymal
Muscle
Myosatellite
Neurospheres
Other
Smooth muscle
Thymus
Chromatin state
Active
Inactive
rs36003688
100%
75%
50%
25%
0%
Adipose
Blood & T−cell
Brain
Digestive
ENCODE
Epithelial
ES cells
ES derived cells
Heart
HSC & B−cell
IMR90
iPSC
Mesenchymal
Muscle
Myosatellite
Neurospheres
Other
Smooth muscle
Thymus
Chromatin state
Active
Inactive
rs989789809
100%
75%
50%
25%
0%
Adipose
Blood & T−cell
Brain
Digestive
ENCODE
Epithelial
ES cells
ES derived cells
Heart
HSC & B−cell
IMR90
iPSC
Mesenchymal
Muscle
Myosatellite
Neurospheres
Other
Smooth muscle
Thymus
Chromatin state
Active
Inactive
rs6950082
100%
75%
50%
25%
0%
Adipose
Blood & T−cell
Brain
Digestive
ENCODE
Epithelial
ES cells
ES derived cells
Heart
HSC & B−cell
IMR90
iPSC
Mesenchymal
Muscle
Myosatellite
Neurospheres
Other
Smooth muscle
Thymus
Chromatin state
Active
Inactive

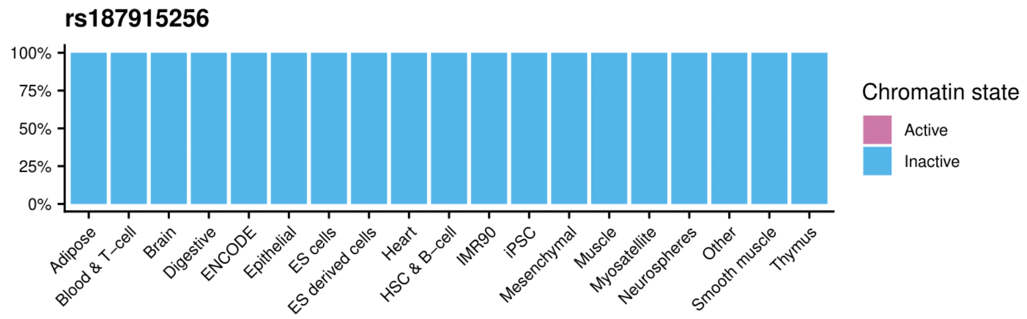
rs187915256
100%
75%
50%
25%
0%
Adipose
Blood & T−cell
Brain
Digestive
ENCODE
Epithelial
ES cells
ES derived cells
Heart
HSC & B−cell
IMR90
iPSC
Mesenchymal
Muscle
Myosatellite
Neurospheres
Other
Smooth muscle
Thymus
Chromatin state
Active
Inactive

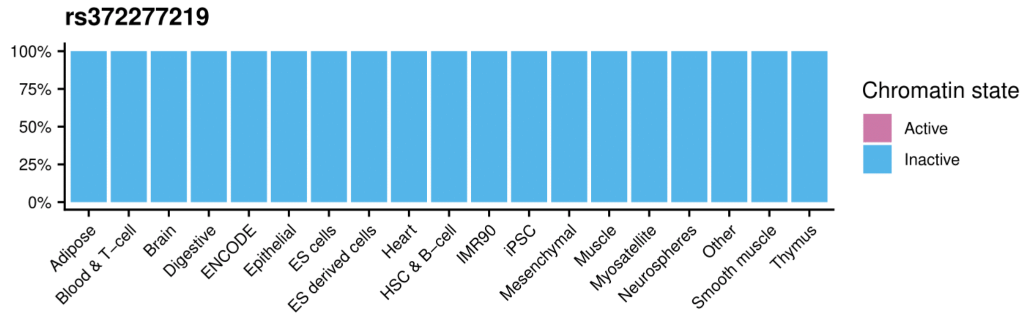
rs372277219
100%
75%
50%
25%
0%
Adipose
Blood & T−cell
Brain
Digestive
ENCODE
Epithelial
ES cells
ES derived cells
Heart
HSC & B−cell
IMR90
iPSC
Mesenchymal
Muscle
Myosatellite
Neurospheres
Other
Smooth muscle
Thymus
Chromatin state
Active
Inactive

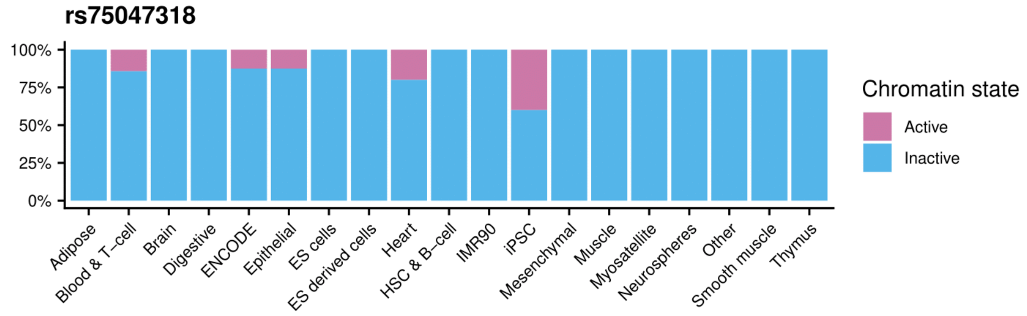
rs75047318
100%
75%
50%
25%
0%
Adipose
Blood & T−cell
Brain
Digestive
ENCODE
Epithelial
ES cells
ES derived cells
Heart
HSC & B−cell
IMR90
iPSC
Mesenchymal
Muscle
Myosatellite
Neurospheres
Other
Smooth muscle
Thymus
Chromatin state
Active
Inactive

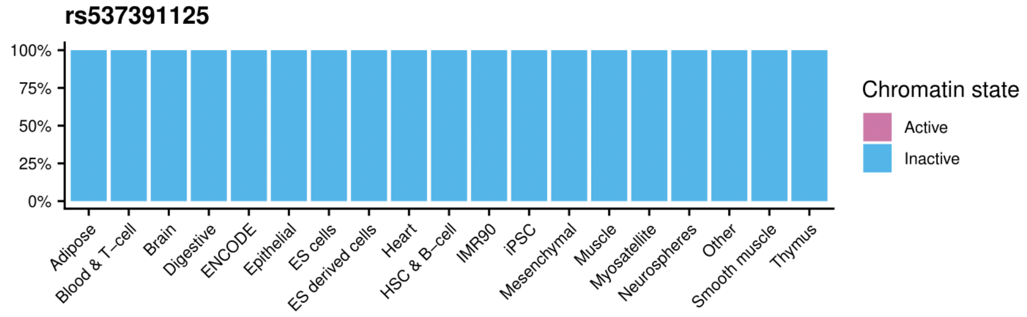
rs537391125
100%
75%
50%
25%
0%
Adipose
Blood & T−cell
Brain
Digestive
ENCODE
Epithelial
ES cells
ES derived cells
Heart
HSC & B−cell
IMR90
iPSC
Mesenchymal
Muscle
Myosatellite
Neurospheres
Other
Smooth muscle
Thymus
Chromatin state
Active
Inactive

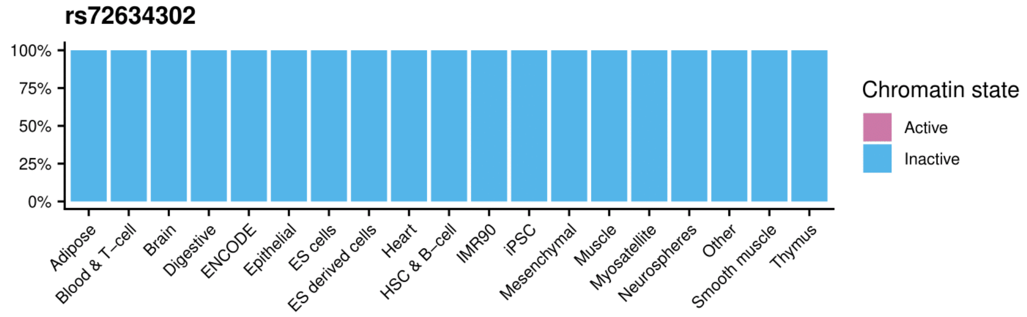
rs72634302
100%
75%
50%
25%
0%
Adipose
Blood & T−cell
Brain
Digestive
ENCODE
Epithelial
ES cells
ES derived cells
Heart
HSC & B−cell
IMR90
iPSC
Mesenchymal
Muscle
Myosatellite
Neurospheres
Other
Smooth muscle
Thymus
Chromatin state
Active
Inactive

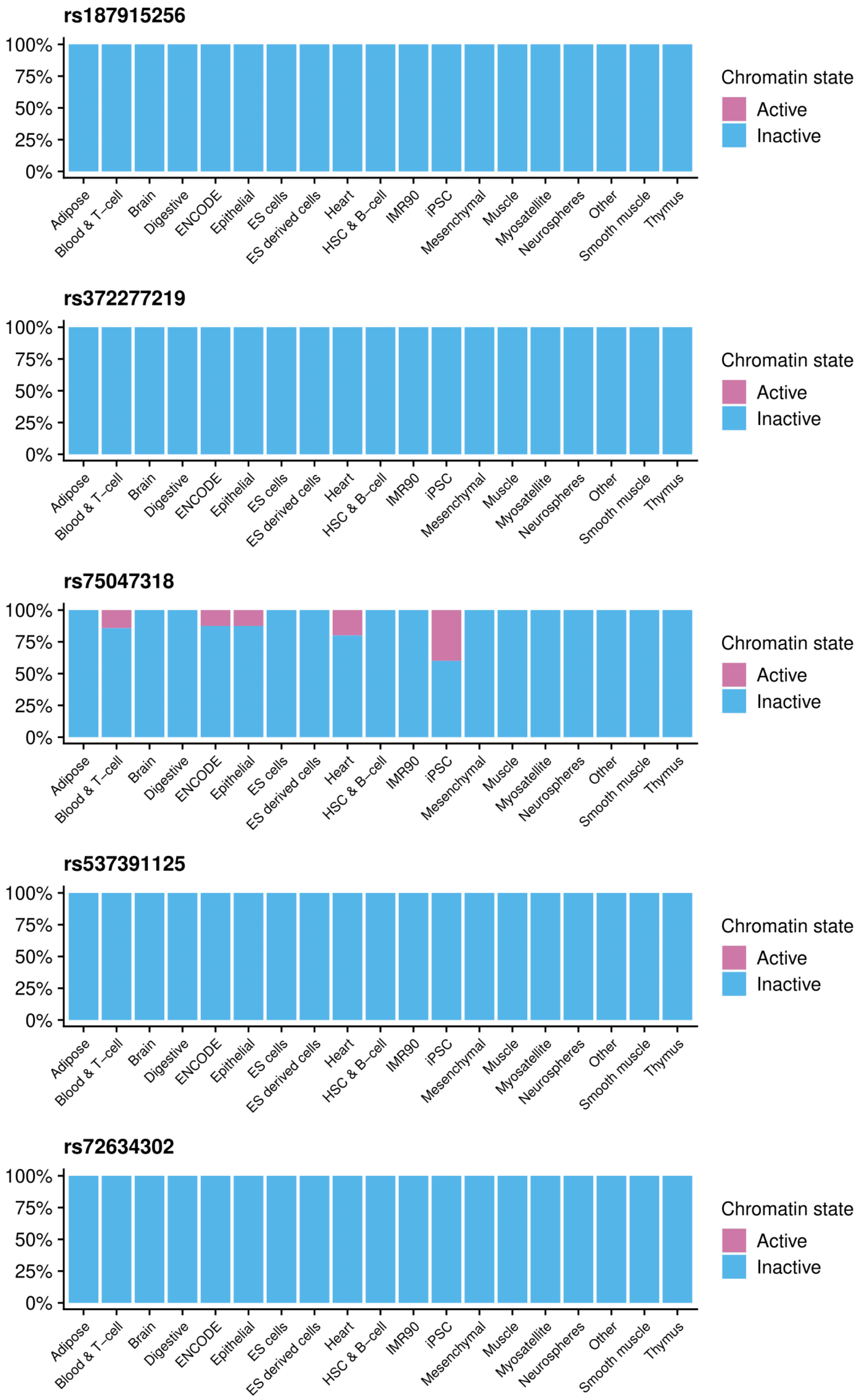
rs187915256
rs372277219
rs75047318
rs537391125
rs72634302
100%
75%
50%
25%
0%
Adipose
Blood & T−cell
Brain
Digestive
ENCODE
Epithelial
ES cells
ES derived cells
Heart
HSC & B−cell
IMR90
iPSC
Mesenchymal
Muscle
Myosatellite
Neurospheres
Other
Smooth muscle
Thymus
Chromatin state
Active
Inactive

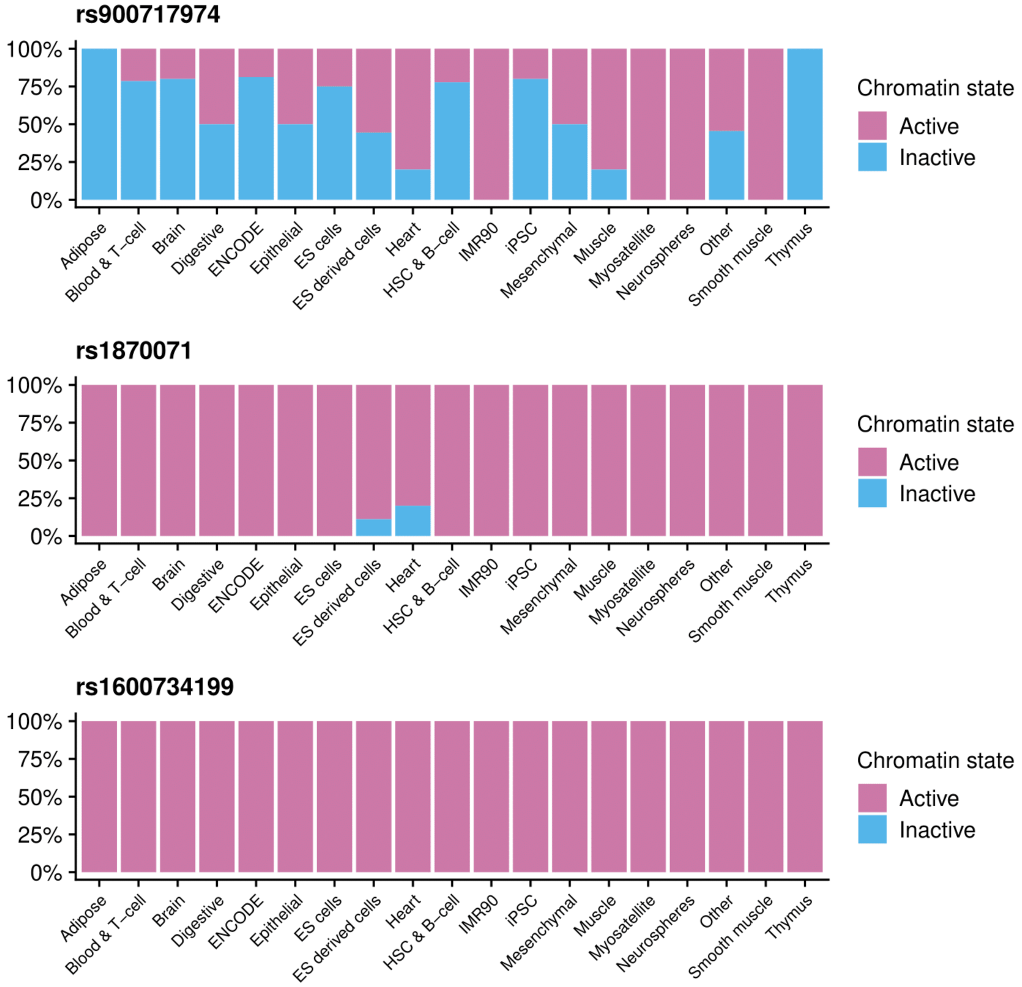

Figure S14: Chromatin state of the candidate SNPs of PNG lowlanders for the epigenomes of the Roadmap project clustered in tissues. Figure generated with the ggplot2 package (v3.4.2).

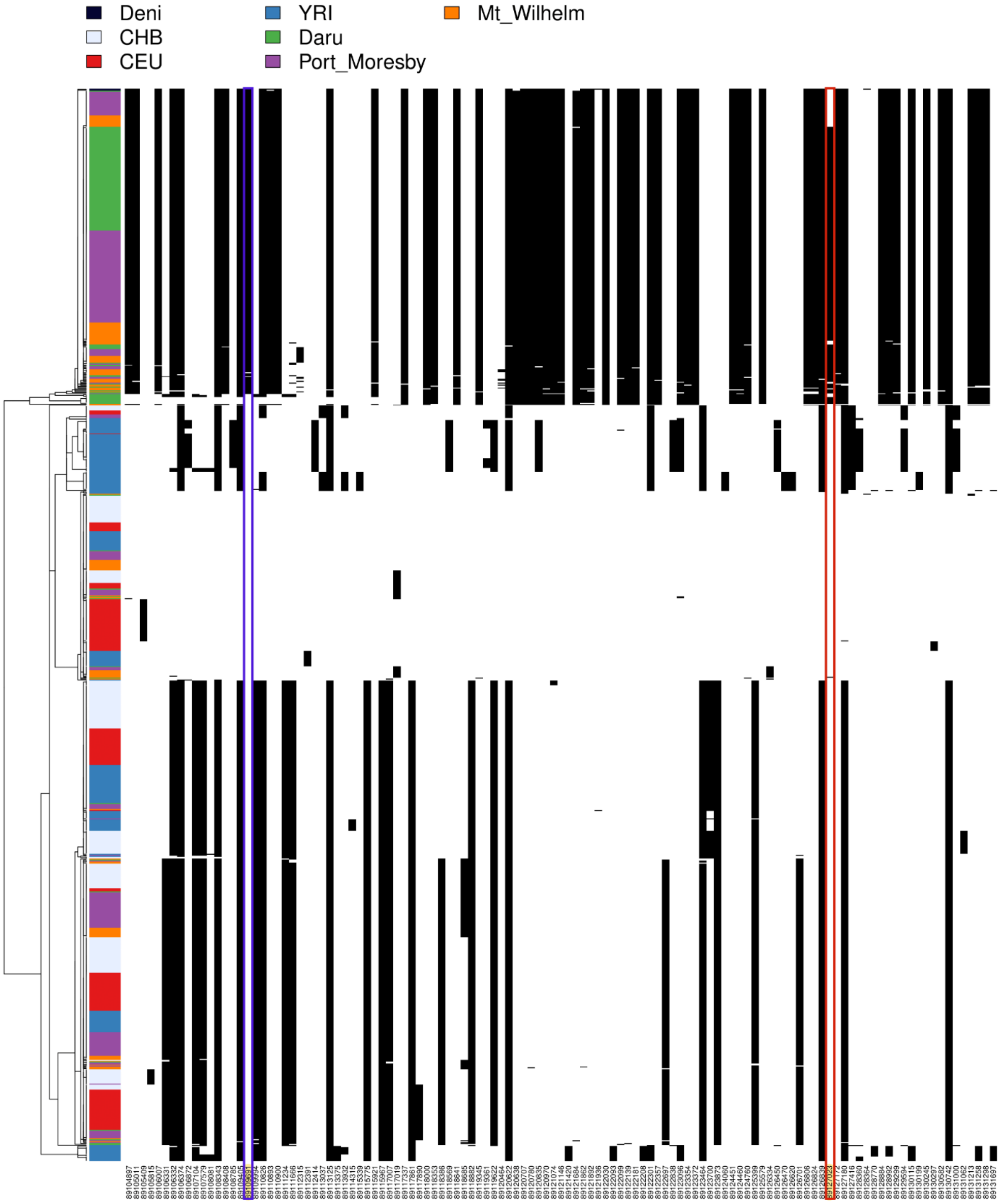

Figure S15: Haplostrips plot for the region chr1:88800562-89326878 overlapping with the GBP locus and under selection in PNG lowlanders. Derived alleles are plotted in black, and ancestral are in white. The introgressed haplotype carries the SNP driving selection for the region (rs368120563-T/C, framed in orange), but the Altai Denisovan does not have this particular allele. On the contrary, the missense variant (framed in blue) in LD with rs36812056 is found in the introgressed haplotype and in the Denisovan genome.

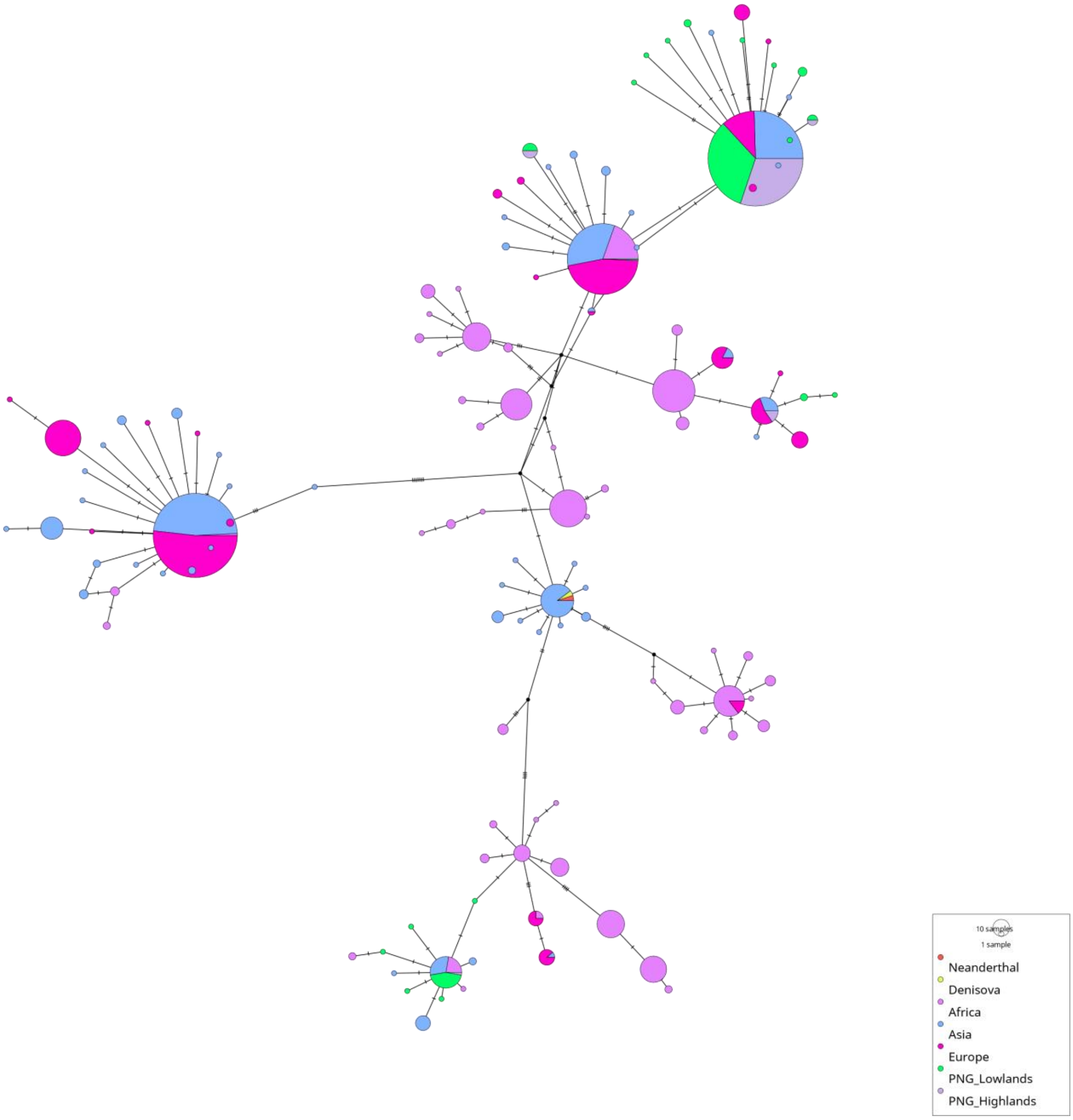

Figure S16: median-joining haplotype networks for the windows 5kbp down- and upstream rs74621527, the candidate SNP for the genomic region chr2:151012094-151201575 under selection in PNG highlanders. This variant is in high-LD (r^2>0.5) with an introgressed ambiguous (Denisovan and Neanderthal) haplotype.

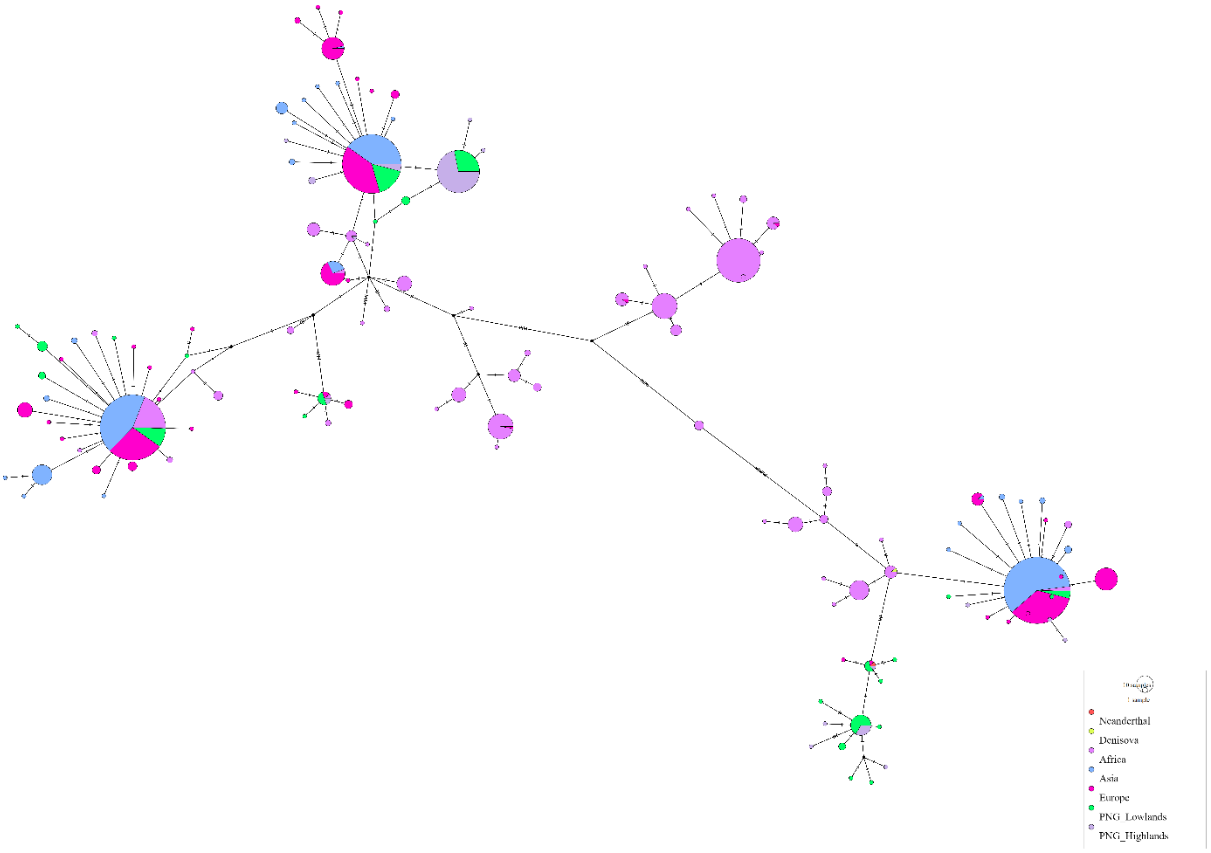

Figure S17: median-joining haplotype networks for the windows 5kbp down- and upstream rs376870800, the candidate SNP for the genomic region chr12:58391529-58634980 under selection in PNG highlanders. This variant is in high-LD (r^2>0.5) with an introgressed ambiguous (Denisovan and Neanderthal) haplotype.

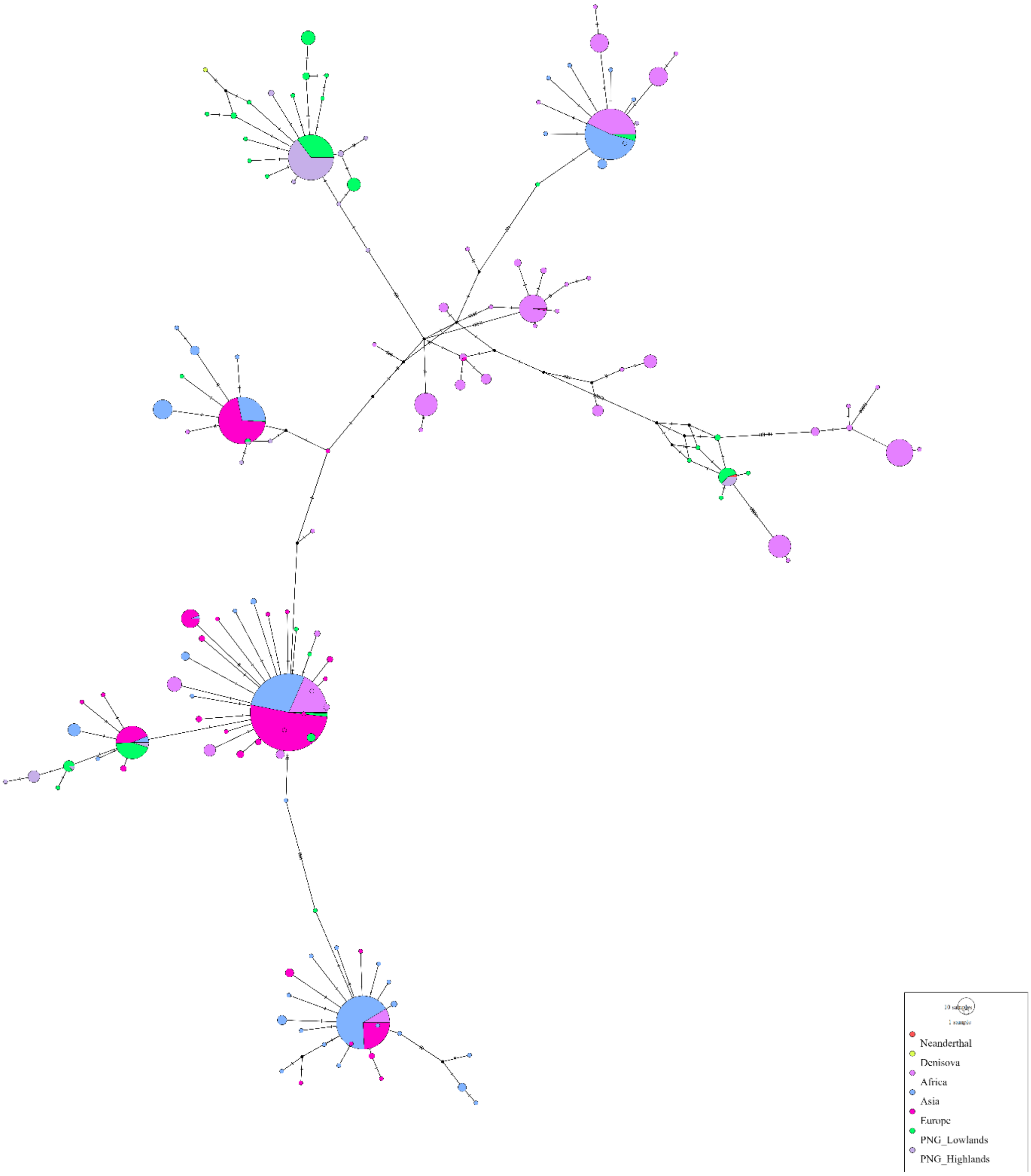

Figure S18: median-joining haplotype networks for the windows 5kbp down- and upstream rs371858795, the candidate SNP for the genomic region chr18:4072997-4251153 under selection in PNG highlanders. This variant is in high-LD ($r^2>0.5$) with an introgressed ambiguous (Denisovan and Neanderthal) haplotype.

Supplementary Tables

Table S1: Sequences from PNG diversity set I, PNG highlanders, and PNG lowlanders datasets. Sequences kept after quality and kinsh

	PNG diversity set I	PNG highlanders	PNG lowlanders	Total
**All sequences**	122	60	80	262
**Variant calling quality control**	0	0	1	1
**misslabelled**	0	4	0	4
**kinship**	1	1	4	6
**Low call rate**	0	1	1	2
**Total Removed**	1	6	6	13
**Total Sequences kept**	121	54	74	249

Table S2: Count of the phenotype measurements for each sampling places

	PNG lowlanders	PNG highlanders	PNG diversity set I	Total
**number_all**	72	52	110	234
**mean age**	45	35	24.3	33
**Number_M**	63	45	53	161
**Number_F**	9	7	57	73
**Height**	72	51	110	233
**Weight**	72	52	110	234
**BMI**	72	51	110	233
**Waist**	72	52	13	137
**Chest_min**	64	33	12	109
**Chest_max**	64	34	12	110
**FVC**	27	16	0	43
**FEV1**	27	16	0	43
**PEF**	27	16	0	43
**Dia**	72	52	24	148
**Sys**	72	52	24	148
**HeartbeatFinal**	72	52	108	232
**Haemoglobine**	56	50	20	126

Table S6: D-statistic analysis of the form D(W,X;Y,Z)

Pop1 (W)	Pop2 (X)	Pop3 (Y)	Pop4 (Z)	D(W,X,Y,Z)	Z	BABA	ABBA
PNG highlanders	PNG lowlanders	CHB	YRI	-0.0002	-0.145	224222	224291
PNG highlanders	PNG diversity I	CHB	YRI	-0.0232	-22.874*	227731	238535
PNG diversity I	PNG lowlanders	CHB	YRI	0.0226	30.339*	242492	231756

* statistically significant admixture (|Z|>3)
D < 0 reflects a excess in ABBA and a gene flow between pop 2 and pop3
D > 0 reflects a excess in BABA and a gene flow between pop 1 and pop3

Table S4: Top regions under selection in PNG highlanders

P-value were computed using a one sided random sampling approach (Skoglund et al., 2017). The detailed approach description can be found in the supplementary note S9.

	CHR	Start (BP,GRCh38)	End (BP,GRCh38)	Score	Z	p-value
**XP-EHH**						
	18	4132370	4199172	6.176	6.211	2.64E-10
	12	58448770	58576778	6.062	6.098	5.37E-10
	12	6502552	6612260	5.090	5.136	1.40E-07
	1	95579290	95686826	5.038	5.085	1.84E-07
	9	85508922	85695092	5.016	5.063	2.06E-07
	4	152754503	152920509	5.007	5.054	2.16E-07
	4	110232324	110334099	4.931	4.979	3.20E-07
	6	30966070	31103184	4.905	4.953	3.66E-07
	3	13118431	13167789	4.636	4.686	1.39E-06
	3	13060340	13100805	4.608	4.658	1.59E-06
**PBS**						
	22	45569818	45594906	1.134	4.872	5.52E-07
	12	58442097	58469168	0.634	3.980	3.45E-05
	3	61894529	61953999	0.602	3.899	4.82E-05
	7	147640904	147668219	0.588	3.864	5.58E-05
	2	151062094	151151575	0.587	3.861	5.66E-05
	10	131162245	131185951	0.585	3.855	5.79E-05
	6	33057609	33082312	0.582	3.848	5.94E-05
	13	47689988	47775193	0.548	3.755	8.66E-05
	12	58482695	58583316	0.546	3.749	8.88E-05
	13	104785633	104825020	0.534	3.715	1.02E-04
**Fisher Score**						
	12	58441529	58584980	8.846	3.096	9.81E-04
	18	4122997	4201153	8.250	3.015	1.28E-03
	12	103833315	104071479	6.713	2.777	2.74E-03
	22	45575411	45590656	6.429	2.727	3.20E-03
	6	33056055	33082312	6.264	2.697	3.50E-03
	12	9936812	10005333	6.251	2.695	3.52E-03
	13	104784734	104816123	6.193	2.684	3.64E-03
	3	61829523	61959858	6.193	2.684	3.64E-03
	14	60207772	60327317	6.124	2.671	3.78E-03
	14	92280479	92351520	6.124	2.671	3.79E-03

Table S5: Top regions under selection in PNG lowlanders

P-value were computed using a one sided random sampling approach (Skoglund et al., 2017).
The detailed approach description can be found in the supplementary note S9.

	CHR	Start (BP,GRCh38)	End (BP,GRCh38)	Score	Z	p-value
**XP-EHH**						
	1	88990863	89202535	-5.968	-5.810	3.12E-09
	2	241871368	241989102	-4.906	-4.759	9.71E-07
	13	61640770	61943327	-4.843	-4.697	1.32E-06
	17	54053406	54172843	-4.710	-4.565	2.49E-06
	7	129604951	129773933	-4.598	-4.454	4.21E-06
	16	87856834	87878392	-4.500	-4.357	6.59E-06
	19	16394294	16526199	-4.435	-4.293	8.81E-06
	14	37187933	37332802	-4.426	-4.284	9.16E-06
	9	93767217	93827803	-4.318	-4.177	1.48E-05
	5	65554470	65658617	-4.288	-4.147	1.68E-05
**PBS**						
	1	89070336	89276878	0.776	4.469	3.93E-06
	8	133841891	133912825	0.540	3.958	3.79E-05
	2	241809136	242016140	0.477	3.782	7.79E-05
	14	77362867	77495559	0.470	3.761	8.45E-05
	1	237877847	237942467	0.433	3.645	1.34E-04
	19	54226104	54280609	0.425	3.619	1.48E-04
	2	124135628	124199405	0.415	3.585	1.68E-04
	19	11758670	12058034	0.400	3.531	2.07E-04
	2	200288798	200382145	0.393	3.508	2.26E-04
	6	85316477	85433888	0.388	3.489	2.43E-04
**Fisher Score**						
	1	88850562	89276878	10.317	3.607	1.55E-04
	2	241866673	242038831	7.383	3.184	7.27E-04
	7	129598370	129786070	6.670	3.056	1.12E-03
	14	77362867	77508267	6.332	2.990	1.40E-03
	18	41183289	41568597	6.315	2.986	1.41E-03
	4	171841098	171936729	6.220	2.967	1.50E-03
	4	82800503	83096792	6.219	2.967	1.50E-03
	12	120403731	120616335	6.137	2.950	1.59E-03
	19	54226104	54280609	6.119	2.947	1.61E-03
	13	89710867	89870623	6.061	2.935	1.67E-03

Table S6: Candidate SNPs driving selection in PNG highlanders

CHR	rs id	XP-EHH SNP	PBS SNP	Daf HL	Daf LL	mean logLR HL 95%CI	mean sel coef HL 95%CI	mean logLR LL 95% CI	mean sel coef LL 95%CI	F_{ST} HL LL	DAF CHB	DAF EUR (UKBB)	distance to the closest SNP in UKBB (GRCh38)	Top association UKBiobank	-LOG(pval)	Beta*	Top assoc pheno gemma	pval gemma	Beta gemma*	variant consequences (VEP)
1	rs887476833	2.458	0.528	0.546	0.189	5.257 [4.319-6.055]	0.006 [0.004-0.008]	1.53 [1.239-1.994]	0.003 [0.003-0.004]	0.242	0.000	NA	33	.	.	.	Height	0.46	-1.26	
2	rs74621527	0.322	0.552	0.917	0.804	2.804 [1.674-4.724]	0.004 [0.003-0.005]	0.809 [0.348-1.257]	0.002 [0.001-0.002]	0.041	0.165	0.098	.	.	.	.	Forced volume capacity	0.51	0.38	
3	rs374181005	1.625	0.223	0.407	0.236	5.889 [4.883-6.829]	0.009 [0.006-0.012]	2.476 [1.931-3.526]	0.004 [0.003-0.005]	0.059	0.000	0.011	50	.	.	.	Height	0.44	1.31	
3	rs79600167	2.939	0.656	0.769	0.412	5.672 [2.95-7.854]	0.004 [0.003-0.006]	2.626 [1.55-3.244]	0.002 [0.002-0.003]	0.222	0.000	NA	.	.	.	.	Forced volume capacity	0.55	-0.21	regulatory region variant
4	rs943845085	3.406	0.528	0.417	0.054	4.584 [3.801-5.192]	0.006 [0.005-0.008]	2.804 [2.183-3.477]	0.02 [0.014-0.026]	0.327	0.000	NA	22	.	.	.	Diastolic blood pressure	0.46	-3.58	
4	rs369030953	1.954	0.580	0.593	0.216	4.185 [3.178-5.223]	0.004 [0.003-0.006]	1.431 [1.265-1.529]	0.002 [0.002-0.003]	0.256	0.000	NA	.	.	.	.	Diastolic blood pressure	0.48	-2.58	
6	rs940110341	4.720	0.610	0.611	0.223	7.149 [6.131-8.264]	0.007 [0.005-0.008]	2.209 [2.003-2.502]	0.004 [0.003-0.004]	0.267	0.000	NA	94	.	.	.	Haemoglobin concentration	0.17	0.89	
6	rs9277772	0.810	0.016	0.213	0.216	3.243 [2.306-4.244]	0.012 [0.008-0.019]	1.719 [1.275-1.956]	0.003 [0.002-0.004]	-0.008	0.257	0.157	.	**Non-cancer illness code, self-reported**	**-124.40**	**0.24**	Diastolic blood pressure	0.67	2.03	regulatory region variant
7	rs17170618	0.245	0.052	0.519	0.514	3.892 [2.951-4.774]	0.005 [0.003-0.007]	5.088 [3.84-6.138]	0.003 [0.003-0.004]	-0.008	0.335	0.191	.	.	.	.	Diastolic blood pressure	0.46	-2.90	
9	rs28728004	3.859	0.475	0.685	0.419	5.119 [4.111-5.92]	0.004 [0.003-0.006]	4.294 [3.751-4.746]	0.004 [0.003-0.004]	0.126	0.015	NA	7	.	.	.	Diastolic blood pressure	0.60	-2.21	
10	rs10829909	-0.755	0.064	0.426	0.338	3.169 [1.879-4.323]	0.005 [0.003-0.007]	0.434 [0.143-0.634]	0.001 [0.001-0.001]	0.008	0.121	0.125	.	.	.	.	Waist circumference	0.14	2.95	
12	rs74576183	4.612	0.480	0.713	0.459	4.142 [2.462-5.901]	0.003 [0.003-0.004]	3.738 [3.443-4.047]	0.003 [0.003-0.003]	0.116	0.117	0.009	.	**Red blood cell (erythrocyte) count**	**-11.74**	**0.05**	Heart rate	0.05	-2.98	
12	rs536947	3.425	0.313	0.907	0.466	4.263 [2.541-6.715]	0.004 [0.003-0.005]	0.152 [0.02-0.389]	0 [0-0.001]	0.347	0.199	0.156	.	.	.	.	Minimal chest depth	0.30	0.61	
12	rs376870800	4.444	0.830	0.704	0.230	6.624 [4.751-7.641]	0.005 [0.004-0.006]	2.264 [1.847-2.663]	0.004 [0.003-0.005]	0.366	0.000	NA	160	.	.	.	Height	0.62	-0.56	
12	rs1032698711	2.643	0.893	0.472	0.243	8.483 [7.41-9.504]	0.012 [0.009-0.015]	5.352 [4.522-5.982]	0.007 [0.006-0.009]	0.103	0.000	NA	22	.	.	.	Diastolic blood pressure	0.60	-2.24	regulatory region variant
13	rs1033760372	0.854	-0.017	0.194	0.250	4.696 [3.346-7.093]	0.006 [0.004-0.010]	4.249 [3.740-4.870]	0.007 [0.006-0.008]	0.001	0.000	NA	34	.	.	.	Diastolic blood pressure	0.18	-5.83	
13	rs16965509	-0.172	0.360	0.500	0.243	4.065 [2.859-5.072]	0.024 [0.016-0.032]	0.869 [0.336-1.566]	0.002 [0.001-0.002]	0.128	0.451	0.052	.	.	.	.	Diastolic blood pressure	0.46	-2.84	
14	rs1033848215	0.669	0.018	0.324	0.345	3.535 [2.592-4.714]	0.008 [0.005-0.013]	3.761 [3.145-4.091]	0.004 [0.003-0.004]	0.021	0.000	NA	2	.	.	.	Height	0.34	-1.44	
14	rs8003454	-0.147	0.088	0.519	0.399	3.258 [1.625-4.459]	0.004 [0.002-0.006]	3.954 [3.564-4.523]	0.003 [0.003-0.004]	0.229	0.184	0.488	.	.	.	.	Height	0.56	-0.82	
18	rs371858795	5.998	0.711	0.769	0.405	6.268 [4.277-7.774]	0.004 [0.003-0.005]	3.876 [3.254-4.441]	0.004 [0.003-0.005]	0.229	0.000	NA	124	.	.	.	Heart rate	0.39	2.10	
22	rs1601558750	-0.570	0.024	0.102	0.081	1.826 [1.044-2.822]	0.014 [0.007-0.027]	1.918 [1.567-2.219]	0.009 [0.006-0.012]	-0.005	0.000	NA	101	.	.	.	Forced volume capacity	0.51	0.48	

Significant association (log(p-val) < -10.47) with PNG highlanders candidate SNPs or with the closest SNP in a 100bp windows (50 bp up and downstream) in the UK Biobank are given in bold

*Effective allele is the derived allele. When closest SNP doesn't exist in our papuan dataset, beta is given as absolute value

** association results given for the closest SNP

Table S7: Candidate SNPs driving selection in PNG lowlanders

CHR	rs id	XP-EHH SNP	PBS SNP	Daf HL	Daf LL	mean logLR HL 95%CI	mean sel coef HL 95%CI	mean logLR LL 95% CI	mean sel coef LL 95%CI	F_{ST} HL LL	DAF CHB	DAF EUR (UKBB)	distance to the closest SNP in UKBB (GRCh38)	Top association UK Biobank	-LOG(pval)	Beta*	Top assoc pheno gemma	pval gemma	Beta gemma*	variant consequences (VEP)
1	rs368120563	-5.482	1.059	0.872	0.380	7.739 [5.018-11.948]	0.004 [0.003-0.005]	5.062 [4.629-5.778]	0.01 [0.007-0.013]	0.420	0.000	NA	123	.	.	.	Height	0.42	1.38	regulatory region variant
1	rs1574154373	-1.508	0.114	0.135	0.019	3.695 [2.285-5.851]	0.009 [0.006-0.014]	0.961 [0.598-1.335]	0.08 [0.055-0.1]	0.075	0.000	NA	36	.	.	.	Maximal chest depth	0.34	0.93	regulatory region variant
2	rs7583123	0.457	-0.148	0.486	0.630	4.806 [2.838-6.068]	0.003 [0.003-0.004]	2.239 [1.141-3.906]	0.003 [0.002-0.004]	0.033	0.184	0.238	.	.	.	.	Heart rate	0.24	-2.44	.
2	chr2:200269472	NA	NA	0.054	0.000	3.182 [1.876-4.107]	0.023 [0.012-0.037]	NA	NA	0.039	0.000	NA	7	.	.	.	Height	0.49	-3.24	.
2	rs376150658	NA	0.068	0.230	0.130	3.261 [2.435-4.116]	0.005 [0.004-0.007]	3.685 [2.816-4.669]	0.02 [0.014-0.025]	0.025	0.000	NA	8	.	.	.	Maximal chest depth	0.61	0.48	.
4	rs4693058	-3.862	0.331	0.757	0.528	6.859 [3.644-9.831]	0.004 [0.003-0.005]	1.433 [0.219-2.517]	0.002 [0.001-0.003]	0.103	0.301	0.579	.	**Lymphocyte percentage**	**-36.61**	**-0.02**	Heart rate	0.05	3.14	.
4	rs926184421	NA	NA	0.081	0.000	2.658 [1.531-4.049]	0.013 [0.007-0.020]	NA	NA	0.063	0.000	NA	14	**Recent restlessness****	**-11.64**	**0.58**	Height	0.66	-1.17	.
5	rs36003688	-1.921	0.021	0.311	0.259	3.121 [2.018-4.122]	0.005 [0.003-0.007]	0.644 [0.322-1.168]	0.003 [0.002-0.006]	-0.002	0.078	0.016	.	.	.	.	Height	0.57	-0.76	.
6	rs989789809	0.049	0.067	0.142	0.056	2.412 [1.865-3.108]	0.007 [0.005-0.011]	1.807 [0.644-3.307]	0.044 [0.011-0.081]	0.031	0.000	NA	13	.	.	.	Heart rate	0.33	3.73	.
7	rs6950082	-3.511	0.241	0.493	0.259	3.235 [1.787-4.571]	0.003 [0.002-0.003]	1.522 [1.259-1.834]	0.004 [0.003-0.005]	0.101	0.155	NA	3	**Platelet count****	**-35.02**	**0.03**	Heart rate	0.33	2.05	.
8	rs187915256	NA	NA	0.986	1.000	5.307 [4.467-6.098]	0.004 [0.003-0.004]	3.637 [2.567-5.14]	0.003 [0.003-0.004]	0.004	1.000	NA	1	.	.	.	Diastolic blood pressure	0.46	18.61	.
9	rs372277219	1.214	NA	0.223	0.000	3.346 [2.654-3.878]	0.006 [0.004-0.007]	NA	NA	0.192	0.000	NA	143	.	.	.	Height	0.15	2.92	.
12	rs75047318	-3.128	-0.058	0.074	0.148	2.174 [1.028-3.776]	0.015 [0.006-0.026]	0.657 [0.172-1.727]	0.004 [0.001-0.008]	0.021	0.039	0.092	.	**SHBG** (sex hormone-binding globulin)	**-41.26**	**-0.04**	Maximal chest depth	0.11	1.20	regulatory region variant
13	rs537391125	-4.732	-0.027	0.939	0.917	6.616 [5.243-7.907]	0.011 [0.008-0.013]	2.202 [1.471-3.269]	0.028 [0.018-0.043]	-0.004	1.000	NA	81	.	.	.	Maximal chest depth	0.11	-1.15	.
13	rs72634302	-2.033	0.331	0.480	0.167	5.727 [4.642-6.55]	0.004 [0.003-0.005]	0.757 [0.552-0.88]	0.003 [0.002-0.004]	0.188	0.209	0.200	.	.	.	.	Forced volume capacity	0.51	-0.28	.
14	rs1594377001	-4.031	-0.044	0.054	0.111	2.698 [1.602-4.102]	0.021 [0.01-0.033]	2.351 [1.899-3.04]	0.015 [0.01-0.022]	0.014	0.000	NA	27	.	.	.	Height	0.66	0.91	5 prime UTR variant, regulatory region variant
14	rs12885954	-0.330	0.096	0.574	0.463	4.141 [2.343-5.846]	0.003 [0.002-0.003]	2.82 [1.653-3.484]	0.004 [0.002-0.005]	0.017	0.602	0.076	.	.	.	.	Peak expiratory flow	0.38	1.05	.
16	rs2287123	-0.691	0.109	0.318	0.139	2.118 [0.709-4.201]	0.002 [0.001-0.004]	0.445 [0.316-0.553]	0.002 [0.002-0.003]	0.076	0.175	0.041	.	**Noninflammatory female genital disorders**	**-12.05**	**-0.14**	Diastolic blood pressure	0.18	-5.31	.
17	rs575590765	-2.250	-0.077	0.115	0.204	3.021 [1.932-4.716]	0.01 [0.006-0.017]	2.134 [1.85-2.54]	0.008 [0.006-0.01]	0.022	0.000	NA	78	.	.	.	Diastolic blood pressure	0.59	-3.24	.
18	rs2848745	-3.279	0.584	0.946	0.778	2.694 [2.007-3.345]	0.003 [0.003-0.004]	0.315 [0.004-0.642]	-0.001 [-0.002-0]	0.114	0.578	0.528	.	.	.	.	Height	0.46	1.56	.
19	rs900717974	-1.205	0.020	0.108	0.065	3.06 [2.255-3.748]	0.011 [0.007-0.014]	1.414 [0.588-2.623]	0.02 [0.009-0.037]	0.003	0.000	NA	32	.	.	.	Diastolic blood pressure	0.46	-6.08	.
19	rs1870071	-2.788	0.250	0.757	0.417	3.506 [1.526-5.688]	0.003 [0.002-0.004]	0.535 [0.009-1.272]	0.001 [0-0.002]	0.210	0.597	0.689	.	**Lymphocyte count**	**-471.30**	**-0.09**	Height	0.34	1.47	regulatory region variant
19	rs1600734199	-0.044	0.041	0.128	0.065	2.827 [1.828-4.332]	0.01 [0.006-0.016]	0.978 [0.433-1.717]	0.012 [0.005-0.023]	0.014	0.000	NA	90	.	.	.	Diastolic blood pressure	0.46	-5.02	regulatory region variant

Significant association (log(p-val) < -10.47) with PNG lowlanders candidate SNPs or with the closest SNP in a 100bp windows (50 bp up and downstream) in the UK Biobank are given in bold

*Effective allele is the derived allele. When closest SNP doesn't exist in our papuan dataset, beta is given as absolute value

** association results given for the closest SNP

Table S8: mean log LR after 50 CLUES run for the 5 top SNPs in the regions under selection in PNG highlanders
Source data are provided as a Source Data file

Region of interest	CHR	BP GRCh38	mean logLR after 50 runs	DAF (PNG highlanders)
**chr1:95529290-95736826**				
	1	**95547804**	5.257	0.546
	1	95579291	3.834	0.676
	1	95567984	3.252	0.556
	1	95650657	2.945	0.657
	1	95643171	2.464	0.324
**chr2:151012094-151201575**				
	2	**151194524**	2.804	0.917
	2	151166392	2.584	0.917
	2	151154513	1.879	0.102
	2	151062188	1.740	0.167
	2	151166811	1.318	0.056
**chr3:13010340-13217789**				
	3	**13035177**	5.889	0.407
	3	13165457	5.274	0.731
	3	13132090	5.224	0.704
	3	13172682	5.138	0.602
	3	13044657	4.515	0.583
**chr3:61779523-62009858**				
	3	**61949161**	5.672	0.769
	3	61946137	5.514	0.778
	3	61819606	4.763	0.120
	3	61852692	3.097	0.852
	3	61846686	2.541	0.852
**chr4:110182324-110384099**				
	4	**110291463**	4.584	0.417
	4	110232325	3.915	0.676
	4	110320193	2.750	0.769
	4	110264462	2.370	0.731
	4	110296751	2.363	0.759
**chr4:152704503-152970509**				
	4	**152954793**	4.185	0.593
	4	152795365	3.876	0.759
	4	152716551	3.534	0.685
	4	152910966	2.055	0.593
	4	152908101	1.767	0.139
**chr6:30916070-31153184**				
	6	**31000120**	7.149	0.611
	6	31035407	4.267	0.519
	6	30983781	4.189	0.630
	6	30985432	4.065	0.611
	6	31073503	3.192	0.565
**chr6:33006055-33132312**				
	6	**33131649**	3.243	0.213
	6	33093558	3.159	0.306
	6	33128978	2.568	0.639
	6	33104062	2.226	0.315
	6	33107186	1.963	0.417
**chr7:147590904-147718219**				
	7	**147610112**	3.892	0.519
	7	147683897	3.026	0.241
	7	147662601	2.998	0.639
	7	147612922	2.450	0.509
	7	147606714	1.952	0.231
**chr9:85458922-85745092**				
	9	**85560644**	5.119	0.685
	9	85479140	4.466	0.824
	9	85688522	3.359	0.120
	9	85636728	3.301	0.694
	9	85640367	3.208	0.685
**chr10:131112245-131235951**				
	10	**131130644**	3.169	0.426
	10	131131893	3.050	0.426
	10	131140940	2.049	0.380
	10	131160615	1.862	0.491
	10	131171199	1.837	0.954

Region of interest	CHR	BP GRCh38	mean logLR after 50 runs	DAF (PNG highlanders)
**chr12:6452552-6662260**				
	12	**6505015**	4.142	0.713
	12	6516544	3.465	0.056
	12	6479999	2.894	0.296
	12	6648538	2.845	0.713
	12	6502789	2.479	0.722
**chr12:9886812-10055333**				
	12	**9979340**	4.263	0.907
	12	10010984	3.641	0.824
	12	9983698	3.096	0.917
	12	10023006	2.686	0.954
	12	9977095	2.443	0.907
**chr12:58391529-58634980**				
	12	**58569336**	6.624	0.704
	12	58451248	4.934	0.815
	12	58582254	3.961	0.157
	12	58541863	3.443	0.824
	12	58561387	2.135	0.806
**chr12:103783315-104121479**				
	12	**103794720**	8.483	0.472
	12	103989218	6.641	0.731
	12	103903509	6.640	0.704
	12	103914824	6.576	0.722
	12	103827796	6.544	0.620
**chr13:47639988-47825193**				
	13	**47662268**	4.065	0.194
	13	47825176	3.365	0.148
	13	47703856	3.345	0.370
	13	47803405	2.863	0.574
	13	47753020	2.076	0.565
**chr13:104734734-104875020**				
	13	**104870821**	4.696	0.500
	13	104768188	4.250	0.519
	13	104786510	2.400	0.611
	13	104824094	2.379	0.620
	13	104787393	2.236	0.657
**chr14:60157772-60377317**				
	14	**60160438**	3.535	0.324
	14	60158288	2.349	0.139
	14	60352132	2.027	0.398
	14	60354491	2.018	0.398
	14	60331291	1.871	0.731
**chr14:92230479-92401520**				
	14	**92251093**	3.258	0.519
	14	92248897	3.119	0.065
	14	92273678	2.643	0.435
	14	92389176	2.629	0.741
	14	92239129	1.629	0.130
**chr18:4072997-4251153**				
	18	**4162760**	6.268	0.769
	18	4181004	5.063	0.759
	18	4180103	4.955	0.741
	18	4088952	3.812	0.352
	18	4152970	3.450	0.120
**chr22:45519818-45644906**				
	22	**45617645**	1.826	0.102
	22	45536399	1.752	0.306
	22	45637445	1.651	0.148
	22	45634845	1.452	0.056
	22	45610910	1.111	0.315

The candidate SNPs we considered the most likely to drive selection and that we will consider for the association analysis are in bold

Table S9: mean log LR after 50 CLUES run for the 5 top SNPs in the regions under selection in PNG lowlanders

Source data are provided as a Source Data file

Region of interest	CHR	BP GRCh38	mean logLR	DAF (PNG
**chr1:88800562-89326878**				
	**1**	**89127023**	7.739	0.872
	**1**	**89175641**	7.106	0.561
	**1**	**89040215**	4.856	0.291
	**1**	**88915275**	4.215	0.818
	**1**	**89109691**	1.671	0.824
**chr1:237827847-237992467**				
	1	**237859017**	3.695	0.135
	1	237843735	3.476	0.304
	1	237967252	2.440	0.155
	1	237865965	1.997	0.358
	1	237872490	1.743	0.149
**chr2:124085628-124249405**				
	2	**124228148**	4.806	0.486
	2	124117978	4.013	0.561
	2	124233560	3.011	0.432
	2	124232181	2.988	0.426
	2	124206789	2.227	0.061
**chr2:200238798-200432145**	2	**200269472**	3.182	0.054
	2	200300024	3.039	0.980
	2	200383471	2.483	0.054
	2	200321849	2.315	0.865
	2	200325905	2.161	0.331
**chr2:241759136-242088831**				
	2	**241862387**	3.261	0.230
	2	241778517	2.284	0.291
	2	241815140	1.546	0.101
	2	241764015	1.519	0.615
	2	241822219	1.162	0.216
**chr4:171791098-171986729**				
	4	**171895323**	2.658	0.081
	4	171841157	2.316	0.061
	4	171911456	2.231	0.095
	4	171950025	2.098	0.135
	4	171967368	1.663	0.149
**chr4:82750503-83146792**				
	4	**82856713**	6.859	0.757
	4	83036465	2.414	0.824
	4	82816546	2.160	0.074
	4	83116141	1.806	0.419
	4	82861781	1.258	0.054
**chr5:65504470-65708617**				
	5	**65534488**	3.121	0.311
	5	65540191	3.018	0.054
	5	65619024	2.718	0.088
	5	65682263	2.036	0.068
	5	65560665	1.769	0.338
**chr6:85266477-85483888**				
	6	**85351595**	2.412	0.142
	6	85328394	2.254	0.074
	6	85273167	1.682	0.250
	6	85277286	1.615	0.581
	6	85361286	1.209	0.236
**chr7:129548370-129836070**				
	7	**129637870**	3.235	0.493
	7	129637875	2.650	0.162
	7	129673855	2.586	0.777
	7	129672730	1.985	0.818
	7	129754564	1.698	0.108
**chr8:133791891-133962825**				
	8	**133959118**	5.307	0.986
	8	133793941	2.436	0.588
	8	133826446	1.123	0.581
	8	133839210	1.013	0.554
	8	133805132	0.601	0.054
**chr9:93717217-93877803**				
	9	**93845307**	3.346	0.223
	9	93852030	3.241	0.682
	9	93782635	3.086	0.723
	9	93847325	2.823	0.682
	9	93853581	2.697	0.696

Region of interest	CHR	BP GRCh38	mean logLR	DAF (PNG
**chr12:120353731-120666335**				
	**12**	**120431276**	2.174	0.074
	**12**	**120639163**	1.951	0.831
	**12**	**120638240**	1.579	0.838
	**12**	**120639705**	1.527	0.831
	**12**	**120611132**	1.396	0.122
**chr13:61590770-61993327**				
	13	**61791792**	6.616	0.939
	13	61947329	4.363	0.480
	13	61937190	4.356	0.486
	13	61727719	4.037	0.101
	13	61692897	3.685	0.095
**chr13:89660867-89920623**				
	13	**89848283**	5.727	0.480
	13	89858302	4.250	0.541
	13	89851426	3.440	0.520
	13	89712047	2.779	0.736
	13	89661705	2.222	0.088
**chr14:37137933-37382802**				
	14	**37197894**	2.698	0.054
	14	37159526	2.343	0.061
	14	37371863	1.910	0.054
	14	37299777	1.682	0.122
	14	37293216	0.885	0.061
**chr14:77312867-77558267**				
	14	**77524491**	4.141	0.574
	14	77495559	3.054	0.250
	14	77508975	2.825	0.149
	14	77520582	2.496	0.155
	14	77366205	1.772	0.135
**chr16:87806834-87928392**				
	16	**87839137**	2.118	0.318
	16	87811366	2.088	0.182
	16	87855597	1.603	0.399
	16	87824639	1.455	0.520
	16	87869733	0.873	0.223
**chr17:54003406-54222843**				
	17	**54041157**	3.021	0.115
	17	54097704	2.696	0.284
	17	54062441	2.539	0.912
	17	54138655	2.452	0.284
	17	54073758	2.168	0.061
**chr18:41133289-41618597**				
	18	**41477761**	2.694	0.946
	18	41319199	2.283	0.189
	18	41416329	1.938	0.223
	18	41580340	1.913	0.703
	18	41361038	1.669	0.196
**chr19:11708670-12108034**				
	19	**12067828**	3.060	0.108
	19	11759746	2.945	0.959
	19	11989871	2.536	0.061
	19	11993862	2.503	0.108
	19	12027280	2.436	0.081
**chr19:16344294-16576199**				
	19	**16394295**	3.506	0.757
	19	16385981	3.287	0.757
	19	16361060	3.256	0.743
	19	16555290	2.052	0.791
	19	16401701	2.046	0.142
**chr19:54176104-54330609**				
	19	**54200712**	2.827	0.128
	19	54204288	1.591	0.872
	19	54189013	1.591	0.628
	19	54283564	1.545	0.331
	19	54196804	0.682	0.149

The candidate SNPs we considered the most likely to drive selection and that we will consider for the association analysis are in bold

Table S10: Significant association (log(p-val) < -10.47) with PNG highlanders candidate SNPs in the UK Biobank

P-values and betas have been extracted from UK Biobank summary statistics (Pan-UKB team. https://pan.ukbb.broadinstitute.org (2020)).

	Phenotype	Phenotype description	-LOG(p_val)	beta*
**rs9277772**	**categorical-20002-both_sexes-1226**	**Non-cancer illness code, self-reported: hypothyroidism/myxoedema**	**-124.4**	**0.2369**
	categorical-20003-both_sexes-1141191044	Treatment/medication code: levothyroxine sodium	-70.34	0.1882
	phecode-244.4-both_sexes	Hypothyroidism NOS	-62.88	0.1755
	icd10-E03-both_sexes	E03 Other hypothyroidism	-62.28	0.1741
	phecode-244-both_sexes	Hypothyroidism	-59.9	0.1669
	continuous-50-both_sexes-irnt	Standing height	-50.05	0.02655
	prescriptions-levothyroxine-both_sexes	Prescriptions levothyroxine	-34.49	0.1412
	prescriptions-thyroid_hormone-both_sexes	Prescriptions thyroid hormone	-34.49	0.1412
	continuous-20015-both_sexes-irnt	Sitting height	-34.24	0.02357
	continuous-23129-both_sexes-irnt	Trunk fat-free mass	-28.3	0.01658
	continuous-23130-both_sexes-irnt	Trunk predicted mass	-27.9	0.01625
	continuous-51-both_sexes-irnt	Seated height	-27.73	0.01998
	continuous-23125-both_sexes-irnt	Arm fat-free mass (left)	-26.44	0.01582
	continuous-23101-both_sexes-irnt	Whole body fat-free mass	-26.35	0.01586
	continuous-23126-both_sexes-irnt	Arm predicted mass (left)	-26.26	0.01572
	continuous-23102-both_sexes-irnt	Whole body water mass	-24.88	0.0153
	continuous-23105-both_sexes-irnt	Basal metabolic rate	-24.54	0.01597
	continuous-1697-both_sexes	Comparative height size at age 10	-23.62	0.01612
	continuous-23121-both_sexes-irnt	Arm fat-free mass (right)	-23.47	0.01466
	continuous-23122-both_sexes-irnt	Arm predicted mass (right)	-22.29	0.01412
	continuous-23114-both_sexes-irnt	Leg predicted mass (right)	-18.94	0.01347
	continuous-30150-both_sexes-irnt	**Eosinophill count**	-18.76	0.02042
	continuous-23113-both_sexes-irnt	Leg fat-free mass (right)	-18.65	0.01338
	continuous-23117-both_sexes-irnt	Leg fat-free mass (left)	-17.96	0.01337
	categorical-6159-both_sexes-7	Pain type(s) experienced in last month: knee pain	-17.46	0.04276
	continuous-23118-both_sexes-irnt	Leg predicted mass (left)	-16.83	0.0126
	continuous-21002-both_sexes-irnt	Weight	-16.79	0.01715
	continuous-49-both_sexes-irnt	Hip circumference	-16.59	0.01912
	continuous-23098-both_sexes-irnt	Weight	-15.64	0.01647
	biomarkers-30720-both_sexes-irnt	Cystatin C	-15.56	-0.01763
	continuous-30210-both_sexes-irnt	**Eosinophill percentage**	-15.28	0.01827
	continuous-NAP-both_sexes-irnt	Non-albumin protein	-15.25	-0.01867
	continuous-102-both_sexes-irnt	Pulse rate, automated reading	-15.03	-0.01787
	continuous-30100-both_sexes-irnt	**Mean platelet (thrombocyte) volume**	-14.98	0.01979
	continuous-30070-both_sexes-irnt	**Red blood cell (erythrocyte) distribution width**	-14.07	0.01722
	phecode-495-both_sexes	Asthma	-13.18	0.0578
	categorical-20107-both_sexes-3	Illnesses of father: lung cancer	-12.71	0.05301
	continuous-30010-both_sexes-irnt	**Red blood cell (erythrocyte) count**	-12.71	-0.01399
	icd10-J45-both_sexes	J45 Asthma	-12.71	0.05623
	categorical-2492-both_sexes-2492	Taking other prescription medications	-12.26	0.02967
	biomarkers-30860-both_sexes-irnt	Total protein	-12.18	-0.01616
	continuous-1737-both_sexes	Childhood sunburn occasions	-11.87	-0.015
	continuous-20153-both_sexes-irnt	Forced expiratory volume in 1-second (FEV1), predicted	-11.39	0.009274
	categorical-20107-both_sexes-101	Illnesses of father	-11.37	-0.03292
	continuous-30090-both_sexes-irnt	**Platelet crit**	-11.3	0.0155
	biomarkers-30630-both_sexes-irnt	Apolipoprotein A	-11	0.01497
	continuous-AG-both_sexes-irnt	Albumin/Globulin ratio	-10.74	0.0152
	biomarkers-30760-both_sexes-irnt	HDL cholesterol	-10.7	0.01497
**rs74576183**	continuous-30010-both_sexes-irnt	**Red blood cell (erythrocyte) count**	-11.74	0.04942
	continuous-30020-both_sexes-irnt	**Haemoglobin concentration**	-10.97	0.04307

*effective allele is the derived allele that is under positive selection

Phenotypes from the "Blood count" category 100081 of the UKBB are shown in bold

Table S11: Significant association (log(p-val) < -10.47) with PNG lowlanders candidate SNPs in the UK Biobank

P-values and betas have been extracted from UK Biobank summary statistics (Pan-UKB team. https://pan.ukbb.broadinstitute.org (2020)).

	Phenotype	Phenotype description	-LOG(p_val)	beta*
**rs4693058**				
	continuous-30180-both_sexes-irnt	**Lymphocyte percentage**	-36.61	-0.02079
	continuous-30200-both_sexes-irnt	**Neutrophil percentage**	-29.54	0.01871
	continuous-30120-both_sexes-irnt	**Lymphocyte count**	-27.08	-0.01837
	continuous-30070-both_sexes-irnt	**Red blood cell (erythrocyte) distribution width**	-20.23	0.0155
	biomarkers-30630-both_sexes-irnt	Apolipoprotein A	-11.76	0.01146
	biomarkers-30760-both_sexes-irnt	HDL cholesterol	-11.13	0.01129
**rs75047318**	biomarkers-30830-both_sexes-irnt	SHBG	-41.26	-0.03747
	continuous-50-both_sexes-irnt	Standing height	-19.15	-0.02032
	continuous-20151-both_sexes-irnt	Forced vital capacity (FVC), Best measure	-17.95	-0.01706
	continuous-LDLC-both_sexes-medadj_irnt	LDL direct, adjusted by medication	-14.97	0.02284
	continuous-3062-both_sexes-irnt	Forced vital capacity (FVC)	-14.79	-0.01414
	continuous-eGFR-both_sexes-irnt	Estimated glomerular filtration rate, serum creatinine	-14.79	0.01923
	biomarkers-30610-both_sexes-irnt	Alkaline phosphatase	-13.59	0.02217
	continuous-1180-both_sexes	Morning/evening person (chronotype)	-13.42	0.01989
	continuous-LDLC-both_sexes-medadj_raw	LDL direct, adjusted by medication	-13.04	0.01835
	continuous-20150-both_sexes-irnt	Forced expiratory volume in 1-second (FEV1), Best measure	-12.6	-0.01444
	biomarkers-30700-both_sexes-irnt	Creatinine	-11.3	-0.01583
	biomarkers-30870-both_sexes-irnt	Triglycerides	-11.13	0.01903
	biomarkers-30680-both_sexes-irnt	Calcium	-10.96	0.01906
	continuous-30020-both_sexes-irnt	**Haemoglobin concentration**	-10.51	0.01451
**rs2287123**	phecode-619-females	Noninflammatory female genital disorders	-12.05	-0.1362
**rs1870071**	continuous-30120-both_sexes-irnt	**Lymphocyte_count**	-471.3	-0.08507
	continuous-30180-both_sexes-irnt	**Lymphocyte_percentage**	-280.3	-0.06315
	continuous-30200-both_sexes-irnt	**Neutrophill_percentage**	-174.1	0.05
	continuous-30000-both_sexes-irnt	**White_blood_cell_(leukocyte)_count**	-103.5	-0.03304
	continuous-NAP-both_sexes-irnt	Non-albumin_protein	-43.19	-0.02571
	continuous-AG-both_sexes-irnt	Albumin/Globulin_ratio	-34.46	0.02262
	continuous-30190-both_sexes-irnt	**Monocyte_percentage**	-33.77	0.02164
	biomarkers-30860-both_sexes-irnt	Total_protein	-30.31	-0.02093
	categorical-6149-both_sexes-1: mouth ulcers	Mouth/teeth_dental_problems	-16.31	-0.04353
	continuous-30160-both_sexes	**Basophill_count**	-13.63	-0.009662
	continuous-30040-both_sexes-irnt	**Mean_corpuscular_volume**	-12.84	-0.0131
	categorical-6152-both_sexes-100	Blood_clot,_DVT,_bronchitis,_emphysema,_asthma,_rhinitis,_eczema,_allergy_diagnosed_by_doctor	-12.13	-0.02543
	prescriptions-atorvastatin-both_sexes	prescription atorvastatin	-10.58	-0.04032

*effective allele is the derived allele that is under positive selection

Phenotypes from the "Blood count" category 100081 of the UKBB are shown in bold

Table S12: Significant association (log(p-val) < -10.47) with the closest SNP to PNG lowlanders candidate SNPs (50 bp upstream and downstream) in the UK Biobank

P-values and betas have been extracted from UK Biobank summary statistics (Pan-UKB team. https://pan.ukbb.broadinstitute.org (2020)).

	Phenotype	Phenotype description	-LOG(p_val)	beta
**rs116353753** (closest SNP to rs926184421)				
	continuous-20516-both_sexes	Recent restlessness	-11.64	0.583
**rs6968168** (closest SNP to rs6950082)				
	continuous-30080-both_sexes-irnt	**Platelet count**	-35.02	0.03
	continuous-30090-both_sexes-irnt	**Platelet crit**	-23.32	0.02
	continuous-30100-both_sexes-irnt	**Mean platelet (thrombocyte) volume**	-14.17	0.02
	continuous-23108-both_sexes-irnt	Impedance of leg (left)	-11.73	0.01
	continuous-23107-both_sexes-irnt	Impedance of leg (right)	-11.25	0.01

*beta is given as the absolute value

Phenotypes from the "Blood count" category 100081 of the UKBB are shown in bold

Table S16: Gemma results for the 44 candidate SNPs. P-val is adjusted for multitesting using Benjamini-Hochberg procedure for the number of SNPs tested

P-values and Betas obtained from GEMMA LMM for the SNPs of interest and the phenotype residuals, corrected for the relatedness matrix. Betas are given between parenthesis.

For each phenotype, given P-values are corrected for multi-testing across 44 SNPs with the Benjamini-Hochberg procedure (Benjamini & Hochberg, 1995).

For further discussion on the multi-testing burden see Supplementary Note S15

CHR	Candidate SNP	pos (BP, GRCh38)	Waist**	FEV1**	Chest min**	Hemoglobine*	Weight**	Heart rate*	Dia*	Height*	FVC**	PEF**	Sys*	Chest max**	BMI*
chr1	rs368120563	89127023	0.967 (0.728)	0.67 (-0.318)	0.969 (0.019)	0.935 (-0.246)	0.998 (-0.475)	0.863 (-0.269)	0.799 (1.022)	0.416 (1.378)	0.505 (-0.284)	0.995 (-0.421)	0.867 (1.127)	0.96 (0.014)	0.999 (-0.172)
chr1	rs887476833	95547804	0.967 (1.137)	0.681 (0.155)	0.765 (0.306)	0.935 (0.142)	0.998 (0.907)	0.821 (0.662)	0.869 (-0.476)	0.463 (-1.264)	0.505 (0.286)	0.995 (0.05)	0.959 (0.374)	0.677 (0.38)	0.999 (0.394)
chr1	.	237859017	0.967 (1.737)	0.681 (-0.249)	0.628 (0.768)	0.865 (-0.743)	0.998 (0.373)	0.821 (-1.488)	0.848 (1.318)	0.536 (2.02)	0.505 (-0.479)	0.995 (-0.1)	0.867 (-1.825)	0.336 (0.926)	0.999 (0.057)
chr2	rs7583123	124228148	0.967 (-0.259)	0.67 (0.216)	0.882 (-0.086)	0.56 (0.515)	0.998 (-0.352)	0.239 (-2.436)	0.848 (0.76)	0.463 (-1.07)	0.741 (0.15)	0.995 (0.241)	0.867 (1.675)	0.949 (0.055)	0.999 (-0.176)
chr2	rs74621527	151194524	0.967 (-0.46)	0.67 (0.319)	0.882 (-0.098)	0.865 (-0.553)	0.998 (-0.439)	0.821 (-1.129)	0.7 (-2.162)	0.622 (0.724)	0.505 (0.383)	0.995 (0.432)	0.867 (4.449)	0.949 (-0.057)	0.999 (-0.026)
chr2	.	200269472	0.967 (-2.464)	0.67 (-0.67)	0.87 (-0.545)	0.935 (0.843)	0.949 (-6.645)	0.857 (-1.179)	0.869 (1.455)	0.487 (-3.238)	0.505 (-0.628)	0.83 (-1.975)	0.867 (4.34)	0.884 (-0.55)	0.727 (-2.372)
chr2	rs376150658	241862387	0.967 (0.906)	0.989 (-0.018)	0.765 (0.305)	0.971 (-0.086)	0.998 (1.339)	0.821 (-1.154)	0.848 (-0.916)	0.665 (-0.55)	0.921 (-0.048)	0.995 (-0.299)	0.867 (2.831)	0.613 (0.477)	0.999 (0.465)
chr3	rs374181005	13035177	0.967 (-1.01)	0.929 (-0.067)	0.765 (0.376)	0.935 (0.259)	0.998 (-0.429)	0.821 (1.275)	0.848 (-0.626)	0.439 (1.309)	0.741 (-0.104)	0.995 (-0.159)	0.867 (5.216)	0.613 (0.401)	0.999 (-0.113)
chr3	rs79600167	61949161	0.967 (-0.284)	0.67 (-0.192)	0.87 (0.192)	0.935 (-0.17)	0.998 (-0.314)	0.976 (-0.03)	0.785 (-1.191)	0.567 (-0.753)	0.55 (-0.21)	0.995 (-0.365)	0.867 (-2.364)	0.949 (0.075)	0.999 (-0.061)
chr4	rs4693058	82856713	0.967 (0.444)	0.963 (0.049)	0.882 (-0.148)	0.971 (-0.095)	0.998 (-0.62)	**0.046 (3.137)**	0.481 (2.675)	0.562 (0.804)	0.921 (-0.033)	0.995 (-0.256)	0.867 (2.896)	0.949 (-0.077)	0.999 (-0.214)
chr4	rs943845085	110291463	0.967 (-0.988)	1.0 (0.003)	0.882 (0.221)	0.935 (0.224)	0.949 (2.684)	0.821 (1.807)	0.461 (-3.583)	0.562 (-1.135)	0.741 (0.14)	0.995 (-0.389)	0.867 (-2.358)	0.914 (0.196)	0.727 (0.999)
chr4	rs369030953	152954793	0.981 (0.085)	1.0 (0.002)	0.765 (0.31)	0.935 (0.282)	0.998 (1.432)	0.821 (0.538)	0.481 (-2.578)	0.935 (-0.092)	0.921 (0.03)	0.995 (-0.478)	0.959 (0.376)	0.744 (0.306)	0.727 (0.671)
chr4	rs926184421	171895323	0.967 (-1.273)	0.71 (-0.538)	0.882 (-0.396)	0.865 (-1.131)	0.998 (-0.24)	0.821 (-1.214)	0.946 (-0.274)	0.657 (-1.174)	0.853 (-0.338)	0.995 (-2.024)	0.867 (-3.603)	0.949 (-0.158)	0.999 (-0.027)
chr5	rs36003688	65534488	0.967 (0.483)	0.681 (0.166)	0.997 (0.001)	0.971 (-0.029)	0.998 (-0.569)	0.821 (-1.027)	0.869 (0.462)	0.567 (-0.761)	0.741 (0.127)	0.995 (0.212)	0.867 (2.635)	0.949 (0.064)	0.999 (-0.156)
chr6	rs940110341	31000120	0.967 (-1.384)	0.829 (0.106)	0.882 (0.093)	0.171 (0.886)	0.998 (0.404)	0.821 (0.522)	0.785 (-1.149)	0.622 (-0.606)	0.741 (0.152)	0.995 (-0.076)	0.867 (1.023)	0.949 (0.065)	0.999 (0.122)
chr6	rs9277772	33131649	0.967 (1.162)	1.0 (0.0)	0.765 (0.37)	0.989 (0.008)	0.998 (0.6)	0.858 (0.342)	0.672 (2.029)	0.928 (-0.125)	0.921 (0.045)	0.995 (0.251)	0.867 (-1.055)	0.719 (0.332)	0.999 (0.242)
chr6	rs989789809	85351595	0.967 (0.835)	0.978 (0.037)	0.882 (-0.135)	0.971 (0.087)	0.998 (0.09)	0.333 (3.728)	0.848 (0.94)	0.463 (-1.864)	0.741 (-0.207)	0.995 (0.086)	0.959 (0.461)	0.949 (-0.039)	0.999 (-0.017)
chr7	rs6950082	129637870	0.979 (0.105)	0.67 (-0.213)	0.765 (-0.255)	0.875 (-0.329)	0.998 (-1.031)	0.333 (2.049)	0.6 (-2.094)	0.622 (0.576)	0.505 (-0.305)	0.995 (-0.036)	0.867 (-2.669)	0.719 (-0.287)	0.999 (-0.376)
chr7	rs17170618	147610112	0.967 (-0.907)	0.978 (0.024)	0.894 (-0.057)	0.935 (-0.143)	0.998 (0.009)	0.821 (0.979)	0.461 (-2.899)	0.562 (-0.821)	0.934 (0.013)	0.995 (0.084)	0.867 (1.143)	0.884 (-0.178)	0.999 (-0.056)
chr8	rs187915256	133959118	0.967 (-6.704)	NA	0.882 (-0.696)	0.935 (-1.093)	0.998 (-1.211)	0.821 (3.61)	0.461 (18.607)	0.898 (-1.199)	NA	NA	0.867 (19.996)	0.949 (-0.424)	0.999 (0.004)
chr9	rs28728004	85560644	0.979 (-0.166)	0.978 (-0.025)	0.791 (0.238)	0.935 (-0.22)	0.998 (0.997)	0.821 (0.493)	0.602 (-2.206)	0.792 (0.299)	0.921 (-0.032)	0.995 (-0.002)	0.867 (-3.446)	0.884 (0.188)	0.999 (0.401)
chr9	rs372277219	93845307	0.979 (-0.307)	0.978 (-0.043)	0.808 (-0.294)	0.56 (-0.891)	0.998 (0.453)	0.821 (1.063)	0.946 (-0.189)	0.146 (2.916)	0.853 (-0.088)	0.995 (-0.166)	0.867 (1.91)	0.914 (-0.191)	0.999 (0.132)
chr10	rs10829909	131130644	0.142 (2.952)	0.978 (0.032)	0.765 (0.31)	0.935 (0.214)	0.998 (0.501)	0.821 (-1.152)	0.492 (2.667)	0.616 (0.62)	0.921 (-0.031)	0.995 (-0.041)	0.867 (-1.83)	0.613 (0.396)	0.999 (0.117)
chr12	rs74576183	6505015	0.967 (-1.033)	0.963 (0.05)	0.765 (0.355)	0.95 (0.112)	0.998 (1.208)	**0.046 (-2.981)**	0.803 (-0.9)	0.599 (0.617)	0.741 (0.13)	0.995 (0.086)	0.867 (-1.741)	0.819 (0.214)	0.999 (0.458)
chr12	rs536947	9979340	0.967 (0.638)	0.681 (0.128)	0.304 (0.606)	0.971 (0.058)	0.998 (0.826)	0.821 (-0.85)	0.461 (3.259)	0.886 (-0.191)	0.741 (0.149)	0.995 (0.064)	0.867 (2.038)	0.336 (0.594)	0.999 (0.318)
chr12	rs376870800	58569336	0.967 (-1.122)	0.963 (0.048)	0.908 (-0.049)	0.935 (0.238)	0.998 (-0.791)	0.821 (-0.79)	0.891 (0.313)	0.622 (-0.558)	0.741 (0.105)	0.995 (0.331)	0.867 (1.663)	0.949 (0.023)	0.999 (-0.267)
chr12	rs1032698711	103794720	0.967 (-0.864)	1.0 (0.006)	0.765 (0.294)	0.971 (0.093)	0.998 (0.911)	0.97 (-0.075)	0.602 (-2.244)	0.792 (0.326)	0.921 (-0.024)	0.995 (0.065)	0.867 (-4.889)	0.719 (0.313)	0.999 (0.381)
chr12	rs75047318	120431276	0.982 (0.072)	0.681 (0.315)	0.166 (1.138)	0.935 (-0.354)	0.998 (2.082)	0.821 (-1.399)	0.785 (-1.687)	0.193 (2.707)	0.505 (0.464)	0.995 (0.014)	0.867 (5.472)	0.114 (1.199)	0.999 (0.71)
chr13	rs1033760372	47662268	0.967 (-1.619)	0.681 (-0.174)	0.882 (-0.132)	0.352 (-0.839)	0.998 (-0.436)	0.821 (1.06)	0.183 (-5.833)	0.567 (0.849)	0.741 (-0.187)	0.995 (-0.385)	0.867 (-5.542)	0.949 (-0.133)	0.999 (-0.138)
chr13	rs537391125	61791792	0.365 (-4.248)	0.87 (0.193)	0.166 (-1.177)	0.875 (-0.678)	0.998 (-2.552)	0.821 (0.902)	0.616 (-3.697)	0.536 (1.798)	0.853 (0.164)	0.995 (1.434)	0.867 (-3.936)	0.114 (-1.152)	0.999 (-0.96)
chr13	rs72634302	89848283	0.967 (1.688)	0.681 (-0.18)	0.882 (0.133)	0.935 (-0.142)	0.998 (0.668)	0.821 (-1.368)	0.785 (1.14)	0.754 (0.348)	0.505 (-0.284)	0.995 (-0.158)	0.867 (2.332)	0.914 (0.153)	0.999 (0.205)
chr13	rs16965509	104870821	0.967 (-0.311)	0.87 (-0.076)	0.882 (-0.143)	0.971 (-0.067)	0.998 (-1.141)	0.821 (0.633)	0.461 (-2.838)	0.886 (-0.19)	0.741 (-0.091)	0.995 (-0.125)	0.959 (-0.393)	0.751 (-0.243)	0.999 (-0.424)
chr14	rs1594377001	37197894	0.967 (0.499)	0.681 (0.376)	0.765 (0.469)	0.875 (-0.621)	0.998 (0.859)	0.821 (1.933)	0.7 (-2.881)	0.657 (0.906)	0.741 (0.352)	0.995 (1.025)	0.867 (-8.996)	0.751 (0.458)	0.999 (0.311)
chr14	rs1033848215	60160438	0.967 (0.786)	0.963 (0.052)	0.765 (-0.249)	0.935 (0.143)	0.949 (2.394)	0.877 (-0.211)	0.848 (-0.828)	0.343 (-1.442)	0.895 (0.057)	0.995 (0.024)	0.867 (1.007)	0.751 (-0.255)	0.727 (0.889)
chr14	rs12885954	77524491	0.979 (0.113)	0.681 (0.153)	0.882 (-0.065)	0.935 (-0.151)	0.998 (0.691)	0.821 (-0.536)	0.481 (2.535)	0.657 (-0.438)	0.741 (0.106)	0.375 (1.053)	0.994 (0.065)	0.926 (-0.127)	0.999 (0.291)
chr14	rs8003454	92251093	0.967 (0.86)	0.681 (0.121)	0.882 (-0.073)	0.935 (0.229)	0.998 (0.056)	0.821 (-0.461)	0.672 (-1.721)	0.562 (-0.815)	0.741 (0.117)	0.995 (0.294)	0.867 (-1.4)	0.949 (0.026)	0.999 (-0.034)
chr16	rs2287123	87839137	0.967 (-0.526)	0.681 (-0.179)	0.628 (-0.483)	0.56 (-0.603)	0.998 (-0.585)	0.821 (0.877)	0.183 (-5.312)	0.567 (0.833)	0.505 (-0.367)	0.995 (0.12)	0.867 (-4.503)	0.613 (-0.395)	0.999 (-0.24)
chr17	rs575590765	54041157	0.982 (-0.031)	0.849 (-0.136)	0.765 (-0.357)	0.971 (-0.06)	0.998 (-0.197)	0.821 (-1.474)	0.593 (-3.239)	0.656 (-0.728)	0.741 (-0.173)	0.995 (0.472)	0.867 (-4.536)	0.613 (-0.503)	0.999 (0.011)
chr18	rs371858795	4162760	0.967 (-0.696)	0.681 (0.199)	0.882 (0.141)	0.865 (0.48)	0.998 (-0.618)	0.386 (2.101)	0.672 (-1.703)	0.935 (0.074)	0.598 (0.239)	0.995 (-0.346)	0.867 (-4.403)	0.863 (0.217)	0.999 (-0.186)
chr18	rs2848745	41477761	0.967 (0.505)	0.681 (-0.173)	0.882 (0.144)	0.989 (-0.006)	0.998 (0.198)	0.863 (-0.383)	0.672 (2.218)	0.463 (1.556)	0.741 (-0.188)	0.995 (-0.094)	0.867 (3.788)	0.914 (0.187)	0.999 (0.095)
chr19	rs900717974	12067828	0.979 (0.281)	0.67 (0.386)	0.882 (-0.13)	0.971 (-0.052)	0.998 (-0.005)	0.821 (-2.417)	0.461 (-6.081)	0.487 (-1.935)	0.55 (0.345)	0.995 (-0.307)	0.994 (-0.039)	0.949 (-0.134)	0.999 (0.054)
chr19	rs1870071	16394295	0.967 (-0.384)	0.694 (-0.122)	0.791 (-0.252)	0.966 (-0.107)	0.998 (0.185)	0.858 (0.327)	0.461 (-3.401)	0.343 (1.473)	0.741 (-0.136)	0.995 (-0.159)	0.867 (-2.915)	0.613 (-0.376)	0.999 (0.068)
chr19	.	54200712	0.967 (1.642)	0.963 (0.09)	0.882 (-0.144)	0.56 (-1.005)	0.998 (0.223)	0.821 (1.082)	0.461 (-5.023)	0.599 (-1.232)	0.934 (0.023)	0.995 (0.457)	0.867 (-8.173)	0.949 (0.035)	0.999 (0.218)
chr22	.	45617645	0.967 (0.644)	0.67 (0.423)	0.969 (0.037)	0.935 (-0.253)	0.998 (1.862)	0.857 (0.768)	0.799 (2.056)	0.935 (-0.111)	0.505 (0.475)	0.995 (1.023)	0.867 (2.274)	0.949 (-0.133)	0.999 (0.601)

Beta's are given considering the derived allele as the effect allele

*residuals for age and gender

** residuals for age, gender and height

beta in absolute value when we don't have the ancestral allele information

X some genotype groups are too small to allow comparison

in bold, pval < 0.05

Table S14: Candidate SNPs driving selection in PNG highlanders with significant eQTL in the GTEx Portal
P-values and Normalized effect size were accessed from the GTEx portal (https://www.gtexportal.org)

Candidate SNP	Gene	P-Value	Normalized effect size	Tissue
**rs74621527**	TNFAIP6	1.70E-13	0.34	Whole Blood
	TNFAIP6	6.70E-12	0.49	Cells - Cultured fibroblasts
	NMI	1.10E-07	0.39	Cells - Cultured fibroblasts
	RBM43	2.20E-06	0.24	Whole Blood
	NMI	1.90E-04	-0.21	Thyroid
**rs9277772**	HLA-DPA1	2.60E-23	0.85	Cells - Cultured fibroblasts
	HCG24	1.70E-20	1.20	Brain - Cerebellum
	HCG24	5.70E-19	0.97	Brain - Nucleus accumbens (basal ganglia)
	HCG24	1.90E-18	1.00	Brain - Caudate (basal ganglia)
	HLA-DPB2	1.90E-15	-0.46	Whole Blood
	HCG24	4.70E-14	1.00	Brain - Cerebellar Hemisphere
	COL11A2	1.30E-11	0.68	Brain - Cerebellum
	HLA-DPB2	2.50E-11	-0.51	Nerve - Tibial
	HLA-DPB2	7.00E-10	-0.46	Lung
	HLA-DPB2	2.40E-09	-0.42	Thyroid
	HLA-DPB2	5.30E-09	-0.58	Brain - Hypothalamus
	COL11A2	5.70E-09	0.66	Brain - Cerebellar Hemisphere
	HLA-DPB2	8.10E-09	-0.47	Adipose - Subcutaneous
	HCG24	1.50E-08	0.59	Brain - Hypothalamus
	HLA-DPB2	1.70E-08	-0.84	Cells - EBV-transformed lymphocytes
	HLA-DPB2	3.20E-08	-0.38	Artery - Tibial
	HCG24	3.90E-08	0.68	Brain - Putamen (basal ganglia)
	HLA-DPB2	5.60E-08	-0.71	Brain - Cerebellum
	HLA-DPB2	9.60E-08	-0.46	Skin - Not Sun Exposed (Suprapubic)
	HLA-DPB2	2.00E-07	-0.54	Colon - Sigmoid
	HLA-DPB2	9.00E-07	-0.39	Esophagus - Mucosa
	RPL32P1	1.50E-06	0.32	Adipose - Subcutaneous
	HLA-DPA1	1.50E-06	0.32	Testis
	HLA-DPB2	1.80E-06	-0.54	Pituitary
	HLA-DPB2	2.40E-06	-0.69	Brain - Cerebellar Hemisphere
	HLA-DPB2	2.90E-06	-0.38	Esophagus - Muscularis
	HLA-DPB2	3.90E-06	-0.40	Artery - Aorta
	HLA-DPA3	7.10E-06	0.62	Brain - Cerebellum
	HLA-DPB2	7.60E-06	-0.34	Skin - Sun Exposed (Lower leg)
	HLA-DPB2	8.20E-06	-0.45	Small Intestine - Terminal Ileum
	WDR46	9.30E-06	0.10	Cells - Cultured fibroblasts
	WDR46	1.50E-05	0.36	Brain - Cerebellum
	RXRB	1.50E-05	0.31	Brain - Cerebellum
	WDR46	1.70E-05	0.10	Lung
	HLA-DPB2	1.90E-05	-0.43	Breast - Mammary Tissue
	RPL32P1	1.90E-05	0.27	Skin - Not Sun Exposed (Suprapubic)
	RPL32P1	2.40E-05	0.32	Esophagus - Muscularis
	CUTA	2.60E-05	-0.10	Artery - Tibial
	HCG24	2.70E-05	0.39	Brain - Hippocampus
	RPL32P1	4.00E-05	0.35	Breast - Mammary Tissue
	RPL32P1	4.30E-05	0.27	Skin - Sun Exposed (Lower leg)
	TAPBP	4.60E-05	0.14	Nerve - Tibial
	WDR46	4.60E-05	0.09	Muscle - Skeletal
	RING1	7.50E-05	-0.15	Thyroid
	CUTA	7.90E-05	-0.14	Adipose - Visceral (Omentum)
	CUTA	9.00E-05	-0.13	Skin - Not Sun Exposed (Suprapubic)
	RPL32P1	1.20E-04	0.22	Nerve - Tibial
	CUTA	1.50E-04	-0.11	Nerve - Tibial
**rs74576183**	ACRBP	1.30E-05	-0.65	Skin - Sun Exposed (Lower leg)
**rs536947**	CLEC12A	5.60E-09	-0.37	Cells - Cultured fibroblasts
	CLEC12A	1.00E-08	-0.46	Heart - Atrial Appendage
	CLEC12A	1.10E-08	-0.40	Muscle - Skeletal
	CLEC12A	1.40E-08	-0.35	Lung
	CLEC12B	1.50E-08	-0.46	Nerve - Tibial
	CLEC1B	4.50E-08	-0.45	Nerve - Tibial
	CLEC12B	4.90E-08	-0.33	Whole Blood
	CLEC12A	6.30E-08	-0.39	Adipose - Subcutaneous
	CLEC12A	1.00E-07	-0.43	Nerve - Tibial
	CLEC12B	6.10E-07	-0.35	Adipose - Subcutaneous
	CLEC12A	8.50E-07	-0.26	Whole Blood
	CLEC12A	7.80E-06	-0.30	Thyroid
	CLEC12A	1.20E-05	-0.36	Esophagus - Muscularis
	CLEC12B	1.90E-05	-0.28	Lung
	CLEC1B	4.10E-05	-0.30	Adipose - Subcutaneous
	CLEC12A	5.00E-05	-0.38	Breast - Mammary Tissue
	CLEC12A	6.60E-05	-0.27	Artery - Tibial
	CLEC12A	7.60E-05	-0.26	Skin - Sun Exposed (Lower leg)
	CLEC12B	1.20E-04	-0.32	Esophagus - Muscularis
**rs8003454**	SLC24A4	6.70E-06	-0.25	Brain - Cerebellum

Table S15: Candidate SNPs driving selection in PNG lowlanders with significant eQTL in the GTEx Portal
P-values and Normalized effect size were accessed from the GTEx portal (https://www.gtexportal.org)

Candidate SNP	Gene	P-Value	Normalized effect size	Tissue
**rs4693058**	THAP9-AS1	3.40E-15	0.31	Cells - Cultured fibroblasts
	THAP9-AS1	8.50E-14	0.19	Artery - Tibial
	THAP9-AS1	4.80E-12	0.19	Muscle - Skeletal
	SCD5	1.40E-09	-0.18	Whole Blood
	THAP9-AS1	3.00E-09	0.37	Brain - Hippocampus
	THAP9	3.70E-09	0.16	Nerve - Tibial
	THAP9-AS1	4.10E-07	0.11	Esophagus - Muscularis
	THAP9-AS1	1.30E-06	0.33	Cells - EBV-transformed lymphocytes
	THAP9-AS1	1.70E-06	0.08	Thyroid
	LIN54	1.90E-06	0.21	Spleen
	THAP9-AS1	6.00E-06	0.12	Artery - Aorta
	THAP9-AS1	6.00E-06	0.09	Lung
	THAP9-AS1	7.60E-06	0.22	Brain - Cortex
	THAP9-AS1	8.70E-06	0.10	Adipose - Subcutaneous
	THAP9-AS1	1.20E-05	0.29	Brain - Putamen (basal ganglia)
	THAP9-AS1	1.40E-05	0.29	Brain - Hypothalamus
	THAP9-AS1	2.10E-05	0.26	Brain - Nucleus accumbens (basal ganglia)
	THAP9	2.10E-05	0.18	Esophagus - Gastroesophageal Junction
	THAP9	2.10E-05	0.20	Pituitary
	THAP9	2.60E-05	0.19	Heart - Atrial Appendage
	THAP9-AS1	4.40E-05	0.19	Brain - Cerebellum
	THAP9	8.80E-05	0.10	Thyroid
	THAP9-AS1	1.80E-04	0.07	Nerve - Tibial
**rs12885954**	GSTZ1	1.10E-05	0.22	Skin - Sun Exposed (Lower leg)
	GSTZ1	4.00E-05	0.17	Lung
**rs1870071**	CTD-2013N17.6	4.90E-17	-0.20	Thyroid
	EPS15L1	2.10E-11	0.21	Esophagus - Muscularis
	CTD-2013N17.6	4.50E-10	-0.15	Skin - Sun Exposed (Lower leg)
	CTD-2013N17.6	2.30E-09	-0.28	Testis
	C19orf44	2.50E-09	-0.18	Thyroid
	C19orf44	3.00E-08	-0.20	Artery - Tibial
	C19orf44	5.50E-08	-0.18	Breast - Mammary Tissue
	CTD-2013N17.6	5.70E-08	-0.14	Skin - Not Sun Exposed (Suprapubic)
	C19orf44	7.20E-08	-0.22	Nerve - Tibial
	C19orf44	7.90E-08	-0.15	Muscle - Skeletal
	SLC35E1	1.80E-07	0.14	Testis
	C19orf44	3.20E-07	-0.14	Adipose - Visceral (Omentum)
	CTD-2013N17.6	6.30E-07	-0.10	Whole Blood
	C19orf44	6.70E-07	-0.21	Artery - Aorta
	C19orf44	8.00E-07	-0.17	Skin - Not Sun Exposed (Suprapubic)
	C19orf44	4.10E-06	-0.13	Skin - Sun Exposed (Lower leg)
	C19orf44	8.30E-06	-0.18	Esophagus - Muscularis
	C19orf44	1.40E-05	-0.16	Stomach
	CTD-2013N17.6	1.50E-05	-0.12	Adipose - Subcutaneous
	EPS15L1	2.00E-05	0.11	Artery - Tibial
	C19orf44	3.70E-05	-0.24	Adrenal Gland
	C19orf44	3.90E-05	-0.13	Esophagus - Mucosa
	C19orf44	4.80E-05	-0.19	Heart - Left Ventricle
	C19orf44	4.90E-05	-0.13	Adipose - Subcutaneous
	C19orf44	5.50E-05	-0.23	Pancreas
	CTD-3222D19.9	1.10E-04	0.18	Thyroid
	EPS15L1	1.30E-04	0.07	Muscle - Skeletal

Table S16: VEP results for the SNPs in LD with the candidate SNPs (R^2 >= 0.5) in PNG highlanders in a window of 500kb flanking the core SNP

Candidate SNP*	total snps	reg snps	regulatory region variant	TF binding site variant	5 prime UTR variant	missense variant	3 prime UTR variant	splice polypyrimidine tract variant	splice region variant
rs887476833	20	NA	NA	NA	NA	NA	NA	NA	NA
rs74621527	75	15	15	NA	NA	NA	NA	NA	NA
rs374181005	2	NA	NA	NA	NA	NA	NA	NA	NA
rs79600167	67	30	30	1	NA	NA	NA	NA	NA
rs943845085	1	NA	NA	NA	NA	NA	NA	NA	NA
rs369030953	7	2	1	NA	NA	1	1	NA	NA
rs940110341	30	14	13	NA	1	NA	NA	NA	NA
rs9277772	166	81	77	14	NA	1	1	2	1
rs17170618	130	24	24	NA	NA	NA	NA	NA	NA
rs28728004	57	13	13	1	NA	NA	NA	NA	NA
rs10829909	42	18	18	NA	NA	NA	NA	NA	NA
rs74576183	29	17	17	4	1	1	NA	NA	NA
rs536947	26	9	8	NA	NA	NA	2	NA	NA
rs376870800	73	19	19	2	NA	NA	NA	NA	NA
rs1032698711	5	2	2	NA	NA	NA	NA	NA	NA
rs1033760372	25	2	2	NA	NA	NA	NA	NA	NA
rs16965509	42	1	1	NA	NA	NA	NA	NA	NA
rs1033848215	169	37	34	3	NA	1	2	1	NA
rs8003454	17	1	1	NA	NA	NA	NA	NA	NA
rs371858795	36	4	4	NA	NA	NA	NA	NA	NA
rs1601558750	1	NA	NA	NA	NA	NA	NA	NA	NA

*When no rs id given we gave the GRCh38 coordinate of the SNP

regSNPs = SNPs with consequenced in the genomic region, regulatory region variant = SNP located within a regulatory region, TF binding site variant=SNP located within a transcription factor binding site 5 prime UTR variant =SNP that are UTR variant of the 5' UTR, 3 prime UTR variant = SNP that are UTR variant of the 3' UTR, missense variant = SNP that changes one or more bases, resulting in a different amino acid sequence but where the length is preserved, Splice polypyrimidine tract variant = SNP that falls in the polypyrimidine tract at 3' end of intron between 17 and 3 bases from the end (acceptor -3 to acceptor -17), splice region variant = SNP in which a change has occurred within the region of the splice site, either within 1-3 bases of the exon or 3-8 bases of the intron

Table S17: VEP results for the SNPs in LD with the candidate SNPs (r^2>= 0.5) in PNG lowlanders in a window of 500kb flanking the core SNP

Candidate SNP*	total snps	reg snps	regulatory region variant	TF binding site variant	5 prime UTR variant	missense variant	3 prime UTR variant	splice polypyrimidine tract variant	splice region variant
rs368120563	213	40	26	2	NA	4	9	NA	NA
rs1574154373	7	2	2	NA	NA	NA	NA	NA	NA
rs7583123	94	4	4	NA	NA	NA	NA	NA	NA
chr2:200269472	3	1	1	NA	NA	NA	NA	NA	NA
rs376150658	2	1	1	NA	NA	NA	NA	NA	NA
rs926184421	68	20	18	NA	NA	NA	2	NA	NA
rs4693058	4	NA	NA	NA	NA	NA	NA	NA	NA
rs36003688	58	18	15	1	1	NA	6	NA	NA
rs989789809	1	NA	NA	NA	NA	NA	NA	NA	NA
rs6950082	1	NA	NA	NA	NA	NA	NA	NA	NA
rs187915256	3	NA	NA	NA	NA	NA	NA	NA	NA
rs372277219	2	NA	NA	NA	NA	NA	NA	NA	NA
rs75047318	10	4	4	NA	NA	NA	NA	NA	NA
rs537391125	7	NA	NA	NA	NA	NA	NA	NA	NA
rs72634302	13	NA	NA	NA	NA	NA	NA	NA	NA
rs1594377001	7	2	2	NA	1	NA	NA	NA	NA
rs12885954	35	7	6	1	NA	NA	1	NA	NA
rs2287123	9	3	3	NA	NA	NA	NA	NA	NA
rs575590765	2	NA	NA	NA	NA	NA	NA	NA	NA
rs2848745	155	8	8	NA	NA	NA	NA	NA	NA
rs900717974	8	1	NA	NA	NA	NA	1	NA	NA
rs1870071	15	3	3	NA	NA	NA	NA	NA	NA
rs1600734199	2	2	2	1	NA	NA	NA	NA	NA

*When no rs id given we gave the GRCh38 coordinate of the SNP

regSNPs = SNPs with consequenced in the genomic region, regulatory region variant = SNP located within a regulatory region, TF binding site variant=SNP located within a transcription factor binding site 5 prime UTR variant =SNP that are UTR variant of the 5' UTR, 3 prime UTR variant = SNP that are UTR variant of the 3' UTR, missense variant = SNP that changes one or more bases, resulting in a different amino acid sequence but where the length is preserved, Splice polypyrimidine tract variant = SNP that falls in the polypyrimidine tract at 3' end of intron between 17 and 3 bases from the end (acceptor -3 to acceptor -17), splice region variant = SNP in which a change has occurred within the region of the splice site, either within 1-3 bases of the exon or 3-8 bases of the intron

Table S18: Missense variants in the region under selection in PNG highlanders and their DAF among different populations

Genomic region under selection (GRCh38)	Variant pos	Missense variant	PNG HL	PNG LL	PNG diversity I	GBR	KHV	ESN	CEU	YRI	CHB	Nean	Deni
**chr3:13010340-13217789**	chr3:13022091	IQSEC1-Y4D*	0.44	0.19	0.22	0.15	0.14	0.01	0.09	0.03	0.17	0.00	0.00
**chr4:152704503-152970509**	chr4:152769690	TIGD4-I439T	0.15	0.13	0.11	0.35	0.30	0.30	0.43	0.24	0.33	0.00	0.00
	chr4:152953514	FHDC1-A172R	0.69	0.42	0.55	0.01	0.07	0.15	0.01	0.15	0.11	0.00	0.00
**chr6:30916070-31153184**	chr6:30920384	VARS2-W449Q	0.38	0.15	0.31	0.46	0.15	0.71	0.46	0.73	0.24	0.50	1.00
	chr6:30925350	VARS2-R917Q	0.61	0.73	0.61	0.21	0.69	0.17	0.18	0.19	0.47	0.50	0.00
	chr6:30926164	VARS2-R1049R	0.30	0.07	0.15	0.19	0.03	0.06	0.18	0.11	0.07	0.50	0.50
	chr6:30938138	DPCR1-H75Y*	0.07	0.07	0.13	0.03	0.03	0.00	0.02	0.00	0.04	0.00	0.00
	chr6:30948928	DPCR1-K155R	0.07	0.14	0.15	0.00	0.10	0.00	0.00	0.00	0.01	0.00	0.00
	chr6:30952101	DPCR1-G1213G	0.07	0.07	0.14	0.12	0.04	0.51	0.13	0.46	0.06	0.00	0.00
	chr6:31025756	MUC22-R109S	0.88	0.59	0.65	0.51	0.37	0.42	0.54	0.53	0.30	0.00	0.00
	chr6:31025999	MUC22-T190N	0.19	0.24	0.20	0.10	0.05	0.35	0.08	0.22	0.15	0.00	0.00
	chr6:31026036	MUC22-S202S	0.10	0.09	0.12	0.19	0.07	0.02	0.15	0.01	0.07	0.00	0.00
	chr6:31026089	MUC22-T220I	0.10	0.09	0.12	0.16	0.06	0.03	0.13	0.03	0.06	0.00	0.00
	chr6:31027401	MUC22-T657T	0.09	0.09	0.11	0.15	0.06	0.03	0.10	0.03	0.07	0.00	0.00
	chr6:31027410	MUC22-I660V	0.91	0.91	0.89	0.85	0.94	0.97	0.90	0.97	0.93	1.00	1.00
	chr6:31027520	MUC22-I697T	0.08	0.09	0.12	0.15	0.06	0.03	0.10	0.03	0.07	0.00	0.00
	chr6:31027755	MUC22-M775I	0.90	0.91	0.88	0.85	0.94	0.97	0.90	0.97	0.93	1.00	1.00
	chr6:31028355	MUC22-T975A	0.09	0.09	0.12	0.15	0.06	0.03	0.10	0.03	0.07	0.00	0.00
	chr6:31029504	MUC22-V1358A	0.99	1.00	1.00	1.00	1.00	1.00	1.00	1.00	1.00	1.00	1.00
	chr6:31029557	MUC22-T1376G	0.10	0.09	0.12	0.15	0.06	0.03	0.10	0.03	0.07	0.00	0.00
	chr6:31029575	MUC22-S1382P	0.90	0.91	0.88	0.85	0.94	0.97	0.90	0.96	0.93	1.00	1.00
	chr6:31030047	MUC22-L1539P	0.30	0.57	0.65	0.85	0.87	0.86	0.90	0.77	0.84	1.00	1.00
	chr6:31032220	MUC22-L1565D	0.07	0.22	0.32	0.60	0.62	0.62	0.67	0.50	0.44	0.50	0.00
	chr6:31034675	MUC22-N1687D	0.07	0.22	0.28	0.48	0.64	0.38	0.54	0.36	0.36	0.50	0.00
	chr6:31034750	MUC22-N1712Q	0.68	0.55	0.55	0.54	0.73	0.34	0.61	0.33	0.55	0.00	0.00
	chr6:31054381	HCG22-E18V	0.10	0.32	0.24	0.36	0.37	0.43	0.34	0.55	0.44	1.00	1.00
	chr6:31054418	HCG22-A30R	0.10	0.13	0.12	0.25	0.18	0.08	0.22	0.22	0.18	0.00	0.00
	chr6:31054429	HCG22-G34E	0.86	0.82	0.76	0.71	0.72	0.83	0.75	0.71	0.77	1.00	1.00
	chr6:31054460	HCG22-G44P	0.09	0.12	0.12	0.25	0.18	0.08	0.22	0.22	0.18	0.00	0.00
	chr6:31054489	HCG22-A54D	0.14	0.17	0.24	0.29	0.28	0.17	0.25	0.28	0.23	0.00	0.00
	chr6:31111487	C6orf15-G291E	0.08	0.20	0.12	0.08	0.18	0.00	0.05	0.00	0.19	0.50	0.50
	chr6:31111866	C6orf15-K165P	0.69	0.61	0.56	0.51	0.52	0.43	0.53	0.39	0.51	0.50	0.00
	chr6:31111926	C6orf15-A145P	0.25	0.31	0.25	0.09	0.30	0.04	0.07	0.06	0.37	0.50	0.50
	chr6:31112112	C6orf15-A83A	0.70	0.61	0.56	0.51	0.52	0.43	0.53	0.39	0.51	0.00	0.00
	chr6:31112117	C6orf15-V81D	0.76	0.69	0.75	0.82	0.70	0.93	0.82	0.92	0.63	0.00	0.50
	chr6:31116036	CDSN-N527S	0.29	0.28	0.34	0.25	0.16	0.07	0.26	0.11	0.14	0.00	0.00
	chr6:31116386	CDSN-L410A	0.71	0.71	0.64	0.55	0.71	0.75	0.48	0.63	0.67	0.50	0.00
	chr6:31116393	CDSN-S408D	0.12	0.25	0.23	0.16	0.34	0.09	0.15	0.09	0.16	0.50	0.00
	chr6:31116713	CDSN-G301S	0.09	0.00	0.02	0.00	0.00	0.00	0.00	0.00	0.00	0.00	0.00
	chr6:31117010	CDSN-F202S	0.17	0.20	0.24	0.26	0.25	0.48	0.22	0.41	0.27	0.00	0.00
	chr6:31117187	CDSN-N143S	0.29	0.28	0.34	0.25	0.16	0.07	0.26	0.11	0.14	0.00	0.00
	chr6:31138211	PSORS1C2-G51T	0.22	0.07	0.12	0.00	0.14	0.00	0.00	0.00	0.07	0.00	0.00
	chr6:31138682	PSORS1C1-P24S	0.08	0.04	0.12	0.10	0.04	0.11	0.07	0.19	0.06	0.00	0.00
	chr6:31138739	PSORS1C1-P43C	0.67	0.44	0.33	0.03	0.17	0.11	0.04	0.18	0.14	0.00	0.00
	chr6:31142614	CCHCR1-S865H	0.08	0.04	0.12	0.31	0.09	0.24	0.30	0.22	0.14	0.00	0.00
	chr6:31143397	CCHCR1-Q728Q	0.67	0.51	0.34	0.03	0.18	0.12	0.04	0.18	0.15	0.00	0.00
	chr6:31144707	CCHCR1-R716R	0.08	0.04	0.12	0.08	0.04	0.02	0.06	0.07	0.06	0.00	0.00
	chr6:31145046	CCHCR1-K635W	0.42	0.26	0.16	0.00	0.06	0.05	0.00	0.11	0.08	0.00	0.00
	chr6:31148469	CCHCR1-R506D	0.75	0.65	0.53	0.43	0.39	0.38	0.41	0.44	0.33	0.00	0.50
	chr6:31150734	CCHCR1-E364Q	0.19	0.24	0.29	0.24	0.35	0.15	0.22	0.20	0.28	0.00	0.50
	chr6:31151121	CCHCR1-L268S	0.08	0.04	0.12	0.08	0.04	0.00	0.06	0.00	0.06	0.00	0.00
**chr9:85458922-85745092**	chr9:85692760	AGTPBP1-N29V	0.08	0.00	0.02	0.00	0.00	0.00	0.00	0.00	0.00	0.00	0.00
**chr12:6452552-6662260**	chr12:6453127	TAPBPL-A42R	0.10	0.17	0.21	0.33	0.31	0.08	0.25	0.08	0.30	0.00	0.00
	chr12:6453602	TAPBPL-G151R	0.70	0.46	0.56	0.01	0.10	0.06	0.02	0.04	0.11	0.00	0.00
	chr12:6453657	TAPBPL-A169E	0.10	0.17	0.21	0.34	0.31	0.17	0.25	0.17	0.30	0.00	1.00
	chr12:6510118	NCAPD2-Q83S	0.06	0.17	0.18	0.30	0.33	0.01	0.25	0.00	0.29	0.00	0.00
	chr12:6530815	NCAPD2-T1321L	0.06	0.03	0.07	0.25	0.35	0.05	0.18	0.02	0.43	0.00	0.00
	chr12:6555911	IFFO1-P40I	0.06	0.02	0.04	0.00	0.00	0.00	0.00	0.00	0.00	0.00	0.00
**chr12:9886812-10055333**	chr12:9888765	KLRF2-V68T	0.11	0.28	0.11	0.18	0.22	0.38	0.21	0.45	0.30	0.00	0.00
	chr12:9893453	KLRF2-P131D	0.82	0.59	0.74	0.74	0.49	0.31	0.71	0.28	0.45	0.00	0.00
	chr12:9916703	CLEC2A-G136	0.37	0.57	0.42	0.35	0.67	0.46	0.31	0.52	0.69	0.00	0.00

*missense name is non-canonical

Table S19: Missense variants in the region under selection in PNG lowlanders and their DAF among different populations

Genomic region under selection (GRCh38)	Variant pos	Missense variant	PNG HL	PNG LL	PNG diversity I	GBR	KHV	ESN	CEU	YRI	CHB	Nean	Deni
**chr1:88800562-89326878**	chr1:89009135	GBP3-C491T	0.92	0.97	0.93	0.72	0.81	0.77	0.64	0.77	0.77	1.00	1.00
	chr1:89109691	GBP2-A549T	0.53	0.82	0.56	0.00	0.00	0.00	0.00	0.00	0.00	1.00	1.00
	chr1:89132390	GBP7-L559R	0.47	0.11	0.43	1.00	1.00	1.00	1.00	1.00	1.00	0.00	0.00
	chr1:89150509	GBP7-Q231I	0.46	0.11	0.44	1.00	1.00	0.96	1.00	0.94	1.00	0.00	0.00
	chr1:89171895	GBP7-T14K	0.23	0.06	0.08	0.34	0.23	0.25	0.36	0.17	0.22	0.00	0.00
	chr1:89186404	GBP4-E546I	0.21	0.05	0.33	0.45	0.39	0.11	0.45	0.11	0.40	0.00	0.00
	chr1:89186405	GBP4-M545L	0.21	0.05	0.33	0.45	0.39	0.11	0.45	0.11	0.40	0.00	0.00
	chr1:89186407	GBP4-M545I	0.21	0.05	0.33	0.45	0.39	0.11	0.45	0.11	0.40	0.00	0.00
	chr1:89186414	GBP4-M542N	0.21	0.05	0.33	0.45	0.39	0.11	0.45	0.11	0.40	0.00	0.00
	chr1:89186419	GBP4-Y541R	0.21	0.05	0.33	0.45	0.39	0.11	0.45	0.11	0.40	0.00	0.00
	chr1:89188617	GBP4-W459L	0.47	0.14	0.44	1.00	1.00	1.00	1.00	1.00	1.00	0.00	0.00
**chr2:200238798-200432145**	chr2:200389233	chr2:200389233*	0.35	0.09	0.25	0.31	0.13	0.25	0.30	0.21	0.19	0.00	0.00
**chr2:241759136-242088831**	chr2:241776965	GAL3ST2-M4V	0.20	0.24	0.21	0.27	0.35	0.59	0.30	0.60	0.39	0.00	0.00
	chr2:241803460	GAL3ST2-A164R	0.60	0.61	0.60	0.11	0.24	0.14	0.11	0.16	0.15	1.00	0.00
	chr2:241814177	NEU4-P30R*	0.49	0.78	0.63	0.01	0.37	0.00	0.00	0.00	0.49	0.50	0.00
	chr2:241816494	NEU4-G301S	0.33	0.22	0.24	0.37	0.09	0.28	0.22	0.35	0.09	0.00	0.00
	chr2:241816965	NEU4-R458V	0.03	0.09	0.10	0.00	0.00	0.00	0.00	0.00	0.00	0.00	0.00
	chr2:241851281	PDCD1-A215T	0.51	0.73	0.56	0.01	0.38	0.00	0.02	0.01	0.50	1.00	0.00
**chr4:82750503-83146792**	chr4:82916988	THAP9-I259F	0.00	0.06	0.01	0.00	0.00	0.00	0.00	0.00	0.00	0.00	0.00
	chr4:82917109	THAP9-L299T	0.65	0.84	0.71	0.59	0.17	0.04	0.52	0.06	0.23	0.00	0.00
**chr12:120353731-120666335**	chr12:120516687	COQ5-A152K	0.28	0.07	0.10	0.11	0.09	0.27	0.09	0.28	0.08	0.00	0.00
	chr12:120655947	CABP1-A46G*	0.01	0.07	0.02	0.00	0.00	0.00	0.00	0.00	0.00	0.00	0.00
**chr14:77312867-77558267**	chr14:77326864	GSTZ1-E32T	0.90	0.83	0.73	0.34	0.47	0.31	0.29	0.34	0.51	1.00	0.00
	chr14:77327940	GSTZ1-M82P	0.07	0.11	0.03	0.23	0.05	0.08	0.20	0.07	0.04	0.00	0.00
	chr14:77377020	TMED8-S12P	0.41	0.12	0.12	0.20	0.03	0.09	0.21	0.05	0.02	0.00	0.00
	chr14:77377471	SAMD15-L18I	0.61	0.88	0.86	0.52	0.88	0.08	0.49	0.09	0.94	0.00	0.00
	chr14:77377922	SAMD15-M168E	0.41	0.11	0.12	0.20	0.03	0.05	0.21	0.03	0.02	0.00	0.00
	chr14:77378526	SAMD15-K370E	0.60	0.89	0.86	0.52	0.88	0.05	0.49	0.07	0.94	0.00	0.00
	chr14:77378778	SAMD15-K454N	0.60	0.89	0.86	0.52	0.88	0.08	0.49	0.09	0.94	0.00	0.00
	chr14:77476074	ISM2-D413T	0.06	0.05	0.04	0.00	0.00	0.00	0.00	0.00	0.00	0.00	0.00
	chr14:77484781	ISM2-A94V	1.00	0.98	0.97	0.65	0.94	0.14	0.60	0.12	0.97	0.00	0.00
	chr14:77521615	SPTLC2-G33V*	0.47	0.21	0.21	0.28	0.11	0.52	0.25	0.35	0.09	0.00	0.00
**chr19:11708670-12108034**	chr19:11722794	ZNF823-A247F	0.12	0.06	0.04	0.00	0.00	0.00	0.00	0.00	0.00	0.00	0.00
	chr19:11978549	ZNF763-V209H	0.51	0.86	0.83	0.99	1.00	0.97	0.98	0.96	0.99	0.00	0.00
	chr19:11978894	ZNF763-Y324M	0.49	0.86	0.83	0.99	1.00	0.97	0.98	0.96	0.99	0.00	0.00
	chr19:11978928	ZNF763-T335G	0.51	0.14	0.17	0.01	0.00	0.03	0.02	0.04	0.01	1.00	1.00
	chr19:11978990	ZNF763-R356A	0.50	0.14	0.17	0.01	0.00	0.03	0.02	0.04	0.01	1.00	1.00
	chr19:12075333	ZNF844-L51S*	0.19	0.45	0.46	0.89	0.91	0.28	0.83	0.26	0.95	0.00	0.00
	chr19:12075946	ZNF844-T276P	0.82	0.53	0.54	0.11	0.09	0.76	0.17	0.78	0.05	1.00	1.00
	chr19:12076042	ZNF844-A308W	0.18	0.46	0.46	0.89	0.91	0.24	0.83	0.22	0.95	0.00	0.00
	chr19:54231743	RPS9-G75R*	0.39	0.05	0.09	0.19	0.07	0.14	0.18	0.15	0.09	0.00	NA
**chr19:54176104-54330609**	chr19:54278849	LILRB2-C306	0.42	0.66	0.55	0.43	0.75	0.69	0.36	0.69	0.94	0.00	NA

*missense name is non-canonical

Table S20: Introgressed haplotypes with the highest frequency in regions under selection in PNG highlanders

Candidate regions for selection	introgressed haplotype	PNG population	introgressed haplotype origin	intogressed haplotype frequence	introgressed haplotype count	r^2
**chr1:95529290-95736826**	chr1:95581715-95682887*	LL	Denisovan	0.115	17	0.083
**chr2:151012094-151201575**	chr2:151077551-151194524	HL	Neanderthal	0.009	1	**0.574**
		LL	Neanderthal	0.047	7	
**chr3:13010340-13217789**	chr3:13132090-13174330	HL	Denisovan	0.333	36	0.043
		LL	Denisovan	0.142	21	
**chr3:61779523-62009858**	chr3:61798966-61853037	HL	Neanderthal	0.019	2	0.031
		LL	Neanderthal	0.020	3	
**chr4:110182324-110384099**	chr4:110232325-110334098	HL	Neanderthal	0.444	48	0.262
		LL	Neanderthal	0.155	23	
**chr6:30916070-31153184**	chr6:31077777-31112941	HL	ambiguous	0.083	9	0.096
		LL	ambiguous	0.196	29	
**chr6:33006055-33132312**	chr6:33092117-33115426*	LL	ambiguous	0.007	1	nan
**chr7:147590904-147718219**	chr7:147665094-147696027	HL	Denisovan	0.019	2	0.027
**chr10:131112245-131235951**	chr10:131130857-131157433	HL	Denisovan	0.046	5	0.036
**chr12:6452552-6662260**	chr12:6503185-6641651*	LL	Denisovan	0.020	3	nan
**chr12:9886812-10055333**	chr12:9904201-10023903	HL	Denisovan	0.046	5	0.149
		LL	Denisovan	0.068	10	
**chr12:58391529-58634980**	chr12:58451248-58568114	HL	ambiguous	0.620	67	**0.540**
		LL	ambiguous	0.405	60	
**chr12:103783315-104121479**	chr12:103839272-104061448	HL	Denisovan	0.556	60	0.297
		LL	Denisovan	0.196	29	
**chr13:104734734-104875020**	chr13:104787393-104824094	HL	Denisovan	0.528	57	0.034
		LL	Denisovan	0.243	36	
**chr14:60157772-60377317**	chr14:60220195-60315071*	LL	Denisovan	0.074	11	0.080
**chr14:92230479-92401520**	chr14:92370144-92392663	HL	ambiguous	0.028	3	0.018
		LL	ambiguous	0.074	11	
**chr18:4072997-4251153**	chr18:4136427-4203633	HL	Denisovan	0.583	63	**1**
		LL	Denisovan	0.385	57	

r^2 was computed on phased haplotypes from PNG highlanders for the candidate SNP and archaic SNP from the introgressed haplotype of the regions.
In some cases, introgressed haplotypes were only found in PNG lowlanders but not in PNG highlanders and none aSNP were found in PNG highlanders.
In those cases, R^2 could not be computed for PNG lolwanders and are indicated as nan
Candidate SNP in high LD (r^2>0.5) with at least one archaic SNP of the introgressed haplotype are in bold
*none introgressed haplotype was found in PNG highlanders in this region so we reported the introgressed haplotype with the highest frequency in PNG lowlanders

Table S21: Introgressed haplotypes with the highest frequency in regions under selection in PNG lowlanders

Candidate regions for selection	introgressed haplotype	PNG population	introgressed haplotype origin	intogressed haplotype frequence	introgressed haplotype count	r^2
**chr1:88800562-89326878**	chr1:89054418-89202534	HL	ambiguous	0.574	62	**0.943**
		LL	ambiguous	0.872	129	
**chr2:241759136-242088831**	chr2:241811883-241869518	HL	Neanderthal	0.546	59	0.092
		LL	Neanderthal	0.804	119	
**chr4:82750503-83146792**	chr4:82755644-83083169	HL	Denisovan	0.130	14	0.173
		LL	Denisovan	0.081	12	
**chr6:85266477-85483888**	chr6:85340299-85364688	LL	Denisovan	0.007	1	0.001
**chr7:129548370-129836070**	chr7:129553314-129774681	HL	Denisovan	0.028	3	0.000
		LL	Denisovan	0.041	6	
**chr8:133791891-133962825**	chr8:133841743-133891832*	HL	Denisovan	0.019	2	0.000
**chr9:93717217-93877803**	chr9:93752325-93867864	HL	Neanderthal	0.065	7	0.052
		LL	Neanderthal	0.101	15	
**chr12:120353731-120666335**	chr12:120368947-120395906	HL	ambiguous	0.083	9	0.008
		LL	ambiguous	0.081	12	
**chr17:54003406-54222843**	chr17:54036011-54160414	HL	Denisovan	0.046	5	0.029
		LL	Denisovan	0.007	1	
**chr19:11708670-12108034**	chr19:11887941-12085466	HL	Neanderthal	0.269	29	0.022
		LL	Neanderthal	0.095	14	

r^2 was computed on phased haplotypes from PNG lowlanders for the candidate SNP and archaic SNP from the introgressed haplotype of the regions.
Candidate SNP in high LD ($r^2>0.5$) with at least one archaic SNP of the introgressed haplotype are in bold
*none introgressed haplotype was found in PNG lowlanders in this region so we reported the introgressed haplotype with the highest frequency in PNG highlanders

Table S22: pairwise comparison of the distance between archaics for the introgressed haplotype chr1:89054418-89202534 at the GBP locus

arch_1	arch_2	Absolute distance difference	nSNP	difference ratio
Denisovan	Altai Neanderthal	27	201	0.067164179
Denisovan	Vindija Neanderthal	168	201	0.417910448
Denisovan	Chagyrskaya Neanderthal	314	201	0.781094527
Altai Neanderthal	Vindija Neanderthal	155	201	0.385572139
Altai Neanderthal	Chagyrskaya Neanderthal	303	201	0.753731343
Vindija Neanderthal	Chagyrskaya Neanderthal	154	201	0.383084577

The distance difference is the sum of the number of alleles (0,1 or 2) by which two archaics differ at each SNP included in the pairwise comparison
nSNP = the number of SNPs taken into the analysis; the analysis is constrained to SNPs that are called across all 4 archaics
ratio difference = absolute difference/2*nSNP

Supplementary Notes

1. Sampling and sequencing

a. Sampling (with phenotypes)

For this paper, we sequenced 204 PNG individuals. These individuals were sequenced from saliva samples collected in three places in Papua New Guinea (PNG) between 2016 and 2019 (Supplementary Data 1-2). DNA was extracted from saliva samples with the Oragene sampling kit (DNA Genotek Inc., Ottawa, Canada) according to the manufacturer's instructions. Sequencing libraries were prepared using the TruSeq DNA PCR-Free HT kit (Illumina, Inc., San Diego, California, USA). About 150-bp paired-end sequencing was performed on the Illumina HiSeq X5 sequencer (Illumina, Inc., San Diego, California, USA). We also added 58 previously published PNG whole genome sequences (Brucato et al., 2021). These samples (n=262) are distributed between three sampling places, as shown below:

- **PNG highlanders** (N=60): sampled in three different PNG highlands villages located on the slope of Mount Wilhelm (Chimbu Province): Womatne (2,300 m a.s.l.), Gembogl (2,500 m a.s.l.) and Keglsugl (2,700 m a.s.l.). (EGAD00001010143, EGAD00001010142)
- **PNG lowlanders** (N=80): living below 100 m a.s.l. in Daru (Western Province) (EGAD00001010143, EGAD00001010142, EGAD50000000050).
- **PNG diversity I** dataset (N=122): individuals sampled in Port Moresby originating from various parts of PNG (EGAD00001010142, (Brucato et al., 2021).

b. Kept Sequences

We detected six related pairs (Supplementary Note 5). When one individual of the related pair had a different number of phenotype measurements, we kept the individual with the highest number of phenotype measurements. Otherwise, we kept the individual with the highest mean coverage (Supplementary Note 4). We removed four mislabelled sequences from Mt Wilhelm. One sequence from Daru was removed before the variant calling because of bad metrics (Supplementary Note 4). Finally, we removed two individuals with a low call rate (Supplementary Note 4).

c. Additional datasets (without phenotypes)

To these 262 PNG genomes, we added 81 published Papuan genomes (PNG diversity II) (Bergström et al., 2020; Jacobs et al., 2019; Malaspinas et al., 2016; Mallick et al., 2016; Vernot et al., 2016) and genomes from Yoruba in Ibadan, Nigeria (YRI, n=108), Esan in Nigeria (ESN, n=99), British in England and Scotland (GBR, n=91), Utah Residents with Northern and Western European ancestry (n=99), Han Chinese in Bejing (CHB=103), China Kinh in Ho Chi Minh City, Vietnam (KHV, n=99) (Byrska-Bishop et al., 2022). To better describe the genetic structure of the studied populations from PNG, we also added the Australian genomes of the SGDP project (n=2) (Mallick et al., 2016), genomes from Flores island from the East Island South-East Asia (East ISEA) (n=46) (Jacobs et al., 2019) and finally samples from the Mentawai and Nias Islands, in the West ISEA (n=25) (Jacobs et al., 2019).

2. Phenotype data collection

We chose the anthropometric and physiologic measurements based on reported adaptive mechanisms in highland populations (Beall 2013; Bigham and Lee, 2014). We explained the measurements to the study participants in detail. During the collection of the phenotypes, the participants were in the rested state, without shoes. We published these phenotype measurements as a part of our dataset (André et al., 2021). We had at least one phenotype measurement and age at the measurements for 234 of the 249 kept sequenced individuals (Table S1). We didn’t have phenotype measurements for ten sequenced individuals (PNG lowlanders = 2, Port Moresby = 6, PNG highlanders = 2) and no age information for five individuals from Port Moresby. We didn’t include these individuals in the association analysis (Supplementary Note 13).

3. Variant Calling

Sequencing data for all samples used in this study were processed together, starting from the raw reads. FASTQ files were trimmed with fastp v0.23.2 (Chen et al., 2018) using the default options, except for reads shorter than 35bp were discarded (-l 35), and auto-detection of adapters for paired-end sequence data was enabled (--detect_adapter_for_pe). Unpaired reads were removed. Filtered FASTQ files were converted to BAM using Picard Tools FastqToSam v2.26.2 (*broadinstitute/picard*, 2014/2022). Further processing was performed with Broad Institute’s GATK v4.2.0.0 Germline short variant discovery (SNPs and Indels) Best Practices (Poplin et al.,

2018). HaplotypeCaller tool was used to produce individual sample GVCF files using Whole Genome Germline Single Sample v2.3.2 Cromwell workflow. The JointGenotyping workflow v1.5.1 was used to produce multi-sample VCF from GVCF files obtained in the first step. Data were processed with GRCh38 genome reference built into the GATK Germline short variant discovery (SNPs + Indels) workflow https://gatk.broadinstitute.org/hc/en-us/articles/360035535932-Germline-short-variant-discovery-SNPs-Indels.

4. Quality control

One sample from PNG lowlands (O517_DA_C001KMQ) was not included in the variant calling because the fraction of bases that attained at least 1X sequence coverage in post-filtering bases (PCT_1X) was only 19%. We computed mean coverage for the new sequences from PNG using the CollectWgsMetrics tool from Picardtools v2.26.3 within the autosomes interval (Supplementary Data 2, Figure S1). The average mean coverage for all the PNG samples was 18.49x.

We computed the heterozygosity and call rate of every sequence using plink (v1.9) missing and het options. One PNG highlander (O517_DA_C001KNS) sequence and one PNG lowlander (191121_FD09254770) sequence were outliers for the call rate, with a call rate lower than 92%. We removed these two individuals from further analysis (Figure S2). We computed additional quality metrics with bcftools stat for all the SNPs of all the individuals included in this paper (Supplementary Data 3).

5. Kinship analysis

We used KING v2.2.4 integrated relationship inference (Manichaikul et al., 2010) to detect pairs of first and second-degree relatives among our dataset using the option --related --degree 2. Within the 262 sequenced individuals, we detected six pairs of related individuals: 4 pairs of second-degree relatives (PNG highlanders = 1, PNG lowlanders = 3), one PNG lowlander parent-offspring pair and one full sibling pair from PNG diversity set I.

We kept the individual with the highest mean coverage from each pair (Supplementary Note 4). When two individuals from PNG diversity set I, Daru or Mt Wilhelm, shared kinship but had a different number of phenotype measurements, we kept the individuals with the highest number of phenotype measurements (Supplementary Data 1).

6. Generation of genomic mask

Previous publications (Stern et al., 2019) used the 1000 Genomes Project genomic mask to indicate the sites with low accessibility to next-generation sequencing methods using short reads (Byrska-Bishop et al., 2022). Because the 1000 Genomes Project genomic masks are based on low coverage, and our dataset is based on higher coverage (both for high coverage 1000 Genomes Project data and our PNG data set), we computed a more adapted genomic mask.

Using cram files for the unrelated PNG highlanders, PNG lowlanders, PNG diversity set I individuals and 1000 Genomes Project populations of our dataset, we called the sites with a minimum base quality of 20, an alignment minimum mapping quality of 20 and downgrading mapping quality for reads containing excessive mismatches with a coefficient of 50. From these sites, we excluded indels, sites with more than two alleles, sites with a maximum missing rate of 5% and sites that weren't included in the mappability mask from MSMC (liftover to GR38) (Schiffels & Durbin, 2014). We also masked sites whose depth of coverage summed across all samples was higher or lower than the sum of the average depth across the dataset (24184.7) by a factor of 2-fold (below 12092.35 or above 48369.4). We wrote the remaining sites P (the base passed all filters), except for variants without the PASS flag from the variant calling.

7. LD pruning and Admixture

To prune variants in high linkage disequilibrium, we used PLINK v.1.9 using the default parameters of 50 variants count window shifting from five variants and a variance inflation factor (VIF) threshold of 2 (Purcell et al., 2007). We ran ADMIXTURE v1.3 (Alexander et al., 2009) for the components K=2 to K=10 on the same dataset as the PCA. ADMIXTURE computes the cross-validation error for each component using a k-fold cross-validation procedure. We set the k parameter to 100. To assess the fit of each model generated, we generated the cross-validation error ten times for each component. We then defined the confidence interval of the cross-validation error for the component using the quantile of the ten generated cross-validation errors. The confidence interval of the cross-validation error will be indicated as the following: (CI, 0.025 and 0.975 quantiles) for each component (Figure S4). We

used pong 1.5 (Behr et al., 2016) to generate the ADMIXTURE ancestry proportions plots.

8. Phasing

We phased sequences from Daru, Mt Wilhelm, PNG diversity set I and African, European and Asian populations from the 1000 Genomes Project dataset. We used the software shapeit4 (v4.2.2) (Delaneau et al., 2019). Because a reference appropriated to Papuans doesn't exist, we phased the samples statistically without reference. We used the genetic map provided by Eagle (Loh et al., 2016). This phasing was performed per chromosome using the sequencing option that adjusts the default parameters for sequenced data. In order to evaluate the phasing error, we computed the percentage of switches for individuals of the 1000 Genomes Project in our phased dataset that have phasing for trio available (CEU, n=1; YRI, n=2, KHV, n=2) (The 1000 Genomes Project Consortium et al., 2015). The average switch percentage in our phased dataset for these five individuals is 1.41%, which falls in the switch error range observed for the phased data published with the 1000 Genomes Project for which the majority of SNPs, which fall in the MAF > 5% category, have an error < 2.5% (Belsare et al., 2019).

9. Random sampling approach to test the significance of the top candidate regions for selection

We used a random sampling approach (Skoglund et al., 2017) to test the significance of the top 10 windows with the highest score for each of the three selection scores. We looked at whether the score of the unit used to determine the score of the genomic region – SNP for XP-EHH or 20-SNP windows for PBS and Fisher score – with the highest score in the region was significantly higher than the score of random units along the genome. For each selection test, we defined random units along all the autosomes so that they are separated by at least 5Mb and thus remain independent. For XP-EHH, we compared the score of the top SNP in each candidate region to the distribution of the XP-EHH score of individual random SNPs. For the PBS and Fisher scores, the random distribution was built using random sliding windows of 20 SNPs defined in the PBS approach. In order for the random distribution to be approximately normal, we transformed the PBS and Fisher scores with the log10 transformation method. We compared the score (or the log10 of the score) of the sliding windows with the highest score (or its log10) for each of the top

10 candidate regions to the distribution of the PBS or Fisher score of the random sliding windows (Figure S5). Finally, we computed a Z score for each candidate region (Z(x)) using the mean (μ) and standard deviation for the score of the random regions (σ) and the score of the candidate region (fx). $Z(x)= (fx - \mu)/\sigma$ (Figure S6 and S7).

10. Control for the difference in mean coverage

Due to the uneven distribution of mean depth of coverage in our sample interval (Supplementary Data 2, Figure S1), we decided to check for the impact of including individuals with such higher mean coverage. We performed the XP-EHH and PBS selection scans on PNG highlanders and lowlanders while we have reduced the coverage for the 15 outliers (which are all PNG lowlanders) with a mean of coverage >25 to 30% of their previous coverage (which roughly makes all the samples with very similar coverages). In order to limit the influence of any other parameters, we performed four new independent variant callings, two of which were used for the XP-EHH scans and two of which were used for the PBS scans. For XP-EHH, the first variant calling includes all the PNG highlanders and PNG lowlanders used in the initial XP-EHH selection scan. The second variant calling includes the same individuals with the 15 outlier lowlanders whose mean coverage has been reduced. We then followed the same phasing and XP-EHH selection protocol described in the manuscript. We see a huge correlation between the two XP-EHH scores (Spearman coeff=0.965) (Figure S8). Moreover, we plot the SNPs in the 1st percentile in the first XP-EHH scan (including high coverage lowlanders) and the SNPs in the 1st percentile in the second XP-EHH scan (with 15 outliers lowlanders with reduced coverage). Most of our candidate regions for selection in PNG lowlanders include SNP in the 1st percentile in the two XP-EHH scans. In order to test the impact of higher coverage individual on the PBS scan, we performed two similar variant calling – with the 15 outliers with their initial mean of coverage or reduced coverage — including this time the YRI from I000 Genomes that we use as an outgroup. We also observe a very strong correlation between PBS scores when using the high-coverage lowlanders or the reduced-coverage lowlanders (Spearman coeff=0.998) (Figure S9). Overall, these additional analyses support the idea that the PNG lowlanders with higher coverage have a limited impact on our selection scan results.

11. Control for admixture in PNG lowlanders

We ran admixture for K=8 using the same dataset as described in Supplementary Note 6, using the bootstrapping procedure included in the admixture software, using the default number replicate of 200. This allowed us to estimate the parameter standard errors. We observed that the average admixture from PNG highlanders to PNG lowlanders is 3.28% (SE=1.86%). In order to determine whether this admixture level might affect our selection scores results, we computed the XP-EHH and PBS scores while excluding the 21 PNG lowlander individuals that had an admixture level from PNG highlanders above 5%, and we observed that there is a strong correlation (spearman coefficient > 0.92) between these new results and the original ones performed (Supplementary Figs. 10 and 11).

12. Relate

We computed recombination trees for the phased dataset using RELATE v1.1.8 (Speidel et al., 2019). We generated Relate input files from the phased dataset following Relate manual instructions using a genomic mask we generated ourselves (Supplementary Note 6) and the ancestral genome from the Ensembl 93 release (Zerbino et al., 2018). With these inputs, we generated local trees using the default parameters for the mutation rate (1.25e-8 per generation per base pair), the effective population size (30000) and Eagle genetic map (Loh et al., 2016). We then extracted subtrees of PNG highlanders and PNG lowlanders subtrees from the generated local trees.

Using the population-specific relative subtrees, we computed the coalescence rates through time separately for PNG highlanders and PNG lowlanders. We specified the first-time interval starting from 0 to 10^2 years ago, and then the period between 10^2 and 10^7 is split into successive bins with a step of $10^{0.1}$. We used Relate default parameters of 28 years per generation, the number of cycles and the fractions of trees to be dropped.

Finally, we extracted the local trees for each SNP in the regions of interest separately for PNG highlanders and PNG lowlanders and sampled branch lengths for those trees using an MCMC-like approach by applying the relevant Relate script. The branch length of the focal SNP tree is resampled 200 times ("—num_samples 200"

option of the “SampleBranchLengths.sh” script), and each iteration result is recorded to estimate the uncertainty in the age estimate of each node.

To take the uncertainty in the branch length estimate into account, we performed this step 5 times. For each SNP of each region of interest, we computed sampled branches five times. We will run Clues for each of the five sampled branches.

In order to support the idea that our Relate set-up is appropriate, we added the Ne curves for CEU, CHB, and CEU generated with our Relate set-up (Figure S12). They are similar to the Ne curves generated in the original Relate paper (Speidel, Forest, Shi, & Myers, 2019). None such curves were available for the Papuan population or population with similar demographic history, but the Ne curves we generated for PNG highlanders and lowlanders are similar to the Ne curves generated with MSMC2 (Schiffels & Wang, 2020) by Brucato et al. (Brucato et al., 2021) on other PNG individuals.

13. Clues

We used the CLUES (Stern et al., 2019), an approximate full-likelihood method for inferring selection, on each of the regions of interest previously characterized with XP-EHH, PBS and Fisher Scores. This is to detect the SNP, which is the most likely to drive selection in each region.

CLUES uses the local trees generated for each SNP with RELATE (Supplementary Note 11) in the regions of interest. The CLUES method will give each SNP the ratio of the likelihood of the SNPs being under selection – with a coefficient of selection computed by Clues – versus the likelihood of the SNP being under a neutral model. We computed the likelihood ratio for the five branch lengths generated for each SNP and averaged the five likelihood ratios. Finally, for each region, we selected the top five SNPs with the highest average likelihood ratios and generated branch lengths and logLR 50 additional times for these SNPs to further fine map for the causal SNP (Tables S9-S12). Because SNPs with low DAF are unlikely to be under positive selection, we didn’t consider SNPs with DAF lower than 5%.

In order to control if our set-up is able to pinpoint the candidate SNP under selection for already known adaptation scenarios, we generated five independent runs for every SNP within the MCM6/LCT locus (chr2: 135820191- 135876443). We reproduce the results from the original Clues paper (Stern et al., 2019) by showing

that the SNP with the highest logLR average is for rs4988235 – (chr2:135850976) (average logLR)= 22.7596, average s= 0.00718), a SNP of the MCM6 gene regulating the LCT and associated to the lactase persistence phenotype in Europeans (Smith et al., 2009).

14. Association in the UK Biobank

When looking for phenotypes significantly associated with the candidate SNPs in the UK Biobank, we considered the 1900 phenotypes for which the sampling size was higher than 10,000 and for which the p-value was computed within the European population (pval_EUR). We excluded from those the phenotypes related to diet (categories 100090 and 100052), sociodemographic factors (category 100062), sexual factors (category 100056), social support (category 100061), electronic use (category 100053) and the local environment (category 114) because of the absence of correspondence between the environmental variable described within these categories and the environment to which the studied PNG populations are exposed.

15. Association test with PNG phenotypes

We used Genome-wide Efficient Mixed Model Association GEMMA (v0.98.4) (Zhou & Stephens, 2012) to detect if the SNPs likely to be under selection in PNG highlanders or PNG lowlanders are associated with any of the phenotypes we measured in the PNG population. We first created a centred relatedness matrix for all the unrelated PNG individuals for which we had at least one phenotype measurement (n=234) (Table S2). To correct for population structure, we performed GEMMA univariate Linear Mixed Model (LMM), including this relatedness matrix. As we did in our previous study (André et al., 2021), we corrected height, diastolic pressure, systolic pressure, heart rate and haemoglobin for age and gender using a multiple linear regression approach. As height can be a covariable to some of these phenotypes, we also regressed chest width, waist circumference, FEV1, PEF and FVC, correcting for age, gender and height. For each phenotype, we performed the GEMMA LMM for the SNPs of interest and the phenotype residuals with the relatedness matrix to control for population structure. Because we are performing the test for several SNPs, we corrected for multi-testing with the Benjamini-Hochberg procedure (Benjamini & Hochberg, 1995) for FDR < 5%. We corrected the p-value following the equation from (Yekutieli & Benjamini, 1999). For each phenotype, we multiply each p-value (one per SNP tested for the phenotype) per the total number of SNPs tested for the phenotype

and divide it by the p-value rank order (smallest p-value for the phenotype = 1). We are also testing multiple phenotypes that gather in five groups of phenotypes highly correlated to each other (André et al., 2021) because the adjusted p-value should be considered significant when lower than 0.01 (0.05/5).

16. Introgression

When the candidate SNP for the region under selection was in LD (r^2>0.5) with at least one of the archaic SNPs of the introgressed haplotype, we built median-joining haplotype networks using Popart ver. 1.7 (Leigh & Bryant, 2015). In detail, starting from the phased vcf, we generated the nexus input files required by Popart using a custom script, first transforming the vcf in fasta, harnessing the scikit.allel python module, and considering 5kbp down- and upstream the variant of interest. Next, we built the median-joining network with default settings (epsilon=0) (Figure S15-S18).

In addition, to explore further adaptive introgression for specific introgressed haplotypes, we used haplostrips v1.3 (Marnetto & Huerta-Sánchez, 2017) within PNG, African, Asian and European samples with Altai (Prüfer et al., 2014), Vindija (Prüfer et al., 2017), Chagyrskaya (Mafessoni et al., 2020) Neanderthal or Denisovan (Meyer et al., 2012) genome as reference haplotypes (Figure S15). We first subset Neanderthal and Denisovan positions for the positions in our PNG and 1000 Genomes Project dataset with bcftools view v.1.14 (Danecek et al., 2021).

We ran haplostrips for the GBP locus under selection in PNG lowlanders using the phased dataset and the extracted Denisovan or Neanderthal positions. We used the haplostrips inner join option, where only sites present in the PNG, the 1000 Genomes Project dataset, and the archaic positions were used.

References:

Alexander, D. H., Novembre, J., & Lange, K. (2009). Fast model-based estimation of ancestry in unrelated individuals. *Genome Research*, *19*(9), 1655-1664. https://doi.org/10.1101/gr.094052.109

André, M., Brucato, N., Plutniak, S., Kariwiga, J., Muke, J., Morez, A., Leavesley, M., Mondal, M., & Ricaut, F.-X. (2021). Phenotypic differences between highlanders and lowlanders in Papua New Guinea. *PLOS ONE*, *16*(7), e0253921. https://doi.org/10.1371/journal.pone.0253921

André, M., Brucato, N., Hudjasov, G., Pankratov, V., Yermakovich, D., Kreevan, R., … Ricaut, F.-X. (2022, décembre 15). Positive selection in the genomes of two Papua New Guinean populations at distinct altitude levels,Source data, https://doi.org/10.6084/m9.figshare.23695062, 2024

André, M., Brucato, N., Hudjasov, G., Pankratov, V., Yermakovich, D., Kreevan, R., … Ricaut, F.-X. (2022, décembre 15). Positive selection in the genomes of two Papua New Guinean populations at distinct altitude levels, https://github.com/mathilde999/selection-png, https://doi.org/10.5281/zenodo.10793101, 2024

Beall, C. M. (2013). Human adaptability studies at high altitude : Research designs and major concepts during fifty years of discovery. *American Journal of Human Biology*, *25*(2), 141-147. https://doi.org/10.1002/ajhb.22355

Behr, A. A., Liu, K. Z., Liu-Fang, G., Nakka, P., & Ramachandran, S. (2016). pong : Fast analysis and visualization of latent clusters in population genetic data. *Bioinformatics*, *32*(18), 2817-2823. https://doi.org/10.1093/bioinformatics/btw327

Belsare, S., Levy-Sakin, M., Mostovoy, Y., Durinck, S., Chaudhuri, S., Xiao, M., Peterson, A. S., Kwok, P.-Y., Seshagiri, S., & Wall, J. D. (2019). Evaluating

the quality of the 1000 genomes project data. *BMC Genomics*, *20*. https://doi.org/10.1186/s12864-019-5957-x

Benjamini, Y., & Hochberg, Y. (1995). Controlling the False Discovery Rate : A Practical and Powerful Approach to Multiple Testing. *Journal of the Royal Statistical Society. Series B (Methodological)*, *57*(1), 289-300.

Bergström, A., McCarthy, S. A., Hui, R., Almarri, M. A., Ayub, Q., Danecek, P., Chen, Y., Felkel, S., Hallast, P., Kamm, J., Blanché, H., Deleuze, J.-F., Cann, H., Mallick, S., Reich, D., Sandhu, M. S., Skoglund, P., Scally, A., Xue, Y., … Tyler-Smith, C. (2020). Insights into human genetic variation and population history from 929 diverse genomes. *Science*, *367*(6484). https://doi.org/10.1126/science.aay5012

Bigham, A. W., & Lee, F. S. (2014). Human high-altitude adaptation : Forward genetics meets the HIF pathway. *Genes & Development*, *28*(20), 2189-2204. https://doi.org/10.1101/gad.250167.114

Broadinstitute/picard. (2022). [Java]. Broad Institute. https://github.com/broadinstitute/picard (Édition originale 2014)

Brucato, N., André, M., Tsang, R., Saag, L., Kariwiga, J., Sesuki, K., Beni, T., Pomat, W., Muke, J., Meyer, V., Boland, A., Deleuze, J.-F., Sudoyo, H., Mondal, M., Pagani, L., Gallego Romero, I., Metspalu, M., Cox, M. P., Leavesley, M., & Ricaut, F.-X. (2021). Papua New Guinean Genomes Reveal the Complex Settlement of North Sahul. *Molecular Biology and Evolution*, *msab238*. https://doi.org/10.1093/molbev/msab238

Byrska-Bishop, M., Evani, U. S., Zhao, X., Basile, A. O., Abel, H. J., Regier, A. A., Corvelo, A., Clarke, W. E., Musunuri, R., Nagulapalli, K., Fairley, S., Runnels, A., Winterkorn, L., Lowy, E., Paul Flicek, Germer, S., Brand, H., Hall, I. M.,

Talkowski, M. E., … Xiao, C. (2022). High-coverage whole-genome sequencing of the expanded 1000 Genomes Project cohort including 602 trios. *Cell*, *185*(18), 3426-3440.e19. https://doi.org/10.1016/j.cell.2022.08.004

Chen, S., Zhou, Y., Chen, Y., & Gu, J. (2018). fastp : An ultra-fast all-in-one FASTQ preprocessor. *Bioinformatics*, *34*(17), i884-i890. https://doi.org/10.1093/bioinformatics/bty560

Danecek, P., Bonfield, J. K., Liddle, J., Marshall, J., Ohan, V., Pollard, M. O., Whitwham, A., Keane, T., McCarthy, S. A., Davies, R. M., & Li, H. (2021). Twelve years of SAMtools and BCFtools. *GigaScience*, *10*(2), giab008. https://doi.org/10.1093/gigascience/giab008

Delaneau, O., Zagury, J.-F., Robinson, M. R., Marchini, J. L., & Dermitzakis, E. T. (2019). Accurate, scalable and integrative haplotype estimation. *Nature Communications*, *10*(1), Article 1. https://doi.org/10.1038/s41467-019-13225-y

Jacobs, G. S., Hudjashov, G., Saag, L., Kusuma, P., Darusallam, C. C., Lawson, D. J., Mondal, M., Pagani, L., Ricaut, F.-X., Stoneking, M., Metspalu, M., Sudoyo, H., Lansing, J. S., & Cox, M. P. (2019). Multiple Deeply Divergent Denisovan Ancestries in Papuans. *Cell*, *177*(4), 1010-1021.e32. https://doi.org/10.1016/j.cell.2019.02.035

Leigh, J. W., & Bryant, D. (2015). popart : Full-feature software for haplotype network construction. *Methods in Ecology and Evolution*, *6*(9), 1110-1116. https://doi.org/10.1111/2041-210X.12410

Loh, P.-R., Palamara, P. F., & Price, A. L. (2016). Fast and accurate long-range phasing in a UK Biobank cohort. *Nature Genetics*, *48*(7), 811-816. https://doi.org/10.1038/ng.3571

Mafessoni, F., Grote, S., de Filippo, C., Slon, V., Kolobova, K. A., Viola, B., Markin, S. V., Chintalapati, M., Peyrégne, S., Skov, L., Skoglund, P., Krivoshapkin, A. I., Derevianko, A. P., Meyer, M., Kelso, J., Peter, B., Prüfer, K., & Pääbo, S. (2020). A high-coverage Neandertal genome from Chagyrskaya Cave. *Proceedings of the National Academy of Sciences*, *117*(26), 15132-15136. https://doi.org/10.1073/pnas.2004944117

Malaspinas, A.-S., Westaway, M. C., Muller, C., Sousa, V. C., Lao, O., Alves, I., Bergström, A., Athanasiadis, G., Cheng, J. Y., Crawford, J. E., Heupink, T. H., Macholdt, E., Peischl, S., Rasmussen, S., Schiffels, S., Subramanian, S., Wright, J. L., Albrechtsen, A., Barbieri, C., … Willerslev, E. (2016). A genomic history of Aboriginal Australia. *Nature*, *538*(7624), 207-214. https://doi.org/10.1038/nature18299

Mallick, S., Li, H., Lipson, M., Mathieson, I., Gymrek, M., Racimo, F., Zhao, M., Chennagiri, N., Nordenfelt, S., Tandon, A., Skoglund, P., Lazaridis, I., Sankararaman, S., Fu, Q., Rohland, N., Renaud, G., Erlich, Y., Willems, T., Gallo, C., … Reich, D. (2016). The Simons Genome Diversity Project : 300 genomes from 142 diverse populations. *Nature*, *538*(7624), 201-206. https://doi.org/10.1038/nature18964

Manichaikul, A., Mychaleckyj, J. C., Rich, S. S., Daly, K., Sale, M., & Chen, W.-M. (2010). Robust relationship inference in genome-wide association studies. *Bioinformatics*, *26*(22), 2867-2873. https://doi.org/10.1093/bioinformatics/btq559

Marnetto, D., & Huerta-Sánchez, E. (2017). Haplostrips : Revealing population structure through haplotype visualization. *Methods in Ecology and Evolution*, *8*(10), 1389-1392. https://doi.org/10.1111/2041-210X.12747

Meyer, M., Kircher, M., Gansauge, M.-T., Li, H., Racimo, F., Mallick, S., Schraiber, J. G., Jay, F., Prüfer, K., Filippo, C. de, Sudmant, P. H., Alkan, C., Fu, Q., Do, R., Rohland, N., Tandon, A., Siebauer, M., Green, R. E., Bryc, K., … Pääbo, S. (2012). A High-Coverage Genome Sequence from an Archaic Denisovan Individual. *Science*, *338*(6104), 222-226. https://doi.org/10.1126/science.1224344

Poplin, R., Ruano-Rubio, V., DePristo, M. A., Fennell, T. J., Carneiro, M. O., Auwera, G. A. V. der, Kling, D. E., Gauthier, L. D., Levy-Moonshine, A., Roazen, D., Shakir, K., Thibault, J., Chandran, S., Whelan, C., Lek, M., Gabriel, S., Daly, M. J., Neale, B., MacArthur, D. G., & Banks, E. (2018). *Scaling accurate genetic variant discovery to tens of thousands of samples* (p. 201178). bioRxiv. https://doi.org/10.1101/201178

Prüfer, K., de Filippo, C., Grote, S., Mafessoni, F., Korlević, P., Hajdinjak, M., Vernot, B., Skov, L., Hsieh, P., Peyrégne, S., Reher, D., Hopfe, C., Nagel, S., Maricic, T., Fu, Q., Theunert, C., Rogers, R., Skoglund, P., Chintalapati, M., … Pääbo, S. (2017). A high-coverage Neandertal genome from Vindija Cave in Croatia. *Science (New York, N.Y.)*, *358*(6363), 655-658. https://doi.org/10.1126/science.aao1887

Prüfer, K., Racimo, F., Patterson, N., Jay, F., Sankararaman, S., Sawyer, S., Heinze, A., Renaud, G., Sudmant, P. H., de Filippo, C., Li, H., Mallick, S., Dannemann, M., Fu, Q., Kircher, M., Kuhlwilm, M., Lachmann, M., Meyer, M., Ongyerth, M., … Pääbo, S. (2014). The complete genome sequence of a Neandertal from the Altai Mountains. *Nature*, *505*(7481), 43-49. https://doi.org/10.1038/nature12886

Purcell, S., Neale, B., Todd-Brown, K., Thomas, L., Ferreira, M. A. R., Bender, D., Maller, J., Sklar, P., de Bakker, P. I. W., Daly, M. J., & Sham, P. C. (2007). PLINK : A Tool Set for Whole-Genome Association and Population-Based Linkage Analyses. *American Journal of Human Genetics*, *81*(3), 559-575.

Schiffels, S., & Durbin, R. (2014). Inferring human population size and separation history from multiple genome sequences. *Nature genetics*, *46*(8), 919-925. https://doi.org/10.1038/ng.3015

Skoglund, P., Thompson, J. C., Prendergast, M. E., Mittnik, A., Sirak, K., Hajdinjak, M., Salie, T., Rohland, N., Mallick, S., Peltzer, A., Heinze, A., Olalde, I., Ferry, M., Harney, E., Michel, M., Stewardson, K., Cerezo-Roman, J., Chiumia, C., Crowther, A., … Reich, D. (2017). Reconstructing Prehistoric African Population Structure. *Cell*, *171*(1), 59-71.e21. https://doi.org/10.1016/j.cell.2017.08.049

Smith, G. D., Lawlor, D. A., Timpson, N. J., Baban, J., Kiessling, M., Day, I. N. M., & Ebrahim, S. (2009). Lactase persistence-related genetic variant : Population substructure and health outcomes. *European Journal of Human Genetics*, *17*(3), Article 3. https://doi.org/10.1038/ejhg.2008.156

Speidel, L., Forest, M., Shi, S., & Myers, S. R. (2019). A method for genome-wide genealogy estimation for thousands of samples. *Nature Genetics*, *51*(9), Article 9. https://doi.org/10.1038/s41588-019-0484-x

Stern, A. J., Wilton, P. R., & Nielsen, R. (2019). An approximate full-likelihood method for inferring selection and allele frequency trajectories from DNA sequence data. *PLOS Genetics*, *15*(9), e1008384. https://doi.org/10.1371/journal.pgen.1008384

The 1000 Genomes Project Consortium, Gibbs, R. A., Boerwinkle, E., Doddapaneni, H., Han, Y., Korchina, V., Kovar, C., Lee, S., Muzny, D., Reid, J. G., Zhu, Y., Wang, J., Chang, Y., Feng, Q., Fang, X., Guo, X., Jian, M., Jiang, H., Jin, X., … Rasheed, A. (2015). A global reference for human genetic variation. *Nature*, *526*(7571), 68-74. https://doi.org/10.1038/nature15393

Vernot, B., Tucci, S., Kelso, J., Schraiber, J. G., Wolf, A. B., Gittelman, R. M., Dannemann, M., Grote, S., McCoy, R. C., Norton, H., Scheinfeldt, L. B., Merriwether, D. A., Koki, G., Friedlaender, J. S., Wakefield, J., Pääbo, S., & Akey, J. M. (2016). Excavating Neandertal and Denisovan DNA from the genomes of Melanesian individuals. *Science (New York, N.Y.)*, *352*(6282), 235-239. https://doi.org/10.1126/science.aad9416

Yekutieli, D., & Benjamini, Y. (1999). Resampling-based false discovery rate controlling multiple test procedures for correlated test statistics. *Journal of Statistical Planning and Inference*, *82*(1), 171-196. https://doi.org/10.1016/S0378-3758(99)00041-5

Zerbino, D. R., Achuthan, P., Akanni, W., Amode, M. R., Barrell, D., Bhai, J., Billis, K., Cummins, C., Gall, A., Girón, C. G., Gil, L., Gordon, L., Haggerty, L., Haskell, E., Hourlier, T., Izuogu, O. G., Janacek, S. H., Juettemann, T., To, J. K., … Flicek, P. (2018). Ensembl 2018. *Nucleic Acids Research*, *46*(D1), D754-D761. https://doi.org/10.1093/nar/gkx1098

Zhou, X., & Stephens, M. (2012). Genome-wide efficient mixed-model analysis for association studies. *Nature Genetics*, *44*(7), Article 7. https://doi.org/10.1038/ng.2310
