# Supplementary material for: Positive selection in the genomes of two Papua New Guinean populations at distinct altitude levels
Source: Nat Commun. 2024 Apr 30;15:3352. doi: 10.1038/s41467-024-47735-1 (PMC11061283; doi:10.1038/s41467-024-47735-1)
Supplement: Supplementary file 3 — Description of Additional Supplementary Files [file 41467_2024_47735_MOESM3_ESM.pdf]

Description of Additional Supplementary Files

Supplementary Data 1: Papuan dataset of the study and which analysis they have been included.

Supplementary Data 2: Mean coverage, heterozygosity and call rate within the autosome interval for PNG diversity set I, PNG highlanders and PNG lowlanders.

Supplementary Data 3: Quality metrics for all the sequences included in the variant calling.

Supplementary Data 4: Introgressed archaic segments identified with hmmix per sample for the most frequent introgressed haplotype for the genomic region chr12:58391529-58634980 under selection in PNG highlanders.

Supplementary Data 5: Introgressed archaic segments identified with hmmix per sample for the most frequent introgressed haplotype for the genomic region chr1:88800562-89326878 under selection in PNG lowlanders.
