# Supplementary material for: Positive selection in the genomes of two Papua New Guinean populations at distinct altitude levels
Source: Nat Commun. 2024 Apr 30;15:3352. doi: 10.1038/s41467-024-47735-1 (PMC11061283; doi:10.1038/s41467-024-47735-1)
Supplement: Supplementary file 5 — Reporting Summary [file 41467_2024_47735_MOESM5_ESM.pdf]

nature portfolio

Corresponding author(s): François-Xavier Ricaut, Mayukh Mondal

Last updated by author(s): Mar 13, 2024

Reporting Summary

Nature Portfolio wishes to improve the reproducibility of the work that we publish. This form provides structure for consistency and transparency in reporting. For further information on Nature Portfolio policies, see our Editorial Policies and the Editorial Policy Checklist.

Statistics

For all statistical analyses, confirm that the following items are present in the figure legend, table legend, main text, or Methods section.

n/a	Confirmed	
☐	☒	The exact sample size (*n*) for each experimental group/condition, given as a discrete number and unit of measurement
☐	☒	A statement on whether measurements were taken from distinct samples or whether the same sample was measured repeatedly
☐	☒	The statistical test(s) used AND whether they are one- or two-sided<br>*Only common tests should be described solely by name; describe more complex techniques in the Methods section.*
☐	☒	A description of all covariates tested
☐	☒	A description of any assumptions or corrections, such as tests of normality and adjustment for multiple comparisons
☐	☒	A full description of the statistical parameters including central tendency (e.g. means) or other basic estimates (e.g. regression coefficient) AND variation (e.g. standard deviation) or associated estimates of uncertainty (e.g. confidence intervals)
☐	☒	For null hypothesis testing, the test statistic (e.g. *F*, *t*, *r*) with confidence intervals, effect sizes, degrees of freedom and *P* value noted<br>*Give P values as exact values whenever suitable.*
☒	☐	For Bayesian analysis, information on the choice of priors and Markov chain Monte Carlo settings
☒	☐	For hierarchical and complex designs, identification of the appropriate level for tests and full reporting of outcomes
☒	☐	Estimates of effect sizes (e.g. Cohen's *d*, Pearson's *r*), indicating how they were calculated

Our web collection on statistics for biologists contains articles on many of the points above.

Software and code

Policy information about availability of computer code

Data collection	No software was used for data collection
Data analysis	fastp v0.23.2, FastqToSam v2.26.2 , GATK v4.2.0.0, Cromwell workflow 2.3.2, Picardtools 2.26.3, KING v2.2.4, vcftools 0.1.14, Plink 1.9 , EIGENSOFT v.7.2.0, ADMIXTURE v1.3, R 3.3.0,python 3.7.12, Shapeit 4.2.2 , bedtools 2.29.2, selscan 2.0.0, Relate 1.1.8 , GEMMA 0.98.4, bcftools 1.14, pong 1.5, All custom codes used in this study are available on GitHub (https://github.com/mathilde999/selection-png)

For manuscripts utilizing custom algorithms or software that are central to the research but not yet described in published literature, software must be made available to editors and reviewers. We strongly encourage code deposition in a community repository (e.g. GitHub). See the Nature Portfolio guidelines for submitting code & software for further information.

Data

Policy information about availability of data

All manuscripts must include a data availability statement. This statement should provide the following information, where applicable:

- Accession codes, unique identifiers, or web links for publicly available datasets
- A description of any restrictions on data availability
- For clinical datasets or third party data, please ensure that the statement adheres to our policy

The whole genome sequences and the phenotype measurements from the 60 PNG highlanders, 80 PNG lowlanders, and 64 individuals sampled in Port Moresby generated in this study have been deposited in the European Genome-phenome Archive under accession codes EGAD00001010142 [https://ega-archive.org/

datasets/EGAD00001010142], EGAD00001010143 [https://ega-archive.org/datasets/EGAD00001010143] and EGAD50000000050 [https://ega-archive.org/datasets/EGAD50000000050]. The whole genome sequences are available under restricted access to protect the privacy of the participants, in agreement with the Institutional Review Board approval and the individuals' informed consent forms. The data are available to the scientific community under controlled access and reviewed by the Data Access Committee of the Papua New Guinean Genome Diversity Project. Access to the new whole genome sequences published with this paper is automatically granted upon request in the case of replication of the published study. New demographic, selection and association studies require approval from the Papua New Guinean Genome Project program (PNGP) committee. Source data generated in this paper can be accessed on the linked figshare repository [https://doi.org/10.6084/m9.figshare.23695062]72.
Published samples used in this study were retrieved from the European Nucleotide Archive (ENA) under the accession numbers PRJEB9586 [http://www.ebi.ac.uk/ena/data/view/PRJEB9586] and ERP010710 [http://www.ebi.ac.uk/ena/data/view/ERP010710]51 and PRJEB6463 [http://www.ebi.ac.uk/ena/data/view/PRJEB6463]49; the European Genome-phenome Archive (EGA) under the accession numbers EGAS00001001247 [https://www.ebi.ac.uk/ega/studies/EGAS00001001247]50, EGAS00001003054 [https://ega-archive.org/studies/EGAS00001003054]26 and EGAS00001005393 [https://ega-archive.org/studies/EGAS00001005393]2; the database of Genotypes and Phenotypes (dbGaP) under the accession number phs001085.v1.p1 [https://www.ncbi.nlm.nih.gov/projects/gap/cgi-bin/study.cgi?study_id=phs001085.v1.p1]39. The high-coverage genomes from the 1000 Genomes project were accessed at https://www.internationalgenome.org/data-portal/data-collection/30x-grch38 52.
The GRCh38 genome reference was built into the GATK Germline short variant discovery (SNPs + Indels) workflow. The resources required for this workflow are accessible on the following Google Cloud bucket [https://console.cloud.google.com/storage/browser/genomics-public-data/resources/broad/hg38/v0/] 55.
The Ancestral Genome for Homo sapiens (GRCh38) was retrieved from the Ensembl website [https://ftp.ensembl.org/pub/release-93/fasta/ancestral_alleles/homo_sapiens_ancestor_GRCh38/] 71. The genetic map for GRCh38 was accessed from Eagle website [https://alkesgroup.broadinstitute.org/Eagle/downloads/tables/]. UK biobank GWAS summary statistics are available at http://www.nealelab.is/uk-biobank 64.

Research involving human participants, their data, or biological material

Policy information about studies with human participants or human data. See also policy information about sex, gender (identity/presentation), and sexual orientation and race, ethnicity and racism.

Reporting on sex and gender	The participants include both males and females, and the cohort did not include or exclude participants based on their gender. In our study gender was not considered in the study design. Gender of participants was determined based on self-report.
Reporting on race, ethnicity, or other socially relevant groupings	The cohort did not include or exclude participants based on their race, ethnicity or other socially relevant groupings.
Population characteristics	Individuals were chosen to represent populations living in the lowlands (<100 m a.s.l.) and highlands (>2300 m a.s.l.) of Papua New Guinea or the diversity of the PNG population (individuals sampled in Port Moresby)
Recruitment	The cohort was collected between 2016-2019 in Daru, Mount Wilhelm and Port Moresby. All samples were colected from healty unrelated adult donors (older than 18 years old) who provided written informed consent. The participation was completely voluntary and there were no advantages or disadvantages to agree or not to participate in the study. After a full presentation of the project to a wide audience, a discussion with each individual willing to participate ensured that the project was fully understood. We did not provide any compensation to the participants. . In our research application approved by the PNG ethic authorities is mentioned that “No risk or benefit for the participant will come from our project, except the fact the participants will contribute to a research project that will bring new information on Papua New Guinea population history.” Considering that the aim of our study was to access the genetic diversity at each site (Daru and Mount Wilhelm) we do not anticipate the recruitment process to have introduced major biases.
Ethics oversight	This study was approved by the Medical Research Advisory Committee of Papua New Guinea under research ethics clearance MRAC 16.21 and the French Ethics 122 Committees (Committees of Protection of Persons CPP 25/21_3, n_SI : 123 21.01.21.42754). Permission to conduct research in PNG was granted by the National Research Institute (visa n° 99902292358) with full support from the School of Humanities and Social Sciences, University of Papua New Guinea. Community engagement preceded sample collection and all participants provided consent for population genetic studies.

Note that full information on the approval of the study protocol must also be provided in the manuscript.

Field-specific reporting

Please select the one below that is the best fit for your research. If you are not sure, read the appropriate sections before making your selection.

☒ Life sciences ☐ Behavioural & social sciences ☐ Ecological, evolutionary & environmental sciences

For a reference copy of the document with all sections, see nature.com/documents/nr-reporting-summary-flat.pdf

Life sciences study design

All studies must disclose on these points even when the disclosure is negative.

Sample size	The new cohort used in this study includes 81 samples collected between 2016-2019 from Lowland (Daru) and highland (Mount Wilhelm). In this study, the sample size was limited by the availability of samples from each location. We also gained access to PNG whole genome sequences from 123 samples collected at the same sampling places during the same period and sequenced at the National Center of Human Genomics Research (France) or the KCCG Sequencing Laboratory (Garvan Institute of Medical Research, Australia). The number of individuals sampled in each of the three regions (Mt Wilhelm, Daru and Port Moresby) was chosen on a trade-off to run Population Genetics Selection scans and Genotype-Pehnotype Association tests on candidate genes. As the effect size of the trait of interest (related to altitude adaptation)

	could not be known beforehand: 1/ we first selected a number of individuals classically used in Population Genetics studies (n>50)(Brucato et al https://doi.org/10.1016/j.isci.2022.104583 has sample size between 45 and 54 samples, Malaspinas et al 2016, 30 for each population, etc) and combine them with published datasets (1000 Genomes Project Consortium doi: 10.1038/nature15393 and the Simons Genome Diversity Project https://doi.org/10.1038/nature18964. 2/ Then we defined a set of regions of interest based on the selection scans, optimising our statistical power. Because genetic variants under selection are by definition common, we assumed that a sample size of few hundreds would be efficient to detect signals association between gentoypes under selection and phenotypes related to altitude, as done in other works on altitude in Tibet (Beall et al 2010)
Data exclusions	Data exclusion was based on standard criteria such as sequencing quality (call rate, coverage, etc) and relatedness between individuals. The following paragraph is include in the « supplementary notes, 1. Sampling and sequencing, b.Kept sequences » : We detected six related pairs (Supplementary Note 6). When one individual of the related pair had a different number of phenotypes measurements, we kept the individual with the highest number of phenotype measurements. Or else we kept the individual with the highest mean coverage (Supplementary Note 4). We removed four mislabelled sequences from Mt Wilhelm. One sequence from Daru was removed before the variant calling (Supplementary Note 3). Finally, we removed two individuals with a low call rate (Supplementary Note 4).
Replication	There was no need for replication due to the nature of this population genetic study. The association study was not replicated because of no other PNG dataset was available. However given our rational, the study can be replicated.
Randomization	Samples were grouped based on their geographic location (lowland, highland). The samples were clustered into three groups (Mt Wilhelm, Daru and Port Moresby) depending of the sampling location, and after checking their genealogy (up to the third generation) to establish local ancestry. Some samples were further excluded based on quality control and kinship. Allocation was not random but based on anthropological data. This allocation was necessary as our aim of the study was to find differences between these groups.
Blinding	No blinding methods were implemented as they were not necessary for our study. We have generated genetic diversity data based on the location of the samples collection. Blinding was not necessary for the genetic data analysis run in this study, as anonymising (requested by the ethics committee) the samples was sufficient for our study protocol.

Reporting for specific materials, systems and methods

We require information from authors about some types of materials, experimental systems and methods used in many studies. Here, indicate whether each material, system or method listed is relevant to your study. If you are not sure if a list item applies to your research, read the appropriate section before selecting a response.

Materials & experimental systems

n/a	Involved in the study
☒	☐ Antibodies
☒	☐ Eukaryotic cell lines
☒	☐ Palaeontology and archaeology
☒	☐ Animals and other organisms
☒	☐ Clinical data
☒	☐ Dual use research of concern
☒	☐ Plants

Methods

n/a	Involved in the study
☒	☐ ChIP-seq
☒	☐ Flow cytometry
☒	☐ MRI-based neuroimaging

Plants

Seed stocks	*Report on the source of all seed stocks or other plant material used. If applicable, state the seed stock centre and catalogue number. If plant specimens were collected from the field, describe the collection location, date and sampling procedures.*
Novel plant genotypes	*Describe the methods by which all novel plant genotypes were produced. This includes those generated by transgenic approaches, gene editing, chemical/radiation-based mutagenesis and hybridization. For transgenic lines, describe the transformation method, the number of independent lines analyzed and the generation upon which experiments were performed. For gene-edited lines, describe the editor used, the endogenous sequence targeted for editing, the targeting guide RNA sequence (if applicable) and how the editor was applied.*
Authentication	*Describe any authentication procedures for each seed stock used or novel genotype generated. Describe any experiments used to assess the effect of a mutation and, where applicable, how potential secondary effects (e.g. second site T-DNA insertions, mosiacism, off-target gene editing) were examined.*
